# Ugi-Tetrazole-Derived α‑Aminomethyl Scaffolds Reveal Unexpected Binding Modes in SARS-CoV‑2 3CLpro

**DOI:** 10.1021/acsmedchemlett.5c00773

**Published:** 2026-03-04

**Authors:** Robin van der Straat, Rick Oerlemans, Yingying Cong, Jeffrey Boxma, Radu G. Bulai, Clàudia Río-Bergé, Lizbé Koekemoer, Tryfon Zarganes Tzitzikas, Zhirui Guan, Peter George Marples, Fulvio Reggiori, Matthew Groves, Alexander Dömling

**Affiliations:** ‡ Department of Medicinal Chemistry, Photopharmacology and Imaging, Groningen Research Institute of Pharmacy, 225118 University of Groningen, 9713 AV Groningen, Netherlands; § Department of Chemical and Pharmaceutical Biology, Groningen Research Institute of Pharmacy, University of Groningen, 9713 AV Groningen, Netherlands; ∥ Department of Biomedicine, 1006 Aarhus University, 8000 Aarhus, Denmark; ⊥ Department of Drug Design, Groningen Research Institute of Pharmacy, University of Groningen, 9713 AV Groningen, Netherlands; # Centre for Medicines Discovery, Nuffield Department of Medicine, 6396 University of Oxford, Oxford OX3 7FZ, United Kingdom; ∇ Diamond Light Source, Limited, Harwell Science and Innovation Campus, Didcot OX11 0DE, United Kingdom; ○ Research Complex at Harwell, Harwell Science and Innovation Campus, Didcot OX11 0FA, United Kingdom; ◆ Innovative Chemistry Group, Regional Centre of Advanced Technologies and Materials, Czech Advanced Technology and Research Institute (CATRIN), Palacký University Olomouc, Šlechtitelů 27, 783 71 Olomouc, Czech Republic; ¶ Institute of Molecular and Translational Medicine, Faculty of Medicine and Dentistry, Palacký University and University Hospital Olomouc, 779 00 Olomouc, Czech Republic

**Keywords:** 3CLpro inhibitor, multicomponent reaction, Ugi, 1,5-disubstituted tetrazole, SARS-CoV-2

## Abstract

The SARS-CoV-2 main protease (3CLpro) is a well-validated target for structure-guided inhibitor discovery. Here, we report α-aminomethyl tetrazole inhibitors accessed via the Ugi tetrazole multicomponent reaction (UT-4CR), enabling rapid exploration of non-classical chemical space. Initial design and modeling suggested a binding mode analogous to Ugi-derived (U-4CR) 3CLpro inhibitors, with heteroaromatic substituents engaging the S1 pocket. However, crystallographic analysis revealed an unexpected binding orientation in which the tetrazole core itself occupies the S1 pocket and forms the key interaction with His163, while the modeled substituents are solvent-exposed. This revised binding mode rationalizes the observed structure–activity relationships. Installation of an electrophilic warhead yielded covalent inhibitors with sub-micromolar enzymatic potency, and lead compound **2a** displayed modest antiviral activity in infected cells. These results highlight UT-4CR-derived tetrazoles as a platform for probing the 3CLpro binding space and underscore the importance of early crystallographic validation.

## Graphical abstract

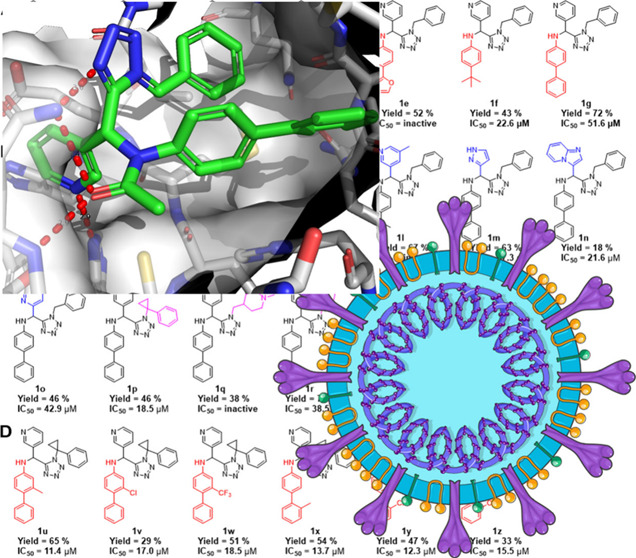

## Introduction

The coronavirus main protease (3CLpro) is a cysteine protease that has emerged as a benchmark target for structure-guided inhibitor discovery, owing to its well-defined active site, high conservation across coronaviruses, and extensive structural characterization (Figure 1).^1–3^ Several peptidomimetic scaffolds were described as 3CLpro inhibitors, and nirmatrelvir was developed for COVID-19 treatment in a record time (Figure 1A).^4^ Another seminal contribution to this area was the identification of ML188 (Figure 1B), a noncovalent 3CLpro inhibitor accessible in a single step via the Ugi four-component reaction (U-4CR).^5^ This work demonstrated that multicomponent reactions (MCRs) can deliver synthetically efficient access to structurally complex protease inhibitors while maintaining precise engagement of defined substrate-binding pockets. Subsequent efforts established U-4CR chemistry as a practical platform for rapid hit identification and structure-guided optimization against 3CLpro.^6–10^ Nevertheless, U-4CR-derived bisamide scaffolds, such as ML188, suffer from intrinsic limitations, including restricted scaffold diversity and suboptimal physicochemical properties, which have constrained further development. These challenges motivated exploration of whether scaffold morphing within the Ugi reaction scaffold manifold could preserve key binding interactions while accessing an alternative chemical space with distinct structural and electronic features. Among available Ugi variants, the Ugi tetrazole reaction (UT-4CR) provides direct access to α-aminomethyl tetrazoles, a heterocyclic motif with unique hydrogen-bonding characteristics, altered polarity, and high synthetic convergence.^11^ Importantly, UT-4CR products maintain a topological arrangement of substituents comparable to that of classical U-4CR adducts, suggesting that tetrazole-based analogues could emulate known 3CLpro binding modes while simultaneously probing new interaction patterns (Figure 1C). Here, we report a scaffold-morphing strategy that translates the original U-4CR-derived 3CLpro inhibitor framework into a tetrazole-based architecture using UT-4CR chemistry. Rather than pursuing incremental potency optimization, this study was designed to interrogate how tetrazole incorporation alters protease binding behavior. By integrating synthesis, enzymatic evaluation, crystallography, and cellular assays, we uncover a binding-mode inversion in which the tetrazole core itself engages the S1 pocket of 3CLpro, deviating from the originally modeled design. These findings highlight both the opportunities and limitations of model-driven design for non-classical MCR-derived scaffolds and underscore the importance of early structural validation in scaffold-morphing approaches.

**Figure 1:**
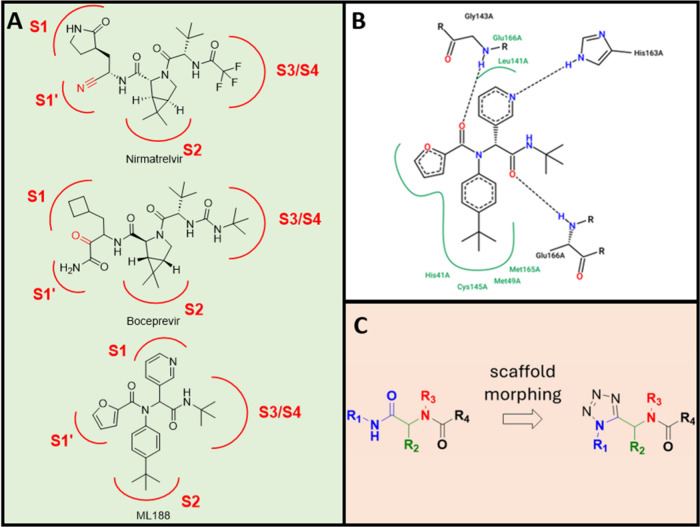
Background and design of the study. (A) Peptidometic inhibitors against SARS-CoV-2 3CLpro and their decomposition into the 3CLpro sub-binding sites (S1–S4). (Top) Marketed drug Paxlovid comprises the active ingredient nirmatrelvir. (Middle) Repurposed FDA-approved hepatitis C virus drug boceprevir. (Bottom) Structure of U-4CR compound ML188. The electrophile inactivating active site Cys145 is highlighted in red. (B) PoseView^12^ two-dimensional diagram of ML188 in the 3CLpro binding site indicating the main interactions. (C) Scaffold morphing of the U-4CR-derived ML188 scaffold to the novel UT-4CR-derived tetrazole scaffold, with the colors indicating the same components and similar 3D conformations.

## Design and Modeling of Tetrazole-Based 3CLpro Inhibitors

Our design strategy was guided by the hypothesis that scaffold morphing within the Ugi reaction manifold could preserve the overall topology of established 3CLpro inhibitors while accessing chemically distinct binding behavior (Figures 1C and 2). As a reference framework, we used the U-4CR-derived inhibitor ML188, whose binding mode has been extensively characterized crystallographically and serves as a benchmark for noncovalent 3CLpro inhibition (Figure 1B). In ML188 and related analogues, a heteroaromatic substituent occupies the S1 pocket and forms a conserved hydrogen bond with His163 (Figure 2F), while hydrophobic groups extend into the S2 and S4 subsites.

**Figure 2:**
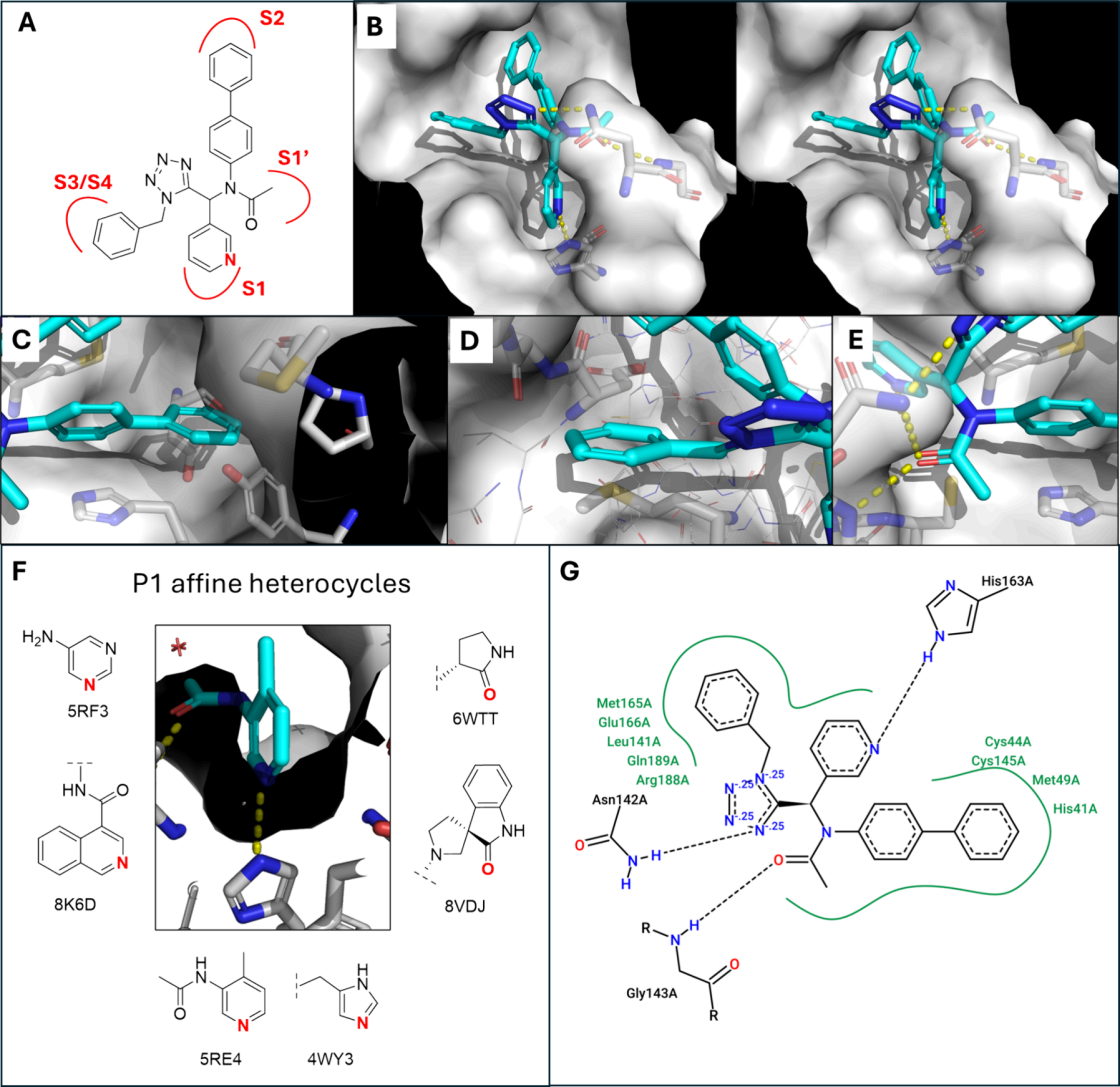
Scaffold design and modeling. (A) Representative UT-4CR tetrazole structure and it is fit into the different 3CLpro pockets. (B) Overall stereoview of the modeling/docking into the 3CLpro receptor site (PDB ID 7KX5).^14^ Hydrogen bonds are indicated as yellow dotted lines. For clarity reasons, the majority of receptor amino acids are not shown. (C) Close-up view of the S2 occupancy. (D) Close-up view of the S3 and S4 occupancy. (E) Close-up view of the S1′ occupancy. (F) Selection of co-crystallized heterocycles occupying S1 and forming a hydrogen bond with His163 and their corresponding PDB IDs. The bold red atom indicates the hydrogen bond acceptor position. (G) PoseView^12^ two-dimensional diagram of the modeled tetrazole (A) in the 3CLpro binding site indicating major interactions.

The Ugi tetrazole reaction (UT-4CR) generates 1,5-disubstituted α-aminomethyl tetrazoles while retaining three of the four input components of classical U-4CR. Subsequent acylation of the secondary amine introduced by UT-4CR completes the fourth component, yielding a scaffold that closely mirrors the substitution pattern and overall topology of U-4CR products. Nevertheless, replacement of the bis­(amide) core with a tetrazole introduces a defined geometric change that alters the relative orientation of the isocyanide substituent directed toward the S3/S4 pocket. In the classical U-4CR backbone, the secondary amide adopts a *trans* configuration, whereas the 1,5-disubstituted tetrazole functions as a *cis*-amide isostere. However, structural comparison of U-4CR and UT-4CR products indicates that substituents derived from the aldehyde, amine, and isocyanide components occupy similar three-dimensional positions despite this *trans* to *cis* geometric shift. This suggested that tetrazole analogues could emulate the ML188 binding topology while introducing altered polarity and hydrogen-bonding properties.

Molecular modeling was therefore performed using MOLOC software by replacing the bisamide core of ML188 with a tetrazole moiety while maintaining analogous substituent mapping onto the 3CLpro subsites (Figure 2).^13^ Docking was used primarily to guide the initial library design rather than to predict definitive binding modes. In these models, aldehyde-derived heteroaromatic substituents were predicted to engage the S1 pocket through hydrogen bonding with His163 (Figure 2B), while aniline-derived substituents were oriented toward the hydrophobic S2 pocket (Figure 2C), favoring bulky aromatic groups, such as biphenyl systems. The isocyanide-derived substituent was positioned toward the S3/S4 region (Figure 2D), which is known to tolerate structural diversity. The secondary amine generated by UT-4CR was oriented toward the oxyanion hole, suggesting that the electrophilic extension at this position could enable covalent engagement of catalytic Cys145 (Figure 2E).

Based on these assumptions, a focused library of tetrazole derivatives was designed to systematically probe the S1, S2, and S3/S4 subsites while maintaining synthetic efficiency. Heteroaromatic aldehydes were selected to test S1 hydrogen-bonding capacity; substituted biphenyl and *tert*-butylphenyl amines were employed to address the hydrophobic S2 pocket; and diverse isocyanides were incorporated to modulate the polarity and steric occupancy of solvent-exposed regions. In parallel, selected compounds were designed for the late-stage installation of electrophilic warheads at the secondary amine, enabling direct comparison of noncovalent and covalent inhibition modes.

Importantly, this design phase assumed that the tetrazole scaffold would act primarily as a structural linker, preserving the ML188-like binding orientation of the peripheral substituents (Figure 2G). As described below, crystallographic analysis revealed a markedly different binding mode, necessitating reinterpretation of the observed structure–activity relationships.

## Structure–Activity Relationships of Tetrazole-Based 3CLpro Inhibitors

### Chemistry-Guided Library Design

The tetrazole inhibitor series was assembled by using the Ugi tetrazole multicomponent reaction (UT-4CR), enabling rapid and convergent access to 1,5-disubstituted α-aminomethyl tetrazoles from readily available building blocks (Scheme 1). Owing to the limited commercial availability of suitably substituted anilines, several required amine components were synthesized via Suzuki cross-coupling reactions (Scheme 2). This chemistry allowed independent variation of four substituent vectors, derived from the amine, aldehyde, and isocyanide components, as well as late-stage modification of the secondary amine while maintaining a constant central scaffold. To evaluate the inhibitory potency of the generated racemic tetrazole compounds, we first determined their IC_50_ value using an *in vitro* 3CLpro enzymatic activity assay. The proteolytic activity of purified recombinant SARS-CoV-2 3CLpro was measured by a fluorescence resonance energy transfer (FRET) assay. The cleavage of the peptidic FRET substrate 2-aminobenzoyl-SVTLQSG-Tyr­(NO_2_)-R was monitored, and IC_50_ values for all compounds were determined as detailed in the Supporting Information. The dose–response curves and associated confidence intervals can be found in Figure S3. As a result, focused structure–activity relationship (SAR) studies could be performed efficiently by systematically modulating substituents intended to address individual subsites of 3CLpro and introducing electrophilic warheads to probe covalent inhibition (Figure 4).

**Scheme 1:**
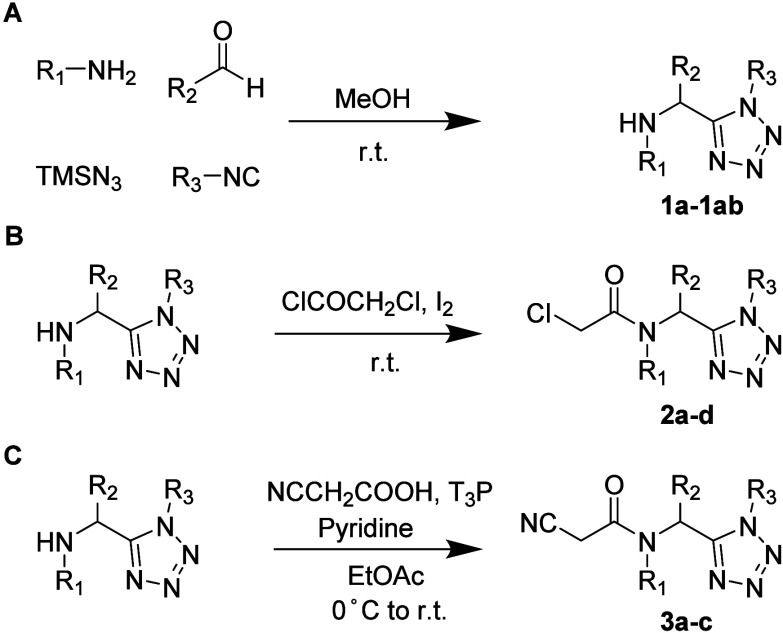
(A) Synthesis of 1,5-Disubstituted Tetrazole **1a**–**1ab**, (B) Iodine-Catalyzed Acylation toward Chloroacetamide **2a**–**2d**, and (C) Amide Coupling toward Cyan Acetamide **3a**–**3c**

**Scheme 2:**
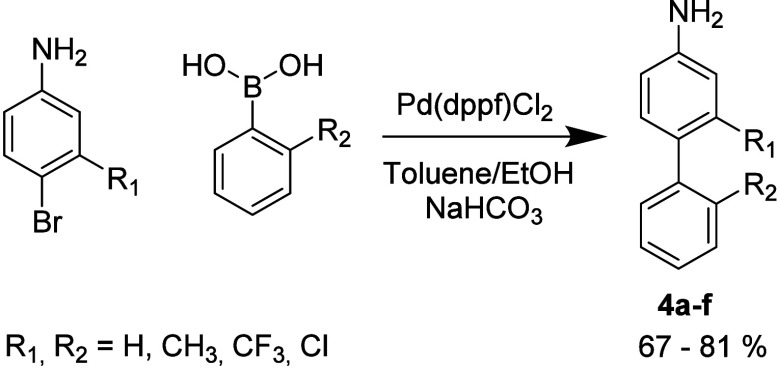
Suzuki Cross-Coupling toward [1,1′-Biphenyl]-4-amines^a^

a Reagents and conditions: Pd­(dppf)­Cl_2_ and toluene/ethanol/NaHCO_3_ (saturated aqueous, 5:1:5, 0.3 M) at 85 °C for 12 h.

**Figure 3:**
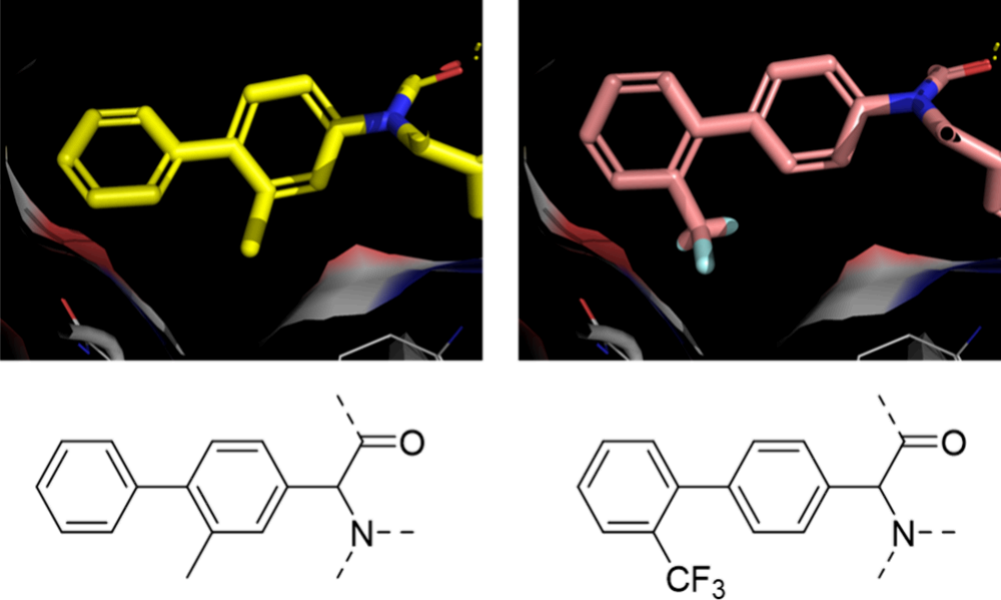
Close-up view of modeled substituted biphenyl inhibitors in the S2 pocket. Both *ortho* positions of the biphenyl ring are strategically positioned to interact with and effectively fill a small subpocket within S2, potentially enhancing binding affinity through steric complementarity and optimized hydrophobic interactions.

**Figure 4:**
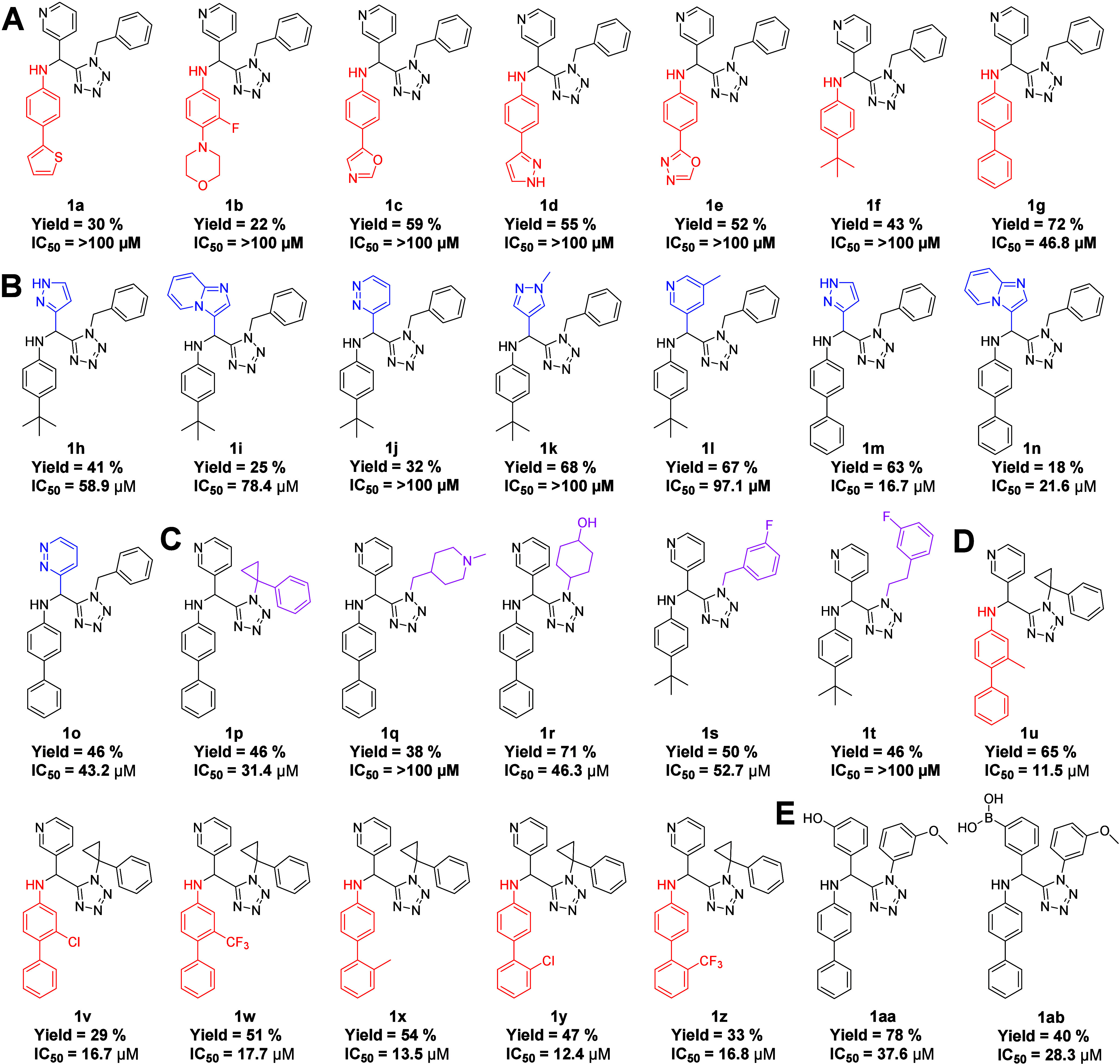
1,5-Disubstituted 5-aminomethyl tetrazole scaffold of noncovalent SARS-CoV-2 3CLpro inhibitors. (A) Analogues with S2 modifications. (B) Analogues with S1 modifications. (C) Analogues with S3/S4 modifications. (D) Analogues with S2 modifications optimizing the small subpocket. (E) Analogues with combined S1 and S3/S4 modifications.

### Modulation of the S2 Pocket

Variation of the aniline-derived substituent, designed to occupy the hydrophobic S2 pocket, revealed a clear preference for extended aromatic systems. Biphenyl-containing derivatives consistently displayed superior potency relative to *tert*-butylphenyl or heteroaromatic analogues, affording low-micromolar inhibition in enzymatic assays. These results are consistent with favorable hydrophobic and π-stacking interactions within the S2 pocket.

Further refinement through *ortho* substitution of the biphenyl motif produced modest but reproducible improvements in potency, consistent with improved steric complementarity and conformational preorganization within this pocket (Figure 3).

### Probing the S1 and S3/S4 Regions

In contrast, SAR trends associated with aldehyde-derived heteroaromatic substituents and isocyanide-derived groups are comparatively shallow. While modest differences in potency were observed across this series, no clear correlation with the predicted S1 hydrogen-bonding capacity or S3/S4 occupancy emerged. Diverse substituents with varying sizes and polarities were generally well-tolerated, suggesting limited or indirect engagement of these regions in the dominant binding mode.

### Transition to Covalent Inhibition

To probe proximity to catalytic Cys145 and enhance inhibitory potency, selected noncovalent tetrazoles were functionalized at the secondary amine with electrophilic warheads (Figure 5). Introduction of chloroacetamide moieties resulted in a pronounced increase in activity, yielding sub-micromolar IC_50_ values. In contrast, cyanoacetamide analogues were inactive under the same conditions, highlighting the importance of both electrophile reactivity and geometric alignment within the active site. Relative potency trends among the covalent inhibitors did not strictly parallel those of their noncovalent precursors, indicating that covalent engagement imposes additional spatial constraints that override incremental substituent effects observed in the parent series. Overall, the observed SAR revealed a dominant contribution from S2-directed hydrophobic interactions and covalent engagement of Cys145, while variations targeting the presumed S1 and S3/S4 regions exerted unexpectedly weak effects on potency. As shown below, crystallographic analysis provides a structural basis for these findings.

**Figure 5:**
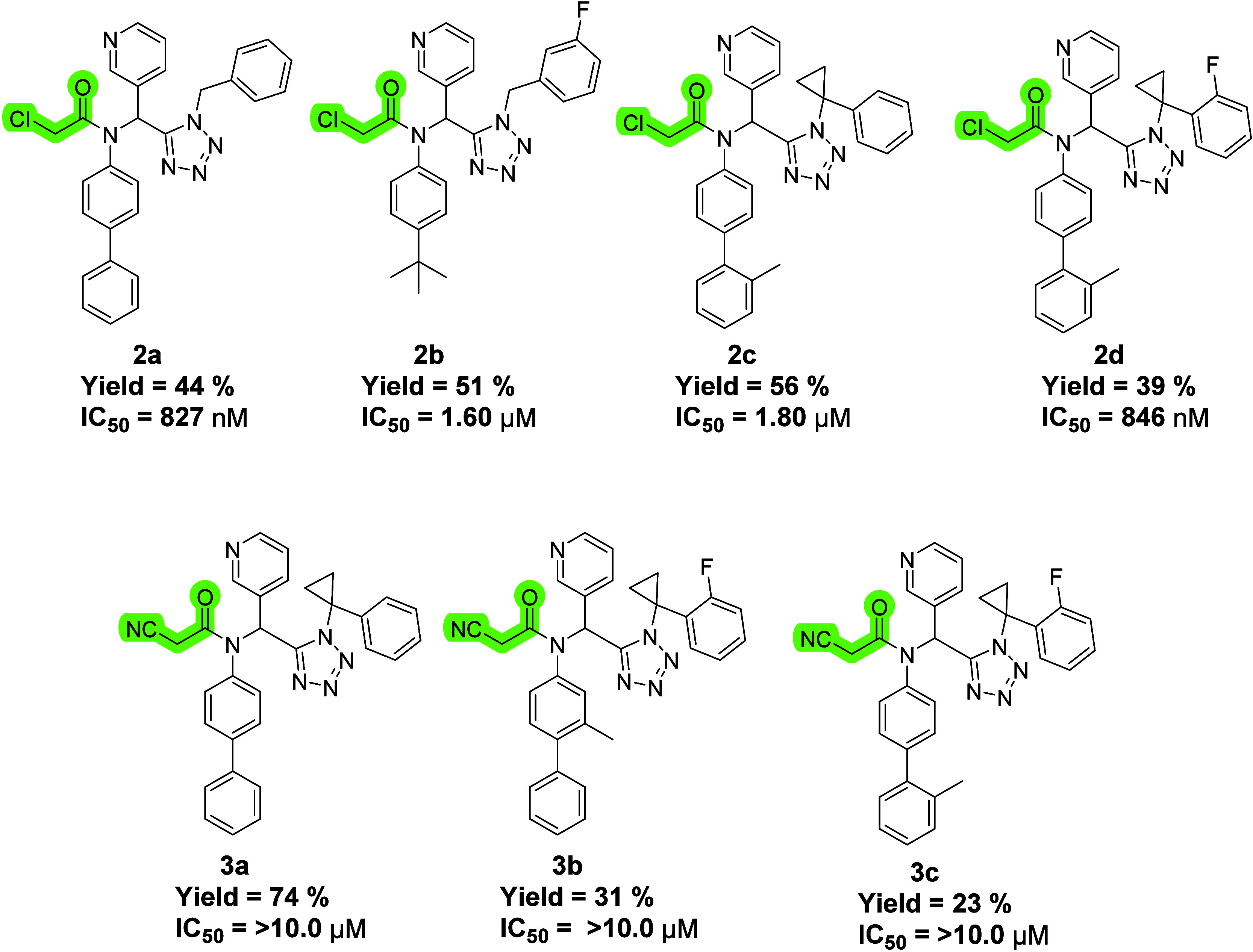
1,5-Disubstituted 5-aminomethyl tetrazole scaffold of SARS-CoV-23 CLpro inhibitors with a chloroacetamide warhead (**2a**–**2d**) or cyan acetamide (**3a**–**3c**)

### Crystallographic Analysis and Revised Binding Mode Interpretation

To elucidate the molecular basis of the unexpectedly shallow SAR observed for substituents originally designed to address the S1 and S3/S4 subsites, co-crystallization experiments were performed with representative tetrazole inhibitors. Crystals suitable for structure determination were obtained for compound **2a**, and the structure of the SARS-CoV-2 3CLpro–**2a** complex was solved at 2.04 Å resolution (Figure 6 and Table S1). The crystal structure reveals a covalent bond between electrophilic chloroacetamide of **2a** and catalytic Cys145, confirming the intended mode of covalent inhibition (Figure 6B). Acetamide carbonyl is positioned within the oxyanion hole, forming stabilizing hydrogen bonds with the backbone amides of Gly143 and Ser144, consistent with the productive engagement of the catalytic machinery (Figure 7A–C). These interactions rationalize the pronounced increase in potency observed upon installation of the chloroacetamide warhead. Strikingly, the overall binding orientation of **2a** deviates substantially from the design model (Figure 7D). Contrary to initial docking predictions, the aldehyde-derived heteroaromatic substituent does not occupy the S1 pocket and is instead largely solvent-exposed. Instead, the tetrazole core itself penetrates deeply into the S1 subsite, forming a hydrogen bond with His163. This interaction positions the tetrazole ring as the primary S1 anchoring element of the scaffold, effectively replacing the role typically played by heteroaromatic substituents in classical U-4CR-derived inhibitors, such as ML188. This binding-mode inversion provides a structural explanation for the previously observed SAR trends. The weak dependence of inhibitory activity on aldehyde-derived heterocycles and isocyanide substituents is consistent with their limited engagement in the experimentally observed binding pose. In contrast, the pronounced sensitivity to modifications of the biphenyl moiety is readily rationalized by its deep insertion into the hydrophobic S2 pocket, where it forms π-stacking and van der Waals interactions with His41, Met165, and surrounding residues. A comparison of the crystallographic structure with the original modeled pose highlights that the tetrazole scaffold actively dictates ligand orientation through its heterocyclic nitrogen array and hydrogen-bonding properties (Figure 7D). Rather than acting as a passive linker, the tetrazole functions as a dominant pharmacophoric element that replaces the classical heteroaromatic S1 anchor. This behavior is not readily predicted by conventional docking approaches calibrated on bisamide-based inhibitors. Together, these data reconcile the design hypothesis with the experimental SAR and underscore the importance of early structural validation when applying scaffold-morphing strategies to non-classical heterocycles.

**Figure 6:**
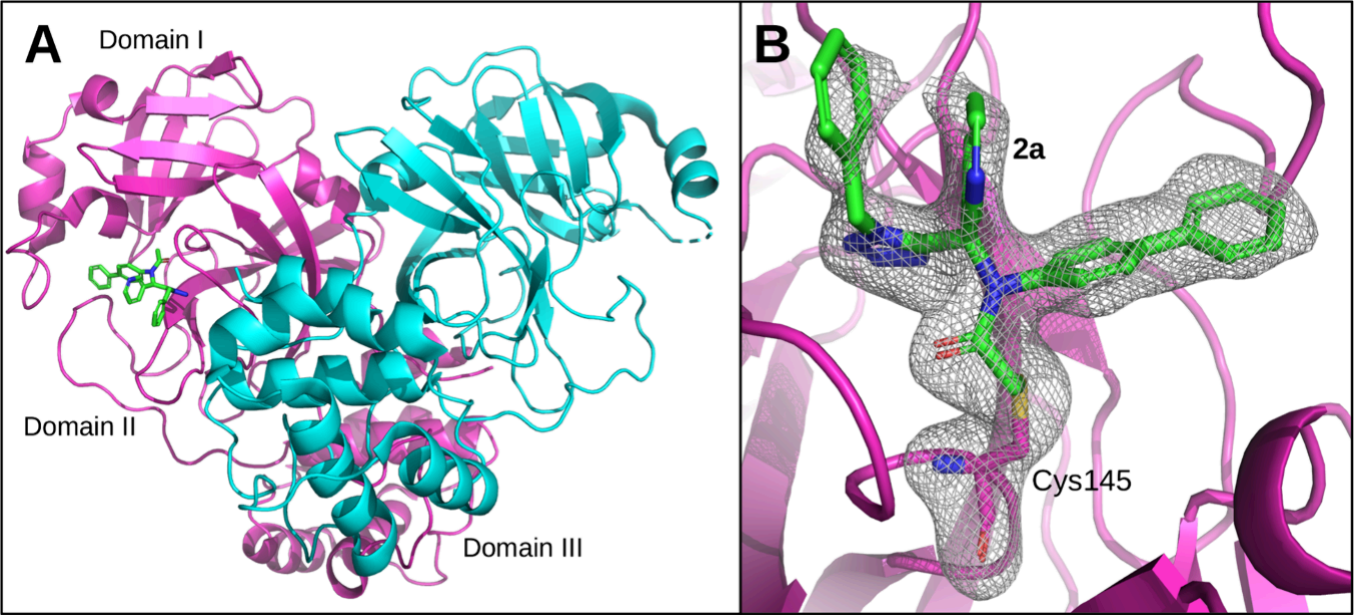
Co-crystal structure of **2a** with SARS-CoV-2 3CLpro. (A) Overall dimeric 3CLpro topology (chain A, magenta; chain B, cyan) with domains indicated on chain A. Domain I (residues 8–99), domain II (residues 100–183), and domain III (residues 201–303). (B) **2a** (green sticks) covalently bound to Cys145 in the active site of 3CLpro with polder map density contoured at 3σ in gray mesh.

**Figure 7:**
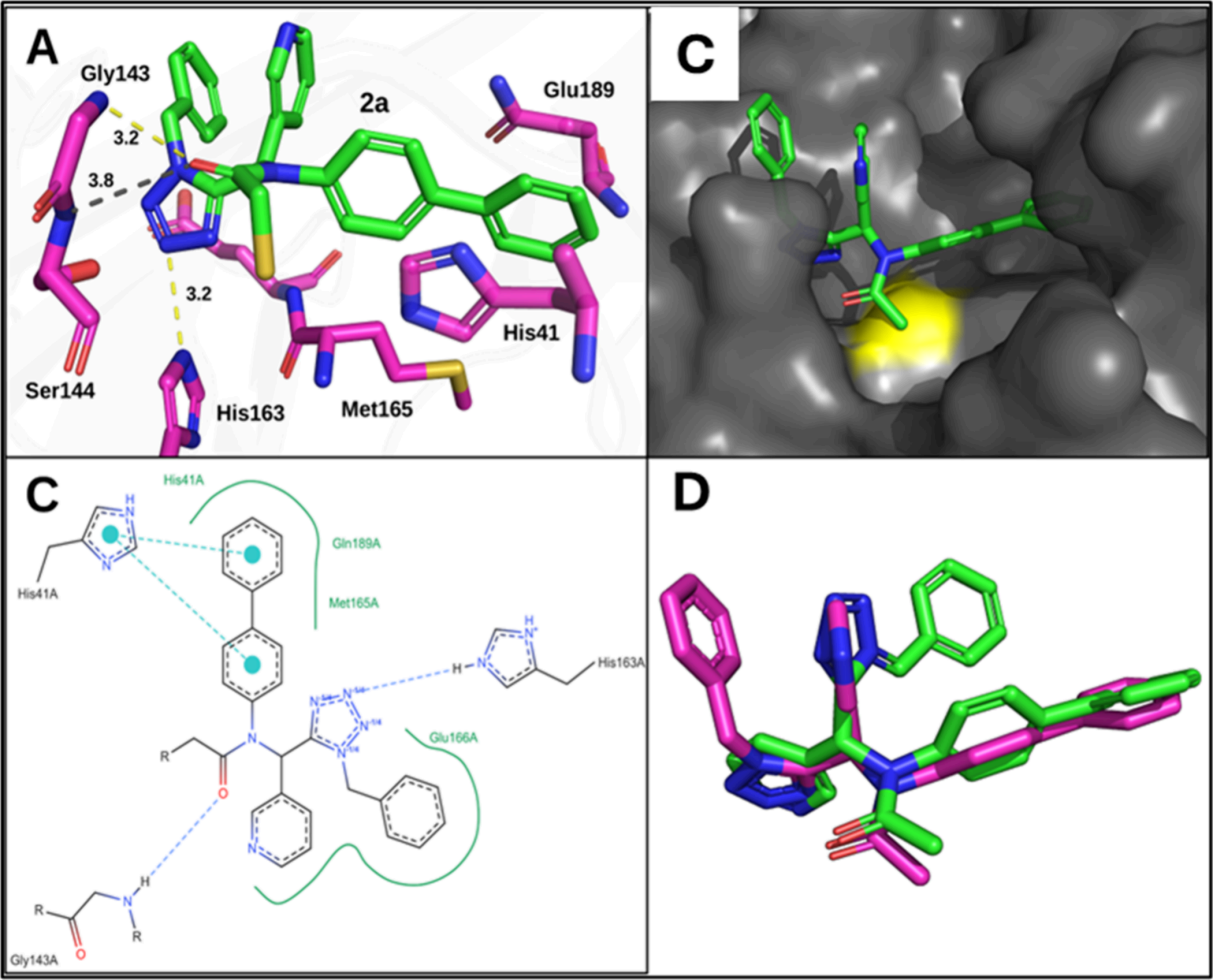
Active site details of 3CLpro with **2a**. (A) Interactions of **2a** (green sticks) with active site residues of 3CLpro (magenta sticks). Hydrogen bonds are represented as dashed yellow lines with distances indicated. The dashed gray line represents a close polar contact. (B) Gray surface representation of 3CLpro bound with **2a** (green sticks). Catalytic Cys145 is shown in yellow. (C) PoseEdit two-dimensional diagram of co-crystallized **2a** in the 3CLpro binding site indicating the main interactions. (D) Alignment of **2a** (magenta sticks) and modeled compound (*R*)-**1** (green sticks).

### Cellular Evaluation of the Lead Tetrazole Inhibitor

We further evaluated the inhibition effects of model compound **2a** on the replication of the live SARS-CoV-2 virus. To this aim, we first perform a cytotoxic assay of **2a** in A549-hACE2 cells to determine the maximal non-toxic concentration of it that could be used in cells by MTT, and we did not observe the obvious cytotoxicity at a concentration up to ∼2 μM (Figure 8A). We further investigate the antiviral effect of **2a** at concentrations from 0.125 to 2 μM, with a serial dilution factor of 2. A549-hACE2 cells were infected with SARS-CoV-2 at MOI 1 as described in the Supporting Information, and **2a** was added at 1 h post-infection (hpi). The incubation continued for an extra 23 h before processing the cells to assess viral infection by different approaches. We first evaluated the viral gRNA and viral N gene mRNA expression by RT-PCR. As a positive control, we used bafilomycin A1 (BafA1), an inhibitor of the lysosomal H^+^-ATPase that increases the pH in the compartments of the endolysosomal system, blocking the cell entry of SARS-CoV-2. BafA1 was therefore added at the same time as **2a**, and as expected, BafA1 showed a strong reduction of SARS-CoV-2 mRNA expression levels, both gRNA and N gene mRNA expression. Moreover, **2a** showed a reduction of viral mRNA expression, approximately 50–60% at a concentration between 0.5 and 2 μM and still a 20% reduction at 0.25 μM, in comparison to the DMSO-treated infected cells (Figure 8B–D), indicating that **2a** has a weak dose-dependent manner on inhibiting SARS-CoV-2 infection. Next, we assessed viral protein expression via western blot (WB) with the SARS-CoV-2 N protein upon **2a** treatments. The viral N protein expression level showed a reduction of 50–60% at a concentration between 0.25 and 2 μM and still 30% at 0.125 μM compared to DMSO-treated infected cells (Figure 8E and F). Moreover, we also collected the cell culture supernatants from the WB assay to titrate the progeny virus by TCID50 to explore whether this could further enhance the observed effects of **2a**. As shown in Figure 8G, 0.125 μM **2a** treatment, which is the lowest concentration used in our setup, reduced the SARS-CoV-2 egression by approximately 60%, and this is similar to other concentrations of **2a** treatments. Taken together, our results show that **2a** has an evident antiviral effect against SARS-CoV-2 at concentrations ranging from 0.125 to 2 μM.

**Figure 8:**
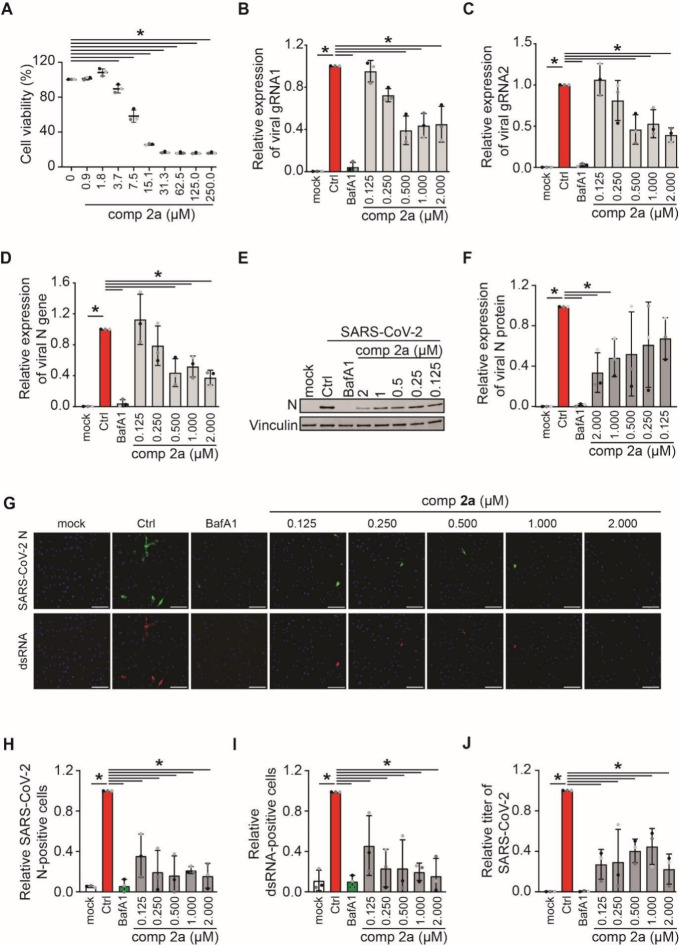
Compound **2a** in SARS-CoV-2 infection. (A) Cytotoxicity of **2a** in A549-hACE2 cells. Cells were treated with the indicated doses of **2a** for 24 h before measuring the cell viability using a MTT assay. (B–J) A549-hACE2 cells were infected with SARS-CoV-2 at MOI 1 for 1 h before **2a** or BafA1 treatment for another 23 h. Controls were cells not exposed to SARS-CoV-2 (mock), incubated with SARS-CoV-2 only (ctrl), or both SARS-CoV-2 and BafA1 (BafA1). The replication of SARS-CoV-2 was measured by assessing the expression levels of (B) gRNA1, (C) gRNA2, and (D) N gene by RT-PCR and normalizing to those encoding for GAPDH. Results are expressed relative to the ctrl condition. (E) Protein expression of the SARS-CoV-2 N protein was measured by WB using antibodies against the SARS-CoV-2 N protein and vinculin. (F) N protein expression in each sample was quantified and normalized to the vinculin signal. (G) Cells were stained with antibodies against the SARS-CoV-2 N protein and dsRNA, and (H and I) number of infected cells was quantified by immunofluorescence. (J) Cell supernatants were collected, and the viral titers were determined by the TCID50 assay. Scale bars = 100 μm. Results are quantified relative to the ctrl. All quantifications represent the average of three experiments plus standard deviation. Asterisks indicate significant differences, with a *p* value of <0.05.

## Discussion

This study explored whether scaffold morphing within the Ugi reaction manifold could provide access to an alternative chemical space for 3CLpro inhibition while retaining the synthetic efficiency that has made U-4CR-derived inhibitors attractive starting points. By translating a classical bisamide U-4CR framework into a tetrazole-based architecture using UT-4CR, we accessed a structurally distinct scaffold that enabled rapid SAR exploration of both covalent and noncovalent inhibitors. A central finding is that incorporation of the tetrazole moiety fundamentally alters binding behavior in ways not readily predictable by conventional docking approaches. While the initial design assumed heteroaromatic substituents would engage the S1 pocket in analogy to ML188, crystallographic analysis revealed that the tetrazole core itself serves as the dominant S1-binding element through direct interaction with His163. This binding-mode inversion provides a unifying explanation for the observed SAR, including the weak dependence of potency on substituents originally intended to address the S1 and S3/S4 subsites and the dominant influence of S2-directed hydrophobic interactions. Importantly, the tetrazole scaffold is not a passive isostere of the bisamide linkage but functions as an active pharmacophoric element that dictates the ligand orientation within the active site. This observation highlights a broader consideration for scaffold-morphing strategies: heterocycles introduced to expand chemical space or improve physicochemical properties may introduce non-intuitive interaction patterns that override design assumptions derived from parent scaffolds. In such cases, early structural validation is essential to avoid misleading SAR interpretations. The successful conversion of selected tetrazoles into covalent inhibitors further underscores the utility of this scaffold. Installation of a chloroacetamide warhead enabled productive engagement of catalytic Cys145, resulting in sub-micromolar enzymatic potency and measurable antiviral activity in a cellular infection model. While the cellular efficacy of compound **2a** remains modest, it demonstrates that the revised binding mode is compatible with target engagement in cells and provides a foundation for future optimization. In summary, this work illustrates how scaffold morphing within multicomponent chemistry can both expand accessible chemical space and challenge established structure-based design paradigms. The combination of UT-4CR synthesis with early crystallographic analysis provides a general framework for discovering and correctly interpreting non-classical binding modes in protease inhibitor discovery.

## Supplementary Material



## Notes

Experimental procedures, characterization, NMR spectral data for compounds **1a**–**1ab**, **2a**–**2d**, **3a**–**3c**, and **4a**–**4f**, X-ray crystallographic structure determination for compound **2a**, and IC_50_ values for all compounds (PDF)

## Abbreviations Used

3CLpro: 3C-like protease
BafA1: bafilomycin A1
FRET: Förster resonance energy transfer
GAPDH: glyceraldehyde-3-phosphate dehydrogenase
MCR: multicomponent reaction
MOI: multiplicity of infection
MTT: 3-(4,5-dimethylthiazol-2-yl)-2,5-diphenyltetrazolium bromide
SAR: structure–activity relationship
SARS: severe acute respiratory syndrome
SARS-CoV-2: severe acute respiratory syndrome coronavirus 2
U-4CR: Ugi four-component reaction
UT-4CR: Ugi tetrazole four-component reaction
