# Supplementary material for: Ugi-Tetrazole-Derived α‑Aminomethyl Scaffolds Reveal Unexpected Binding Modes in SARS-CoV‑2 3CLpro
Source: ACS Med Chem Lett. 2026 Mar 4;17(4):856–65. doi: 10.1021/acsmedchemlett.5c00773 (PMC13071645; doi:10.1021/acsmedchemlett.5c00773)
Supplement: Supplementary file 1 [file ml5c00773_si_001.pdf]

Supporting Information

Ugi-Tetrazole–Derived α-Aminomethyl Scaffolds Reveal Unexpected Binding Modes in SARS-CoV-2 3Clpro

Robin van der Straat ‡1, Rick Oerlemans ‡2, Yingying Cong‡3, Jeffrey Boxma[4], Radu G. Bulai[4], Clàudia Río-Bergé[3], Lizbé Koekemoer[5], Tryfon Zarganes Tzitzikas[5], Zhirui Guan[7], Peter George Marples[6], Fulvio Reggiori[3], Matthew Groves[2], Alexander Dömling[7,8]*

[1]Department of Medicinal Chemistry, Photopharmacology and Imaging, Groningen Research Institute of Pharmacy, University of Groningen, 9713 AV Groningen, The Netherlands
[2]Department of Chemical and Pharmaceutical Biology, Groningen Research Institute of Pharmacy, University of Groningen, 9713 AV Groningen, The Netherlands
[3]Department of Biomedicine, Aarhus University, Aarhus, Denmark
[4]Department of Drug Design, Groningen Research Institute of Pharmacy, University of Groningen, 9713 AV Groningen, The Netherlands
[5]Centre for Medicines Discovery, Nuffield Department of Medicine, University of Oxford, Oxford, United Kingdom
[6]Diamond Light Source Ltd., Harwell Science and Innovation Campus, Didcot, United Kingdom, Research Complex at Harwell, Harwell Science and Innovation Campus, Didcot, United Kingdom.
[7]Innovative Chemistry Group, Regional Centre of Advanced Technologies and Materials, Czech Advanced Technology and Research Institute (CATRIN), Palacký University Olomouc, Šlechtitelů 27, Olomouc, 783 71, Czech Republic.
[8]Institute of Molecular and Translational Medicine, Faculty of Medicine and Dentistry, Palacký University and University Hospital Olomouc, Olomouc, Czech Republic.

†corresponding author
*E-mail: alexander.domling@upol.cz

Table of Contents

General information 3

Experimental procedures and analytical data 4

NMR spectra 18

Chiral separation……………………………………………………………………...56

Protein expression and purification for the purpose of activity assays 56

Activity assay 56

Protein expression and purification for the purpose of crystallisation 57

Crystallisation 57

Cell culture and the cytotoxic assay………………………..………………………………58

Virus stocks and titration……………………………………………………………..59

Antiviral assays…………………………………………………………………….59

Statistical analyses…………………………………………………………………59

Curves activity assay……………………………………………………………….60

References 62

General information

All formamides were prepared in house by performing the formylation reaction of primary amine with ethyl formate[1,2]. Isocyanides were prepared according to our earlier described procedure[3]. Other reagents and solvents were purchased from Sigma Aldrich, Abcr GmbH, Acros, Fluorochem, A2B Chem and AK Scientific and were used without further purification. Nuclear magnetic resonance spectra (NMR) were recorded on a Bruker Avance 500 spectrometer ^{1}H NMR (500 MHz), ^{13}C NMR (126 MHz)). Chemical shifts for 1H NMR were reported relative to TMS (δ 0 ppm) or internal solvent peak ($CDCl_3$ δ 7.26 ppm, DMSO-*d6* δ 2.50 ppm or CD_3OD δ 3.31 ppm) and coupling constants were in hertz (Hz). The following abbreviations were used for spin multiplicity: s = singlet, d = doublet, t = triplet, dd = double doublet, m = multiplet, brs = broad singlet. Chemical shifts for ^{13}C NMR reported in ppm relative to the solvent peak ($CDCl_3$ δ 77.16 ppm, DMSO-*d6* δ 39.52 ppm, CD_3OD δ 49.00 ppm). Thin layer chromatography was performed on precoated silica gel 60 F254 plates (Merck, Darmstadt). Silica Gel used for purification was purchased from Screening Devices b.v.. Reagents were available from commercial suppliers and used without any purification unless otherwise noted. Yields given refer to isolated and spectroscopically pure compounds unless otherwise stated. Melting points were determined using an OEM Electrothermal melting point apparatus 1A 8103. High-resolution mass spectra were recorded using a QTOF Bruker Maxis Plus, mass range 100-1500 m/z, spectra rate 2.00 Hz

Experimental procedures and analytical data

<u>Procedure A</u>: General procedure for the Ugi tetrazole reaction 1a-z

A mixture of aldehyde (1.0 eq.), amine (1.0 eq.) in MeOH (1.0 M) was placed in a glass vial equipped with a magnetic stirring bar. After 15 minutes of stirring $TMSN_3$ (1.5 eq.) followed by isocyanide (1 eq.) was added to the mixture. This mixture was stirred for 24 hours at room temperature. Evaporation of the solvents was followed by purification by silica gel flash chromatography using PE-EA as eluent to obtain the corresponding products **1a-z.**

<u>Procedure B</u>: General procedure for chloroacetamides 2a-d

Ugi tetrazole product was transferred to a 4 mL vial and dissolved in chloroacetyl chloride. A catalytic amount of iodine was added and the reaction mixture was stirred for 5 hours. Upon completion, the reaction was quenched with a sat. solution of sodiumthiosulfate. The organic layer was washed with a sat. solution of $NaHCO_3$, dried over anh. $MgSO_4$, filtered over a small plug of silica eluting with EtOAc and concentrated in vacuo. The oil was dissolved in a few drops of DCM and a mixture of petroleum ether / diethyl ether was added to collect chloroacetamides **2a-d** as a solid.

<u>Procedure C</u>: General procedure for cyanoacetamides 3a-c

Amine (1.1 eq.), cyanoacetic acid (1.0 eq.) and pyridine (3.0 eq) were dissolved in EtOAc in a 4 mL vial and cooled to -20˚C. A solution of propylphosphonic anhydride 50 wt. % in EtOAc (2.0 eq.) was dropwise added to the reaction mixture and was stirred for 20 hours at room temperature. The reaction mixture was cooled down to 0˚C and quenched using 0.5M HCl and stirred for 2 hours at room temperature. DCM was added and the organic layer was washed twice with brine. The organic layer was dried over anh. $MgSO_4$ and concentrated in vacuo. The oil was dissolved in a few drops of DCM and a mixture of petroleum ether / diethyl ether was added to collect cyanoacetamides **3a-c** as a solid.

<u>Procedure D</u>: General procedure for [1,1'-biphenyl]-4-amines 4a-f

A mixture of amine (1 eq.) and Boronic acid (1.5 eq.) in toluene:ethanol:sat. aq. sodium bicarbonate solution (5:1:5 , 0.3M) was placed under nitrogen and degassed for 10 minutes. [1,1'-Bis(diphenylphosphino)ferrocene]palladium(II) chloride (0.5 mol%) was added and the reaction mixture was heated at 85 °C for 12 h. Ethyl acetate and water were added to the reaction mixture. The organic phase was washed with 1M sodium hydroxide solution and brine. The organic extract was dried over magnesium sulfate and concentrated by rotatory evaporation. The residue was purified by silica gel flash chromatography using PE-EA as eluent.

N-((1-benzyl-1H-tetrazol-5-yl)(pyridin-3-yl)methyl)-4-(thiophen-2-yl)aniline (1a)

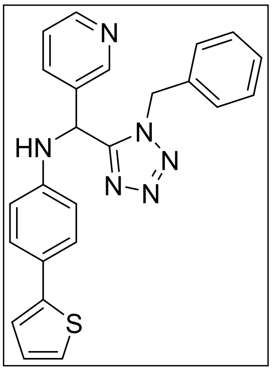

Synthesis according to procedure **A** afforded **1a** (256 mg, 0.60 mmol, 30 %) as a dark yellow solid; **mp:** 132 - 136 ˚C; **¹H NMR** (500 MHz, $CDCl_3$) δ 8.54 (dd, *J* = 4.8, 1.6 Hz, 1H), 8.45 (d, *J* = 2.4 Hz, 1H), 7.56 (dt, *J* = 7.9, 2.0 Hz, 1H), 7.42 – 7.36 (m, 3H), 7.36 – 7.32 (m, 2H), 7.23 (dd, *J* = 8.2, 4.9 Hz, 1H), 7.17 (dd, *J* = 5.1, 1.1 Hz, 1H), 7.14 – 7.10 (m, 3H), 7.01 (dd, *J* = 5.1, 3.6 Hz, 1H), 6.46 – 6.40 (m, 2H), 5.76 (d, *J* = 7.6 Hz, 1H), 5.62 (d, *J* = 15.5 Hz, 1H), 5.45 (d, *J* = 15.5 Hz, 1H), 4.78 (d, *J* = 7.9 Hz, 1H) ppm. **¹³C NMR** (126 MHz, $CDCl_3$) δ 154.7, 150.3, 148.8, 144.3, 144.3, 134.9, 132.7, 132.6, 129.6, 129.5, 129.4, 129.2, 127.9, 127.5, 127.3, 126.4, 124.1, 123.6, 121.8, 114.2, 51.6, 51.1 ppm. **HRMS** (ESI) m/z calculated for $C_{24}H_{20}N_6S$ $[M+H]^+$: 425.1549, found $[M+H]^+$: 425.1543.

N-((1-benzyl-1H-tetrazol-5-yl)(pyridin-3-yl)methyl)-3-fluoro-4-morpholinoaniline (1b)

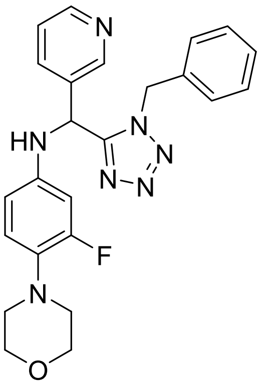

Synthesis according to procedure **A** afforded **1b** (193 mg, 0.44 mmol, 22 %) as a yellow solid; **mp:** 154 - 158 ˚C; **¹H NMR** (500 MHz, $CDCl_3$) δ 8.54 (d, *J* = 4.9 Hz, 1H), 8.42 (d, *J* = 2.6 Hz, 1H), 7.51 (dd, *J* = 6.2, 4.2 Hz, 1H), 7.43 – 7.32 (m, 3H), 7.23 (dd, *J* = 8.1, 4.7 Hz, 1H), 7.10 (d, *J* = 7.3 Hz, 2H), 6.71 (t, *J* = 9.1 Hz, 1H), 6.19 – 6.11 (m, 2H), 5.65 – 5.55 (m, 2H), 5.40 (d, *J* = 15.4 Hz, 1H), 4.58 (d, *J* = 8.4 Hz, 1H), 3.85 – 3.77 (m, 4H), 2.95 – 2.88 (m, 4H) ppm. **¹³C NMR** (126 MHz, $CDCl_3$) δ 157.6, 155.6, 154.6, 150.3, 148.8, 141.2, 141.1, 134.9, 132.8, 132.8, 132.6, 132.4, 129.5, 129.4, 127.5, 124.1, 120.2, 120.2, 109.8, 109.8, 103.2, 103.0, 67.1, 51.6, 51.5, 51.5 ppm. **HRMS** (ESI) m/z calculated for $C_{24}H_{24}FN_7O$ $[M+H]^+$: 446.2105, found $[M+H]^+$: 446.2099.

N-((1-benzyl-1H-tetrazol-5-yl)(pyridin-3-yl)methyl)-4-(oxazol-5-yl)aniline (1c)

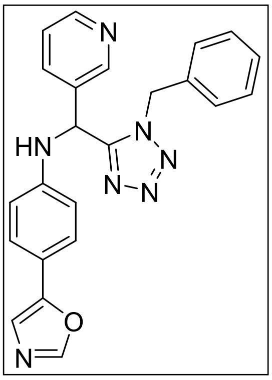

Synthesis according to procedure **A** afforded **1c** (483 mg, 1.18 mmol, 59 %) as an off-white solid; **mp:** 150 - 156 ˚C; **¹H NMR** (500 MHz, $CDCl_3$) δ 8.55 (dd, *J* = 4.9, 1.6 Hz, 1H), 8.46 (d, *J* = 2.4 Hz, 1H), 7.82 (s, 1H), 7.55 (dt, *J* = 8.0, 2.0 Hz, 1H), 7.42 – 7.34 (m, 5H), 7.24 (dd, *J* = 8.0, 4.7 Hz, 1H), 7.15 – 7.09 (m, 3H), 6.47 – 6.42 (m, 2H), 5.76 (d, *J* = 7.9 Hz, 1H), 5.63 (d, *J* = 15.5 Hz, 1H), 5.42 (d, *J* = 15.5 Hz, 1H), 4.94 (d, *J* = 7.9 Hz, 1H) ppm. **¹³C NMR** (126 MHz, $CDCl_3$) δ 154.5, 151.5, 150.4, 149.7, 148.9, 145.1, 134.9, 132.6, 132.4, 129.6, 129.5, 127.5, 125.9, 124.1, 119.7, 119.5, 114.0, 51.6, 50.8 ppm. **HRMS** (ESI) m/z calculated for $C_{23}H_{19}N_7O$ $[M+H]^+$: 410.1729, found $[M+H]^+$: 410.1724.

N-((1-benzyl-1H-tetrazol-5-yl)(pyridin-3-yl)methyl)-4-(1H-pyrazol-3-yl)aniline (1d)

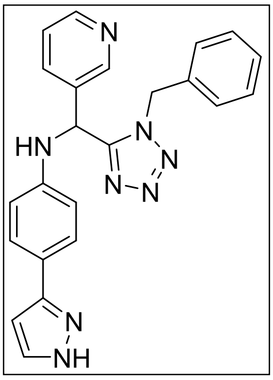

Synthesis according to procedure **A** afforded **1d** (451 mg, 1.10 mmol, 55 %) as a yellow solid; **mp:** 82 - 86 ˚C; **¹H NMR** (500 MHz, $CDCl_3$) δ 8.52 (dd, *J* = 4.8, 1.6 Hz, 1H), 8.46 (d, *J* = 2.4 Hz, 1H), 7.58 – 7.53 (m, 2H), 7.46 – 7.42 (m, 2H), 7.39 – 7.32 (m, 3H), 7.21 (dd, *J* = 8.0, 4.8 Hz, 1H), 7.14 – 7.08 (m, 2H), 6.44 (d, *J* = 2.3 Hz, 1H), 6.43 (d, *J* = 8.6 Hz, 2H), 5.78 (s, 1H), 5.63 (d, *J* = 15.5 Hz, 1H), 5.44 (d, *J* = 15.5 Hz, 1H), 5.07 (s, 1H) ppm. **¹³C NMR** (126 MHz, $CDCl_3$) δ 154.7, 150.2, 148.8, 144.9, 135.1, 133.5, 132.6, 132.6, 129.5, 129.4, 127.6, 127.0, 124.1, 123.5, 114.1, 101.9, 51.6, 50.9 ppm. **HRMS** (ESI) m/z calculated for $C_{23}H_{20}N_8$ $[M+H]^+$: 409.1889, found $[M+H]^+$: 409.1884.

N-((1-benzyl-1H-tetrazol-5-yl)(pyridin-3-yl)methyl)-4-(1,3,4-oxadiazol-2-yl)aniline (1e)

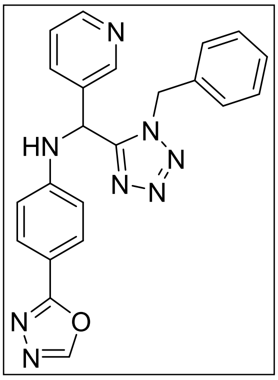

Synthesis according to procedure **A** afforded **1e** (429 mg, 1.04 mmol, 52 %) as an off-white solid; **mp:** 128 - 134 ˚C; **¹H NMR** (500 MHz, $CDCl_3$) δ 8.59 – 8.54 (m, 1H), 8.49 (d, *J* = 2.4 Hz, 1H), 8.35 (d, *J* = 0.8 Hz, 1H), 7.80 – 7.76 (m, 2H), 7.57 (dt, *J* = 8.1, 2.1 Hz, 1H), 7.41 – 7.35 (m, 3H), 7.29 – 7.23 (m, 1H), 7.15 – 7.10 (m, 2H), 6.47 – 6.43 (m, 2H), 5.77 (d, *J* = 7.7 Hz, 1H), 5.66 (d, *J* = 15.5 Hz, 1H), 5.40 (d, *J* = 15.6 Hz, 1H), 5.25 (d, *J* = 7.7 Hz, 1H) ppm. **¹³C NMR** (126 MHz, $CDCl_3$) δ 164.7, 154.3, 152.0, 150.6, 148.8, 147.8, 135.0, 132.4, 132.0, 129.6, 129.6, 128.8, 127.5, 124.3, 114.4, 113.5, 51.6, 50.5 ppm. **HRMS** (ESI) m/z calculated for $C_{22}H_{18}N_8O$ $[M+H]^+$: 411.1682, found $[M+H]^+$: 411.1676.

N-((1-benzyl-1H-tetrazol-5-yl)(pyridin-3-yl)methyl)-4-(tert-butyl)aniline (1f)

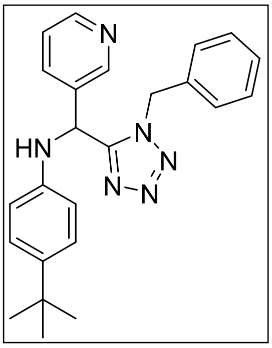

Synthesis according to procedure **A** afforded **1f** (340 mg, 0.86 mmol, 43 %) as an off-white solid; **mp:** 118 - 122˚C; **¹H NMR** (500 MHz, $CDCl_3$) δ 8.51 (dd, *J* = 4.8, 1.6 Hz, 1H), 8.41 (d, *J* = 2.4 Hz, 1H), 7.53 (dt, *J* = 8.0, 2.0 Hz, 1H), 7.40 – 7.31 (m, 3H), 7.20 (ddd, *J* = 8.0, 4.8, 0.9 Hz, 1H), 7.15 – 7.11 (m, 2H), 7.11 – 7.07 (m, 2H), 6.43 – 6.39 (m, 2H), 5.75 (d, *J* = 6.4 Hz, 1H), 5.59 (d, *J* = 15.5 Hz, 1H), 5.46 (d, *J* = 15.4 Hz, 1H), 4.56 (d, *J* = 7.6 Hz, 1H), 1.24 (s, 9H) ppm. **¹³C NMR** (126 MHz, $CDCl_3$) δ 154.9, 150.1, 148.8, 142.7, 142.4, 134.9, 133.0, 132.8, 129.4, 129.3, 127.6, 126.3, 124.0, 113.8, 51.5, 51.4, 34.0, 31.4 ppm. **HRMS** (ESI) m/z calculated for $C_{24}H_{26}N_6$ $[M+H]^+$: 399.2297, found $[M+H]^+$: 399.2292.

N-((1-benzyl-1H-tetrazol-5-yl)(pyridin-3-yl)methyl)-[1,1'-biphenyl]-4-amine (1g)

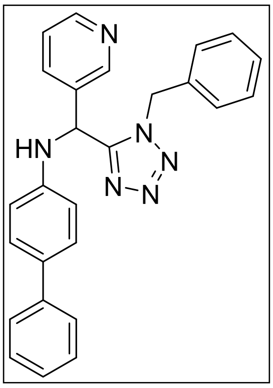

Synthesis according to procedure **A** afforded **1g** (1.50 g, 3.6 mmol, 72 %) as an white solid; **mp:** 164 - 170 ˚C; **^{1}H NMR** (500 MHz, $CDCl_3$) δ 8.53 (dd, *J* = 4.8, 1.6 Hz, 1H), 8.45 (d, *J* = 2.4 Hz, 1H), 7.56 (dt, *J* = 8.0, 1.9 Hz, 1H), 7.49 – 7.45 (m, 2H), 7.41 – 7.31 (m, 7H), 7.29 – 7.24 (m, 1H), 7.22 (dd, *J* = 8.0, 4.8 Hz, 1H), 7.15 – 7.10 (m, 2H), 6.49 (d, *J* = 8.4 Hz, 2H), 5.79 (d, *J* = 7.9 Hz, 1H), 5.63 (d, *J* = 15.5 Hz, 1H), 5.46 (d, *J* = 15.5 Hz, 1H), 4.84 (d, *J* = 7.9 Hz, 1H) ppm. **^{13}C NMR** (126 MHz, $CDCl_3$) δ 154.7, 150.3, 148.9, 144.2, 140.6, 134.9, 132.8, 132.7, 132.7, 129.5, 129.4, 128.7, 128.2, 127.6, 126.6, 126.5, 124.1, 114.2, 51.6, 51.2 ppm. **HRMS** (ESI) m/z calculated for $C_{26}H_{22}N_6$ $[M+H]^+$: 419.1984, found $[M+H]^+$: 419.1979.

N-((1-benzyl-1H-tetrazol-5-yl)(1H-pyrazol-3-yl)methyl)-4-(tert-butyl)aniline (1h)

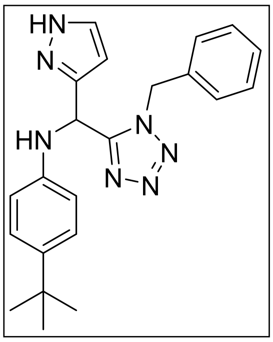

Synthesis according to procedure **A** afforded **1h** (315 mg, 0.81 mmol, 41 %) as a white solid; **mp:** 174 - 182 ˚C; **^{1}H NMR** (500 MHz, $CDCl_3$) δ 10.25 (s, 1H), 7.46 (d, *J* = 2.4 Hz, 1H), 7.25 – 7.19 (m, 3H), 7.15 – 7.08 (m, 2H), 7.03 (dt, *J* = 6.6, 1.6 Hz, 2H), 6.57 – 6.43 (m, 2H), 6.15 (d, *J* = 2.5 Hz, 1H), 6.12 (d, *J* = 5.3 Hz, 1H), 5.65 (d, *J* = 15.2 Hz, 1H), 5.55 (d, *J* = 15.2 Hz, 1H), 5.08 (d, *J* = 5.2 Hz, 1H), 1.24 (s, 9H) ppm. **^{13}C NMR** (126 MHz, $CDCl_3$) δ 155.1, 143.2, 142.0, 133.5, 128.8, 128.5, 127.8, 126.1, 113.5, 103.9, 51.3, 48.9, 33.9, 31.5 ppm. **HRMS** (ESI) m/z calculated for $C_{22}H_{25}N_7$ $[M+H]^+$: 388.2250, found $[M+H]^+$: 388.2244.

N-((1-benzyl-1H-tetrazol-5-yl)(imidazo[1,2-a]pyridin-3-yl)methyl)-4-(tert-butyl)aniline (1i)

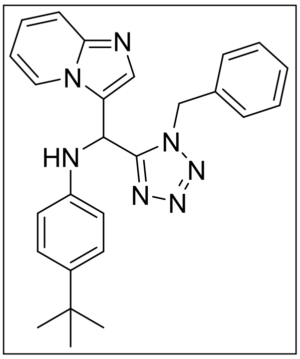

Synthesis according to procedure **A** afforded **1i** (216 mg, 0.49 mmol, 25 %) as a yellow solid; **mp:** 91 - 96 ˚C; **^{1}H NMR** (500 MHz, $CDCl_3$) δ 8.12 (dt, *J* = 6.9, 1.2 Hz, 1H), 7.62 (dt, *J* = 9.2, 1.1 Hz, 1H), 7.36 – 7.27 (m, 3H), 7.26 – 7.22 (m, 2H), 7.14 – 7.09 (m, 2H), 7.05 – 7.01 (m, 2H), 6.82 (td, *J* = 6.8, 1.2 Hz, 1H), 6.45 – 6.39 (m, 2H), 6.02 (d, *J* = 7.1 Hz, 1H), 5.70 (d, *J* = 15.4 Hz, 1H), 5.29 (d, *J* = 15.4 Hz, 1H), 4.63 (d, *J* = 7.1 Hz, 1H), 1.24 (s, 9H) ppm. **^{13}C NMR** (126 MHz, $CDCl_3$) δ 153.2, 147.0, 142.9, 142.6, 133.3, 132.5, 129.5, 129.4, 129.2, 127.5, 126.3, 125.4, 124.4, 118.5, 118.2, 114.0, 113.1, 51.7, 46.3, 34.0, 31.4 ppm. **HRMS** (ESI) m/z calculated for $C_{26}H_{27}N_7$ $[M+H]^+$: 438.2406, found $[M+H]^+$: 438.2401.

N-((1-benzyl-1H-tetrazol-5-yl)(pyridazin-3-yl)methyl)-4-(tert-butyl)aniline (1j)

Synthesis according to procedure **A** afforded **1j** (127 mg, 0.32 mmol, 32 %) as a yellow/orange solid; **mp:** 151 - 156 ˚C; **^{1}H NMR** (500 MHz, $CDCl_3$) δ 9.07 (dd, *J* = 4.9, 1.6 Hz, 1H), 7.50 (dd, *J* = 8.6, 1.6 Hz, 1H), 7.34 (dd, *J* = 8.6, 4.9 Hz, 1H), 7.18 (tt, *J* = 6.4, 4.1 Hz, 5H), 7.06 – 7.01 (m, 2H), 6.63 – 6.58 (m, 2H), 6.30 (d, *J* = 4.3 Hz, 1H), 5.92 (d, *J* = 4.3 Hz, 1H), 5.75 (d, *J* = 15.4 Hz, 1H), 5.72 (d, *J* = 15.4 Hz, 1H), 1.25 (s, 9H) ppm. **^{13}C NMR** (126 MHz, $CDCl_3$) δ 158.4, 154.1, 151.5, 142.5, 142.4, 133.1, 128.9, 128.6, 127.7, 127.5, 126.4, 125.6, 113.5,

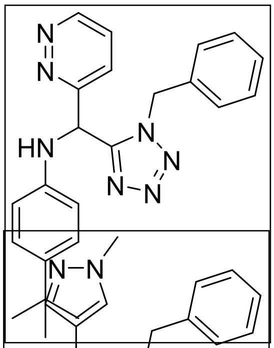

53.7, 51.5, 34.0, 31.4 ppm. **HRMS** (ESI) m/z calculated for $C_{23}H_{25}N_7$ $[M+H]^+$: 400.2250, found $[M+H]^+$: 400.2244.

N-((1-benzyl-1H-tetrazol-5-yl)(1-methyl-1H-pyrazol-4-yl)methyl)-4-(tert-butyl)aniline (1k)

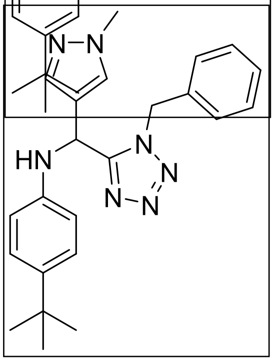

Synthesis according to procedure **A** afforded **1k** (546 mg, 1.36 mmol, 68 %) as a yellow solid; **mp:** 148 - 152 ˚C; **^{1}H NMR** (500 MHz, $CDCl_3$) δ 7.38 – 7.31 (m, 3H), 7.25 (d, *J* = 0.8 Hz, 1H), 7.13 – 7.10 (m, 4H), 7.00 (s, 1H), 6.40 – 6.37 (m, 2H), 5.83 (d, *J* = 6.8 Hz, 1H), 5.63 (d, *J* = 15.3 Hz, 1H), 5.44 (d, *J* = 15.3 Hz, 1H), 4.26 (d, *J* = 6.9 Hz, 1H), 3.75 (s, 3H), 1.24 (s, 9H) ppm. **^{13}C NMR** (126 MHz, $CDCl_3$) δ 155.7, 142.9, 142.4, 137.6, 133.4, 129.2, 129.0, 128.9, 127.8, 126.2, 118.7, 113.6, 51.5, 46.0, 39.1, 33.9, 31.4 ppm. **HRMS** (ESI) m/z calculated for $C_{23}H_{27}N_7$ $[M+H]^+$: 402.2406, found $[M+H]^+$: 402.2401.

N-((1-benzyl-1H-tetrazol-5-yl)(5-methylpyridin-3-yl)methyl)-4-(tert-butyl)aniline (1l)

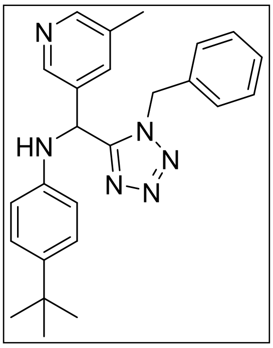

Synthesis according to procedure **A** afforded **1l** (552 mg, 1.34 mmol, 67 %) as an orange/brown oil. **^{1}H NMR** (500 MHz, $CDCl_3$) δ 8.34 (d, *J* = 2.1 Hz, 1H), 8.26 (d, *J* = 2.5 Hz, 1H), 7.39 – 7.32 (m, 3H), 7.25 (d, *J* = 2.4 Hz, 1H), 7.11 (ddd, *J* = 11.7, 7.2, 2.0 Hz, 4H), 6.42 – 6.38 (m, 2H), 5.72 (d, *J* = 7.3 Hz, 1H), 5.60 (d, *J* = 15.4 Hz, 1H), 5.43 (d, *J* = 15.4 Hz, 1H), 4.57 (d, *J* = 7.3 Hz, 1H), 2.22 (s, 3H), 1.24 (s, 9H) ppm. **^{13}C NMR** (126 MHz, $CDCl_3$) δ 155.0, 150.8, 145.9, 142.6, 135.3, 133.9, 132.9, 132.5, 129.4, 129.2, 129.2, 127.7, 127.6, 126.3, 113.6, 51.5, 51.4, 33.9, 31.4, 18.3 ppm. **HRMS** (ESI) m/z calculated for $C_{25}H_{28}N_6$ $[M+H]^+$: 413.2454, found $[M+H]^+$: 413.2448.

N-((1-benzyl-1H-tetrazol-5-yl)(1H-pyrazol-3-yl)methyl)-[1,1'-biphenyl]-4-amine (1m)

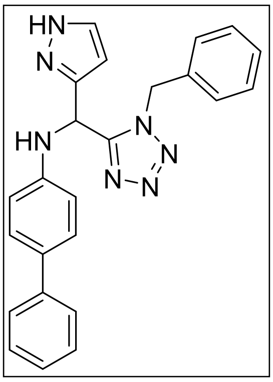

Synthesis according to procedure **A** afforded **1m** (511 mg, 1.25 mmol, 63 %) as a white solid; **mp:** 198 - 202 ˚C; **^{1}H NMR** (500 MHz, $CDCl_3$) δ 10.06 (s, 1H), 7.51 – 7.46 (m, 4H), 7.40 – 7.32 (m, 5H), 7.24 (d, *J* = 9.1 Hz, 2H), 7.09 – 7.05 (m, 2H), 6.64 – 6.59 (m, 2H), 6.21 (d, *J* = 2.5 Hz, 1H), 6.17 (d, *J* = 5.7 Hz, 1H), 5.69 (d, *J* = 15.2 Hz, 1H), 5.57 (d, *J* = 15.2 Hz, 1H), 5.26 (d, *J* = 5.6 Hz, 1H) ppm. **^{13}C NMR** (126 MHz, DMSO) δ 155.7, 148.3, 146.2, 140.3, 134.3, 129.8, 129.1, 128.7, 128.6, 128.3, 128.2, 127.1, 126.00, 125.6, 113.5, 103.5, 50.5, 47.0 ppm. **HRMS** (ESI) m/z calculated for $C_{24}H_{21}N_7$ $[M+H]^+$: 408.1937, found $[M+H]^+$: 408.1931.

N-((1-benzyl-1H-tetrazol-5-yl)(imidazo[1,2-a]pyridin-3-yl)methyl)-[1,1'-biphenyl]-4-amine (1n)

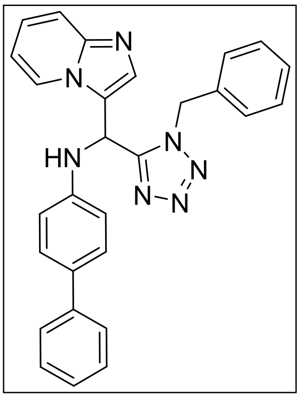

Synthesis according to procedure **A** afforded **1n** (82.9 mg, 0.18 mmol, 18 %) as a orange/brown oil. ^{1}**H NMR** (500 MHz, $CDCl_3$) δ 8.12 (d, *J* = 6.9, 1.1 Hz, 1H), 7.65 (d, *J* = 9.1, 1.1 Hz, 1H), 7.46 (dd, *J* = 8.2, 1.4 Hz, 2H), 7.41 – 7.27 (m, 10H), 7.06 (d, *J* = 7.0 Hz, 2H), 6.86 (qd, *J* = 6.8, 1.1 Hz, 1H), 6.52 – 6.48 (m, 2H), 6.06 (d, *J* = 7.1 Hz, 1H), 5.74 (d, *J* = 15.4 Hz, 1H), 5.28 (d, *J* = 15.4 Hz, 1H), 4.81 (d, *J* = 7.2 Hz, 1H) ppm. ^{13}**C NMR** (126 MHz, $CDCl_3$) δ 153.2, 147.2, 144.5, 140.7, 133.5, 133.2, 132.5, 129.6, 129.5, 128.9, 128.3, 127.7, 126.8, 126.6, 125.8, 124.4, 118.4, 114.5, 113.4, 51.9, 46.1 ppm. **HRMS** (ESI) m/z calculated for $C_{28}H_{23}N_7$ $[M+H]^+$: 458.2093, found $[M+H]^+$: 458.2088.

N-((1-benzyl-1H-tetrazol-5-yl)(pyridazin-3-yl)methyl)-[1,1'-biphenyl]-4-amine (1o)

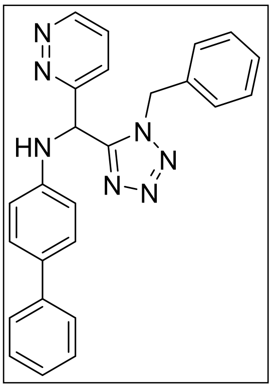

Synthesis according to procedure **A** afforded **1o** (383.7 mg, 0.91 mmol, 46 %) as an off-white solid; **mp:** 176 - 180 ˚C; ^{1}**H NMR** (500 MHz, $CDCl_3$) δ 9.09 (dd, *J* = 4.9, 1.6 Hz, 1H), 7.54 (dd, *J* = 8.6, 1.6 Hz, 1H), 7.51 – 7.47 (m, 2H), 7.41 – 7.35 (m, 5H), 7.29 – 7.26 (m, 1H), 7.23 – 7.17 (m, 3H), 7.09 – 7.04 (m, 2H), 6.75 – 6.69 (m, 2H), 6.37 (d, *J* = 4.6 Hz, 1H), 6.10 (d, *J* = 4.6 Hz, 1H), 5.78 (d, *J* = 15.3 Hz, 1H), 5.73 (d, *J* = 15.2 Hz, 1H) ppm. ^{13}**C NMR** (126 MHz, $CDCl_3$) δ 158.4, 154.0, 151.7, 144.4, 140.8, 133.1, 132.6, 129.1, 128.8, 128.8, 128.3, 127.8, 127.7, 126.7, 126.5, 125.8, 114.2, 53.5, 51.7 ppm. **HRMS** (ESI) m/z calculated for $C_{25}H_{21}N_7$ $[M+H]^+$: 420.1937, found $[M+H]^+$: 420.1931.

N-((1-(1-phenylcyclopropyl)-1H-tetrazol-5-yl)(pyridin-3-yl)methyl)-[1,1'-biphenyl]-4-amine (1p)

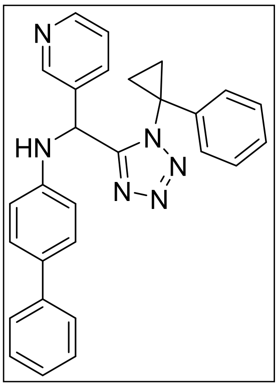

Synthesis according to procedure **A** afforded **1p** (102 mg, 0.229 mmol, 46 %) as an off-white solid; **mp:** 188 - 192 ˚C; ^{1}**H NMR** (500 MHz, $CDCl_3$) δ 8.51 (dd, *J* = 4.8, 1.6 Hz, 1H), 8.43 (d, *J* = 2.4 Hz, 1H), 7.62 (dt, *J* = 8.0, 2.0 Hz, 1H), 7.48 – 7.44 (m, 2H), 7.38 (t, *J* = 7.8 Hz, 2H), 7.35 – 7.23 (m, 6H), 7.21 (dd, *J* = 8.0, 4.8 Hz, 1H), 7.01 – 6.96 (m, 2H), 6.44 (d, *J* = 8.7 Hz, 2H), 5.89 (d, *J* = 8.7 Hz, 1H), 4.76 (d, *J* = 8.7 Hz, 1H), 1.89 (ddd, *J* = 11.0, 7.4, 5.9 Hz, 1H), 1.68 (dddd, *J* = 35.2, 10.3, 7.5, 6.0 Hz, 2H), 1.44 (ddd, *J* = 11.4, 7.4, 5.9 Hz, 1H) ppm.^{13}**C NMR** (126 MHz, $CDCl_3$) δ 156.6, 150.2, 149.4, 144.4, 140.8, 137.4, 135.2, 132.9, 132.6, 129.5, 128.8, 128.6, 128.2, 126.6, 126.5, 125.5, 124.1, 114.4, 51.0, 41.7, 16.2, 16.1 ppm. **HRMS** (ESI) m/z calculated for $C_{28}H_{24}N_6$ $[M+H]^+$: 445.2141, found $[M+H]^+$: 445.2135.

N-((1-((1-methylpiperidin-4-yl)methyl)-1H-tetrazol-5-yl)(pyridin-3-yl)methyl)-[1,1'-biphenyl]-4-amine (1q)

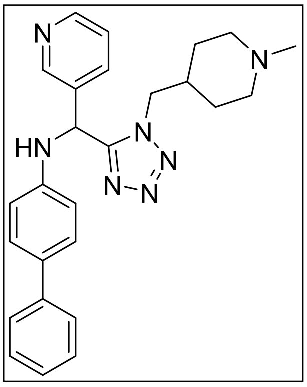

Synthesis according to procedure **A** afforded **1q** (82.6 mg, 0.188 mmol, 38 %) as an orange oil; **^{1}H NMR** (500 MHz, $CDCl_3$) δ 8.73 (d, *J* = 2.4 Hz, 1H), 8.61 (dt, *J* = 4.9, 1.3 Hz, 1H), 7.79 (dt, *J* = 8.1, 1.7 Hz, 1H), 7.49 (dt, *J* = 8.2, 1.3 Hz, 2H), 7.44 – 7.36 (m, 4H), 7.34 (dd, *J* = 8.0, 4.7 Hz, 1H), 7.31 – 7.27 (m, 1H), 6.75 (dd, *J* = 7.6, 1.6 Hz, 2H), 5.95 (d, *J* = 7.3 Hz, 1H), 5.12 (d, *J* = 7.3 Hz, 1H), 4.27 – 4.14 (m, 2H), 2.82 (dq, *J* = 9.6, 4.7, 4.1 Hz, 2H), 2.24 (s 3H), 2.09 (s, 2H), 1.82 (m, 3H), 1.65 – 1.46 (m, 1H), 1.44 – 1.22 (m, 1H) ppm. **^{13}C NMR** (126 MHz, $CDCl_3$) δ 155.0, 150.6, 149.0, 144.4, 140.7, 135.2, 133.4, 132.9, 128.9, 128.4, 126.8, 126.6, 126.3, 124.4, 114.5, 54.8, 53.1, 51.5, 46.0, 35.6, 29.5 ppm. **HRMS** (ESI) m/z calculated for $C_{26}H_{29}N_7$ $[M+H]^+$: 440.2563, found $[M+H]^+$: 440.2557.

4-(5-(([1,1'-biphenyl]-4-ylamino)(pyridin-3-yl)methyl)-1H-tetrazol-1-yl)cyclohexan-1-ol (1r)

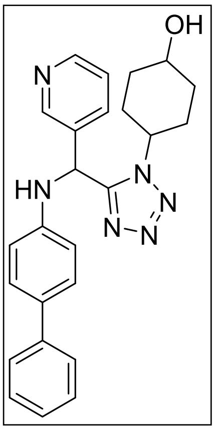

Synthesis according to procedure **A** afforded **1r** (150 mg, 0.353 mmol, 71 %) as a white solid; **mp:** 98 - 102 ˚C; Conformational isomer ratio 6:4. **^{1}H NMR** (500 MHz, DMSO-*d6*) (major) δ 8.78 (d, *J* = 2.6 Hz, 1H), 8.53 (d, *J* = 4.3 Hz, 1H), 8.27 (d, *J* = 2.6 Hz, 1H), 8.20 (d, *J* = 2.6 Hz, 0H), 7.94 (d, *J* = 7.8 Hz, 1H), 7.56 – 7.51 (m, 2H), 7.47 – 7.42 (m, 3H), 7.37 (td, *J* = 7.8, 2.5 Hz, 2H), 7.23 (t, *J* = 7.5 Hz, 1H), 7.09 (d, *J* = 7.8 Hz, 1H), 6.91 – 6.84 (m, 2H), 6.55 (dd, *J* = 9.4, 2.6 Hz, 1H), 4.91 – 4.68 (m, 2H), 2.19 – 1.88 (m, 4H), 1.83 (d, *J* = 13.1 Hz, 1H), 1.77 – 1.59 (m, 2H), 1.59 – 1.38 (m, 1H) ppm. **^{13}C NMR** (126 MHz, DMSO-*d6*) (major) δ 161.7, 154.8, 149.2, 145.9, 140.2, 135.5, 133.7, 129.5, 128.8, 127.3, 126.1, 125.7, 123.7, 113.9, 70.6, 67.2, 55.6, 48.5, 29.7, 28.2, 26.8 ppm. **HRMS** (ESI) m/z calculated for $C_{25}H_{26}N_6O$ $[M+H]^+$: 427.2246, found $[M+H]^+$: 427.2241.

4-(tert-butyl)-N-((1-(3-fluorobenzyl)-1H-tetrazol-5-yl)(pyridin-3-yl)methyl)aniline (1s)

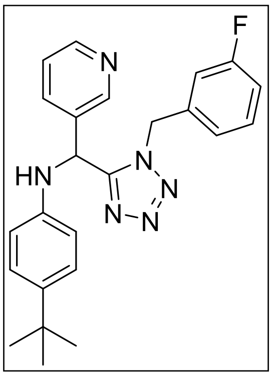

Synthesis according to procedure **A** afforded **1s** (420 mg, 0.992 mmol, 50 %) as a yellow solid; **mp:** 94 - 98 ˚C; **^{1}H NMR** (500 MHz, DMSO) δ 8.68 (d, *J* = 2.3 Hz, 1H), 8.48 (dd, *J* = 4.8, 1.7 Hz, 1H), 7.86 (dt, *J* = 8.0, 2.0 Hz, 1H), 7.35 (ddd, *J* = 15.5, 8.0, 5.6 Hz, 2H), 7.13 (td, *J* = 8.7, 2.6 Hz, 1H), 7.09 (d, *J* = 8.6 Hz, 2H), 7.03 (d, *J* = 7.7 Hz, 1H), 6.99 (dt, *J* = 10.0, 2.1 Hz, 1H), 6.72 (d, *J* = 9.3 Hz, 1H), 6.68 – 6.62 (m, 2H), 6.40 (d, *J* = 9.3 Hz, 1H), 5.80 (s, 2H), 1.18 (s, 9H) ppm. **^{13}C NMR** (126 MHz, DMSO) δ 163.0, 161.0, 155.7, 149.2, 149.1, 143.6, 139.9, 136.83, 136.77, 135.5, 133.8, 130.7, 130.6, 125.5, 124.1, 124.1, 123.5, 115.2, 115.04, 115.01, 114.9, 113.2, 49.7, 48.8, 33.5, 31.3 ppm. **HRMS** (ESI) m/z calculated for $C_{24}H_{25}FN_6$ $[M+H]^+$: 417.2203, found $[M+H]^+$: 417.2197.

4-(tert-butyl)-N-((1-(3-fluorophenethyl)-1H-tetrazol-5-yl)(pyridin-3-yl)methyl)aniline (1t)

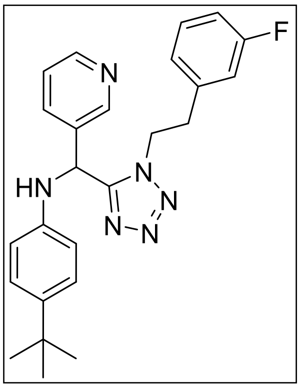

Synthesis according to procedure **A** afforded **1t** (392 mg, 0.910 mmol, 46 %) as an off-white solid; **mp:** 122 - 126 ˚C; **^{1}H NMR** (500 MHz, DMSO) δ 8.67 (d, *J* = 2.3 Hz, 1H), 8.52 (dd, *J* = 4.8, 1.6 Hz, 1H), 7.86 (dt, *J* = 8.0, 2.0 Hz, 1H), 7.41 (dd, *J* = 7.9, 4.7 Hz, 1H), 7.27 (td, *J* = 7.9, 6.1 Hz, 1H), 7.17 – 7.10 (m, 2H), 7.04 (td, *J* = 8.7, 2.7 Hz, 1H), 6.99 – 6.87 (m, 2H), 6.79 (d, *J* = 9.4 Hz, 1H), 6.74 – 6.62 (m, 2H), 6.21 (d, *J* = 9.4 Hz, 1H), 4.82 – 4.66 (m, 2H), 3.25 – 3.04 (m, 2H), 1.19 (s, 9H) ppm. **^{13}C NMR** (126 MHz, DMSO) δ 163.1, 161.2, 155.5, 149.1, 149.0, 143.7, 139.93, 139.91, 139.85, 135.5, 134.0, 130.4, 130.3, 125.7, 125.01, 124.99, 123.6, 115.7, 115.6, 113.7, 113.6, 112.9, 48.5, 48.0, 34.5, 33.5, 31.3 ppm. **HRMS** (ESI) m/z calculated for $C_{25}H_{27}FN_6$ $[M+H]^+$: 431.2360, found $[M+H]^+$: 431.2354.

2-methyl-N-((1-(1-phenylcyclopropyl)-1H-tetrazol-5-yl)(pyridin-3-yl)methyl)-[1,1'-biphenyl]-4-amine (1u)

Synthesis according to procedure **A** afforded **1u** (150 mg, 0.327 mmol, 65 %) as a yellow solid; **mp:** 143 - 148 ˚C; **^{1}H NMR** (500 MHz, $CDCl_3$) δ 8.51 (dd, *J* = 4.8, 1.6 Hz, 1H), 8.42 (d, *J* = 2.4 Hz, 1H), 7.60 (dt, *J* = 8.0, 2.1 Hz, 1H), 7.36 (dd, *J* = 8.2, 6.9 Hz, 2H), 7.32 – 7.27 (m, 4H), 7.24 – 7.19 (m, 3H), 7.01 – 6.95 (m, 2H), 6.91 (d, *J* = 8.2 Hz, 1H), 6.35 (d, *J* = 2.5 Hz, 1H), 6.20 (dd, *J* = 8.2, 2.6 Hz, 1H), 5.88 (d, *J* = 8.9 Hz, 1H), 4.66 (d, *J* = 8.9 Hz, 1H), 2.11 (s, 3H), 1.88 (ddd, *J* = 11.1, 7.5, 6.0 Hz, 1H), 1.71 (ddd, *J* = 10.3, 7.5, 5.9 Hz, 1H), 1.62 (ddd, *J* = 10.2, 7.6, 5.9 Hz, 1H), 1.43 (ddd, *J* = 11.2, 7.6, 6.0 Hz, 1H) ppm. **^{13}C NMR** (126 MHz, $CDCl_3$) δ 156.7, 150.1, 149.3, 144.2, 141.7, 137.4, 136.7, 135.2, 133.7, 130.9, 129.5, 128.6, 128.1, 126.5, 125.5, 116.1, 111.2, 51.1, 41.7, 20.8, 16.2, 16.1 ppm. **HRMS** (ESI) m/z calculated for $C_{29}H_{26}N_6$ $[M+H]^+$: 459.2297, found $[M+H]^+$: 459.2292.

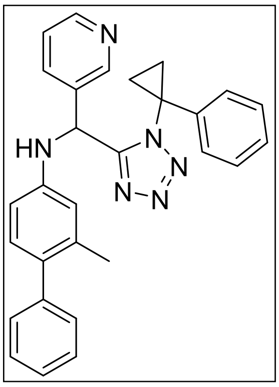

2-chloro-N-((1-(1-phenylcyclopropyl)-1H-tetrazol-5-yl)(pyridin-3-yl)methyl)-[1,1'-biphenyl]-4-amine (1v)

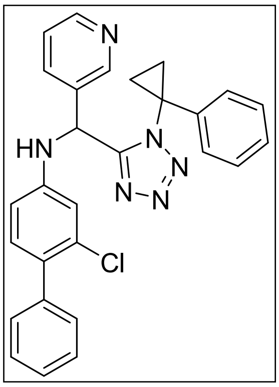

Synthesis according to procedure **A** afforded **1v** (69 mg, 0.144 mmol, 29 %) as a off-white solid; **mp:** 164 - 168 ˚C; **^{1}H NMR** (500 MHz, $CDCl_3$) δ 8.53 (dd, *J* = 4.9, 1.6 Hz, 1H), 8.43 (d, *J* = 2.4 Hz, 1H), 7.58 (dt, *J* = 7.9, 2.1 Hz, 1H), 7.40 – 7.36 (m, 2H), 7.36 – 7.29 (m, 6H), 7.23 (dd, *J* = 8.0, 4.7 Hz, 1H), 7.01 (d, *J* = 8.3 Hz, 1H), 6.99 – 6.97 (m, 1H), 6.96 (d, *J* = 2.2 Hz, 1H), 6.51 (d, *J* = 2.4 Hz, 1H), 6.29 (dd, *J* = 8.4, 2.5 Hz, 1H), 5.82 (d, *J* = 8.6 Hz, 1H), 4.84 (d, *J* = 8.6 Hz, 1H), 1.92 (ddd, *J* = 11.0, 7.5, 5.9 Hz, 1H), 1.72 (dt, *J* = 10.3, 7.5, 5.9 Hz, 1H), 1.63 (dt, *J* = 10.3, 7.5, 5.9 Hz, 1H), 1.42 (ddd, *J* = 11.4, 7.5, 6.0 Hz, 1H) ppm. **^{13}C NMR** (126 MHz, $CDCl_3$) δ 156.3, 150.4, 149.3, 145.2, 139.2, 137.2, 135.2, 133.3, 132.5, 132.2, 131.7, 129.6, 129.6, 128.8, 128.1, 127.3, 125.4, 124.2, 114.8, 112.6, 50.9, 41.8, 16.1 ppm. **HRMS** (ESI) m/z calculated for $C_{28}H_{23}N_6Cl$ $[M+H]^+$: 479.1751, found $[M+H]^+$: 479.1745.

N-((1-(1-phenylcyclopropyl)-1H-tetrazol-5-yl)(pyridin-3-yl)methyl)-2-(trifluoromethyl)-[1,1'-biphenyl]-4-amine (1w)

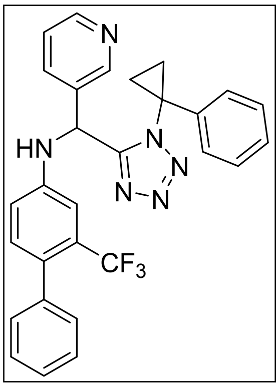

Synthesis according to procedure **A** afforded **1w** (131 mg, 0.256 mmol, 51 %) as a white sticky oil. **^{1}H NMR** (500 MHz, $CDCl_3$) δ 8.54 (d, *J* = 4.8 Hz, 1H), 8.45 (d, *J* = 2.3 Hz, 1H), 7.58 (dt, *J* = 8.0, 2.1 Hz, 1H), 7.34 (qd, *J* = 4.0, 2.2 Hz, 3H), 7.31 – 7.27 (m, *J* = 2.2 Hz, 3H), 7.23 (dt, *J* = 6.1, 3.2 Hz, 3H), 7.00 – 6.93 (m, 3H), 6.81 (d, *J* = 2.6 Hz, 1H), 6.40 (dd, *J* = 8.4, 2.6 Hz, 1H), 5.85 (d, *J* = 8.5 Hz, 1H), 4.97 (d, *J* = 8.5 Hz, 1H), 1.95 – 1.88 (m, 1H), 1.71 (ddd, *J* = 10.4, 7.6, 6.1 Hz, 1H), 1.67 – 1.59 (m, 1H), 1.46 – 1.39 (m, 1H) ppm. **^{13}C NMR** (126 MHz, $CDCl_3$) δ 156.2, 150.4, 149.3, 144.1, 139.7, 137.1, 135.2, 133.3, 132.3, 129.5, 129.4, 129.4, 128.8, 127.8, 127.4, 125.5, 125.0, 124.2, 122.9, 115.7, 111.6, 111.6, 60.6, 51.0, 41.9, 21.2, 16.1, 16.1, 14.3 ppm. **HRMS** (ESI) m/z calculated for $C_{29}H_{23}F_3N_6$ $[M+H]^+$: 513.2015, found $[M+H]^+$: 513.2009.

2'-methyl-N-((1-(1-phenylcyclopropyl)-1H-tetrazol-5-yl)(pyridin-3-yl)methyl)-[1,1'-biphenyl]-4-amine (1x)

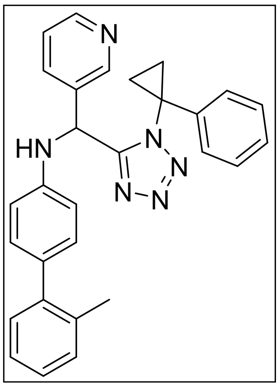

Synthesis according to procedure **A** afforded **1x** (123 mg, 0.268 mmol, 54 %) as a pale yellow solid; **mp:** 210 - 216 ˚C; **^{1}H NMR** (500 MHz, $CDCl_3$) δ 8.52 (d, *J* = 4.9 Hz, 1H), 8.45 (s, 1H), 7.62 (dt, *J* = 7.9, 2.0 Hz, 1H), 7.32 – 7.28 (m, 3H), 7.24 – 7.17 (m, 4H), 7.14 – 7.11 (m, 1H), 7.03 – 6.96 (m, 4H), 6.41 – 6.36 (m, 2H), 5.89 (d, *J* = 8.6 Hz, 1H), 4.73 (d, *J* = 8.7 Hz, 1H), 2.22 (s, 3H), 1.90 (ddd, *J* = 10.9, 7.2, 5.6 Hz, 1H), 1.75 – 1.61 (m, 2H), 1.42 (ddd, *J* = 11.7, 7.3, 5.7 Hz, 1H) ppm. **^{13}C NMR** (126 MHz, $CDCl_3$) δ 156.7, 150.2, 149.4, 143.8, 141.6, 137.3, 135.5, 135.3, 133.3, 130.4, 130.3, 129.9, 129.5, 128.7, 127.0, 125.9, 125.6, 124.1, 113.6, 51.1, 41.7, 20.7, 16.1, 16.0 ppm. **HRMS** (ESI) m/z calculated for $C_{29}H_{26}N_6$ $[M+H]^+$: 459.2297, found $[M+H]^+$: 495.1695.

2'-methyl-N-((1-(1-phenylcyclopropyl)-1H-tetrazol-5-yl)(pyridin-3-yl)methyl)-[1,1'-biphenyl]-4-amine (1y)

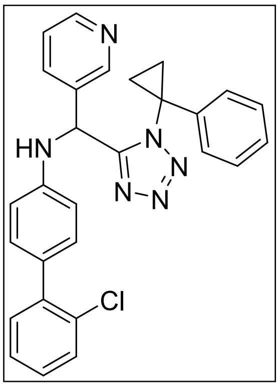

Synthesis according to procedure **A** afforded **1y** (449 mg, 0.937 mmol, 47 %) as a yellow oil. **^{1}H NMR** (500 MHz, DMSO) δ 8.60 (d, *J* = 2.4 Hz, 1H), 8.47 (d, *J* = 4.7 Hz, 1H), 7.81 (d, *J* = 8.0 Hz, 1H), 7.47 (d, *J* = 7.8 Hz, 1H), 7.30 (dq, *J* = 19.7, 7.2 Hz, 7H), 7.08 (d, *J* = 8.1 Hz, 2H), 6.99 (d, *J* = 7.0 Hz, 2H), 6.94 (d, *J* = 8.3 Hz, 1H), 6.55 (d, *J* = 8.1 Hz, 2H), 6.08 (d, *J* = 8.2 Hz, 1H), 1.96 – 1.86 (m, 1H), 1.82 – 1.62 (m, 3H) ppm. **^{13}C NMR** (126 MHz, DMSO) δ 156.7, 149.30, 149.26, 145.7, 139.8, 138.1, 135.6, 133.2, 131.31, 131.26, 129.84, 129.79, 128.8, 128.2, 127.7, 127.4, 125.3, 123.6, 112.6, 49.3, 41.3, 16.0, 15.8 ppm. **HRMS** (ESI) m/z calculated for $C_{28}H_{23}ClN_6$ $[M+H]^+$: 479.1751, found $[M+H]^+$: 479.1745.

N-((1-(1-phenylcyclopropyl)-1H-tetrazol-5-yl)(pyridin-3-yl)methyl)-2'-(trifluoromethyl)-[1,1'-biphenyl]-4-amine (1z)

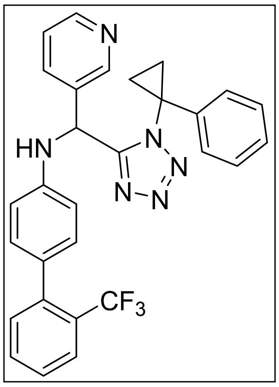

Synthesis according to procedure **A** afforded **1z** (84 mg, 0.165 mmol, 33 %) as a white solid; **mp:** 178 - 182 °C; **^{1}H NMR** (500 MHz, $CDCl_3$) δ 8.52 (d, *J* = 4.8 Hz, 1H), 8.45 (s, 1H), 7.70 (d, *J* = 7.9 Hz, 1H), 7.63 (dt, *J* = 7.9, 2.0 Hz, 1H), 7.51 (t, *J* = 7.5 Hz, 1H), 7.41 (t, *J* = 7.6 Hz, 1H), 7.33 – 7.27 (m, 3H), 7.25 – 7.21 (m, 2H), 7.02 (s, 1H), 7.01 – 6.96 (m, 3H), 6.36 (d, *J* = 8.6 Hz, 2H), 5.88 (d, *J* = 8.4 Hz, 1H), 4.76 (d, *J* = 8.4 Hz, 1H), 1.89 (ddd, *J* = 10.5, 7.2, 5.6 Hz, 1H), 1.70 (ddd, *J* = 10.1, 7.2, 5.6 Hz, 1H), 1.64 (ddd, *J* = 10.5, 7.2, 5.3 Hz, 1H), 1.44 (ddd, *J* = 11.5, 7.1, 5.6 Hz, 1H) ppm. **^{13}C NMR** (126 MHz, $CDCl_3$) δ 156.6, 150.2, 149.4, 144.6, 141.3, 137.3, 135.3, 132.4, 131.4, 131.1, 130.1, 129.5, 128.7, 127.1, 126.2, 126.2, 125.6, 124.1, 113.2, 51.1, 41.8, 16.1, 16.0 ppm. **HRMS** (ESI) m/z calculated for $C_{29}H_{23}F_3N_6$ $[M+H]^+$: 513.2015, found $[M+H]^+$: 513.2009.

3-(([1,1'-biphenyl]-4-ylamino)(1-(3-methoxyphenyl)-1H-tetrazol-5-yl)methyl)phenol (1aa)

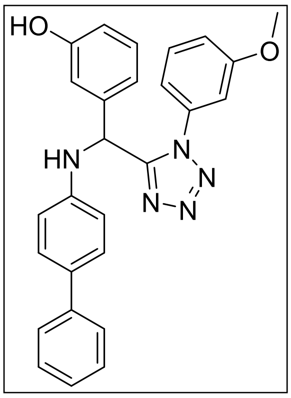

Synthesis according to procedure **A** afforded **1aa** (349 mg, 0.777 mmol, 78 %) as an off-white solid; **mp:** 82 - 86 °C; **^{1}H NMR** (500 MHz, $CDCl_3$) δ 7.50 – 7.43 (m, 3H), 7.40 (d, *J* = 12.3 Hz, 1H), 7.38 – 7.33 (m, 3H), 7.30 – 7.21 (m, 1H), 7.19 (t, *J* = 7.8 Hz, 1H), 7.13 (dd, *J* = 8.5, 2.5 Hz, 1H), 6.90 (dd, *J* = 7.7, 2.0 Hz, 1H), 6.82 (ddt, *J* = 10.9, 5.2, 2.3 Hz, 3H), 6.76 (t, *J* = 2.2 Hz, 1H), 6.56 (d, *J* = 8.5 Hz, 2H), 6.11 (s, 1H), 5.89 (d, *J* = 8.2 Hz, 1H), 4.90 (d, *J* = 8.4 Hz, 1H), 3.71 (s, 3H) ppm. **^{13}C NMR** (126 MHz, $CDCl_3$) δ 160.6, 156.8, 156.0, 144.7, 140.8, 138.9, 134.2, 132.3, 130.8, 130.6, 128.8, 128.1, 126.5, 126.4, 119.6, 117.7, 117.5, 116.3, 114.3, 114.2, 110.7, 55.7, 52.9 ppm. **HRMS** (ESI) m/z calculated for $C_{27}H_{23}N_5O_2$ $[M+H]^+$: 450.1930, found $[M+H]^+$: 450.1927.

(3-(([1,1'-biphenyl]-4-ylamino)(1-(3-methoxyphenyl)-1H-tetrazol-5-yl)methyl)phenyl)boronic acid (1ab)

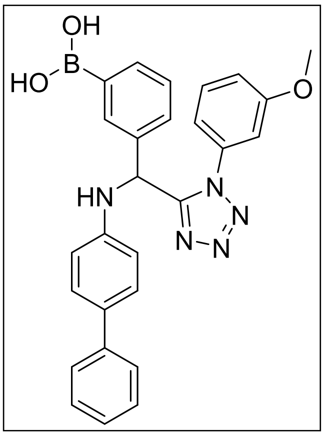

Synthesis according to procedure **A** afforded **1ab** as a mixture of atropisomers (1:0.6) (189 mg, 0.396 mmol, 40 %) as an off-white solid; **mp:** 138 - 142 °C; **^{1}H NMR** (500 MHz, $CDCl_3$) δ 8.18 – 8.07 (m, 2H), 7.84 (s, 0.5H), 7.78 – 7.69 (m, 0.5H), 7.56 (d, *J* = 7.8 Hz, 1H), 7.53 – 7.29 (m, 13H), 7.23 (d, *J* = 7.3 Hz, 1H), 7.14 – 7.02 (m, 2H), 6.95 (d, *J* = 7.8 Hz, 1H), 6.89 (d, *J* = 8.0 Hz, 1H), 6.83 – 6.74 (m, 2H), 6.72 – 6.66 (m, 2H), 6.58 (dd, *J* = 8.8, 7.2 Hz, 1H), 6.07 (s, 1H), 5.93 (s,0.5H), 3.66 (s, 2H), 3.62 (s, 3H) ppm. **^{13}C NMR** (126 MHz, $CDCl_3$) δ 160.6, 155.9, 144.91, 144.87, 144.8, 140.8, 137.3, 136.2, 134.7, 134.4, 132.3, 132.2, 132.0, 130.8, 130.7, 129.1, 128.78, 128.76, 128.13, 128.08, 126.5, 126.4, 117., 117.53, 117.49, 114.3, 114.2, 110.9, 110.8, 110.7, 55.6, 53.2, 53.1 ppm. **HRMS** (ESI) m/z calculated for $C_{27}H_{24}BN_5O_3$ $[M+H]^+$: 478.2050, found $[M+H]^+$: 478.2053.

N-([1,1'-biphenyl]-4-yl)-N-((1-benzyl-1H-tetrazol-5-yl)(pyridin-3-yl)methyl)-2-chloroacetamide (2a)

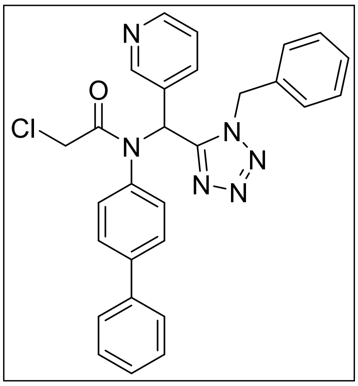

Synthesis according to procedure **B** afforded **2a** (25.8 mg, 0.052 mmol, 44 %) as a yellow solid; **mp:** 76 - 82 ˚C; **^{1}H NMR** (500 MHz, DMSO) δ 8.63 (d, *J* = 2.2 Hz, 1H), 8.60 – 8.56 (m, 1H), 7.84 (d, *J* = 8.1 Hz, 1H), 7.67 – 7.57 (m, 5H), 7.55 (s, 1H), 7.49 (t, *J* = 6.7 Hz, 1H), 7.45 (t, *J* = 7.6 Hz, 2H), 7.37 (t, *J* = 7.3 Hz, 1H), 7.28 (dd, *J* = 5.1, 1.9 Hz, 3H), 7.21 (dd, *J* = 6.6, 2.9 Hz, 3H), 5.87 (d, *J* = 15.5 Hz, 1H), 5.79 (d, *J* = 15.5 Hz, 1H), 4.10 (d, *J* = 14.4 Hz, 1H), 3.98 (d, *J* = 14.4 Hz, 1H) ppm. **^{13}C NMR** (126 MHz, DMSO) δ 166.3, 152.8, 145.1, 144.2, 144.0, 140.8, 138.3, 135.4, 133.7, 131.9, 131.0, 129.0, 128.8, 128.5, 128.2, 127.6, 127.4, 126.8, 126.7, 125.8, 125.7, 51.2, 51.1, 50.6, 43.1 ppm. **HRMS** (ESI) m/z calculated for $C_{28}H_{23}ClN_6O$ [M+H]$^+$: 495.1700, found [M+H]$^+$: 495.1695.

N-(4-(tert-butyl)phenyl)-2-chloro-N-((1-(3-fluorobenzyl)-1H-tetrazol-5-yl)(pyridin-3-yl)methyl)acetamide (2b)

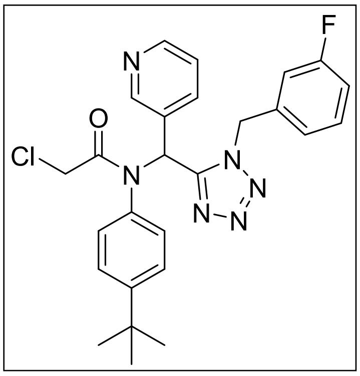

Synthesis according to procedure **B** afforded **2b** (24.2 mg, 0.049 mmol, 51 %) as a white solid; **mp:** 105 - 109 ˚C; **^{1}H NMR** (500 MHz, DMSO) δ 8.52 (d, *J* = 4.3 Hz, 2H), 7.67 (d, *J* = 8.0 Hz, 1H), 7.45 (s, 1H), 7.43 – 7.20 (m, 5H), 7.15 – 6.98 (m, 4H), 5.87 (d, *J* = 15.7 Hz, 1H), 5.78 (d, *J* = 15.4 Hz, 1H), 4.02 (d, *J* = 14.4 Hz, 1H), 3.93 (d, *J* = 14.3 Hz, 1H), 1.19 (s, 9H) ppm. **^{13}C NMR** (126 MHz, DMSO) δ 166.8, 163.4, 161.5, 153.6, 152.6, 148.6, 146.6, 145.4, 136.8, 136.8, 134.1, 131.8, 131.3, 130.5, 126.7, 125.6, 124.7, 115.9, 115.7, 115.6, 115.5, 51.7, 50.3, 43.3, 34.9, 31.4, 31.3 ppm. **HRMS** (ESI) m/z calculated for $C_{26}H_{26}ClFN_6O$ [M+H]$^+$: 493.1919, found [M+H]$^+$: 493.1914.

2-chloro-N-(2'-methyl-[1,1'-biphenyl]-4-yl)-N-((1-(1-phenylcyclopropyl)-1H-tetrazol-5-yl)(pyridin-3-yl)methyl)acetamide (2c)

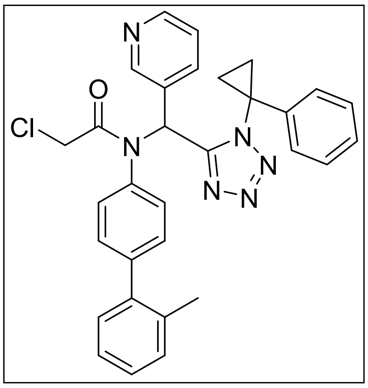

Synthesis according to procedure **B** afforded **2c** (21.4 mg, 0.040 mmol, 56 %) as an off-white solid; **mp:** 130 - 135 ˚C; **^{1}H NMR** (500 MHz, MeOD) δ 8.60 (d, *J* = 5.7 Hz, 1H), 8.57 (d, *J* = 2.0 Hz, 1H), 8.22 (d, *J* = 8.3 Hz, 1H), 7.91 (d, *J* = 0.9 Hz, 1H), 7.71 (dd, *J* = 8.2, 5.7 Hz, 1H), 7.50 (s, 1H), 7.32 – 7.16 (m, 6H), 7.16 – 7.03 (m, 4H), 6.97 – 6.91 (m, 2H), 4.03 (d, *J* = 14.3 Hz, 1H), 3.92 (d, *J* = 14.4 Hz, 1H), 2.31 – 2.22 (m, 1H), 2.20 – 2.14 (m, 2H), 2.10 (s, 3H), 1.64 (td, *J* = 8.7, 5.6 Hz, 1H) ppm. **^{13}C NMR** (126 MHz, MeOD) δ 168.9, 155.5, 148.6, 145.4, 144.6, 143.6, 141.3, 138.6, 136.0, 136.0, 134.6, 131.8, 131.4, 130.3, 130.0, 129.3, 129.0, 128.2, 127.1, 127.0, 53.5, 43.3, 42.9, 20.4, 17.6, 14.9 ppm. **HRMS** (ESI) m/z calculated for $C_{31}H_{27}ClN_6O$ [M+H]$^+$: 535.2013, found [M+H]$^+$: 535.2008.

2-chloro-N-((1-(1-(2-fluorophenyl)cyclopropyl)-1H-tetrazol-5-yl)(pyridin-3-yl)methyl)-N-(2'-methyl-[1,1'-biphenyl]-4-yl)acetamide (2d)

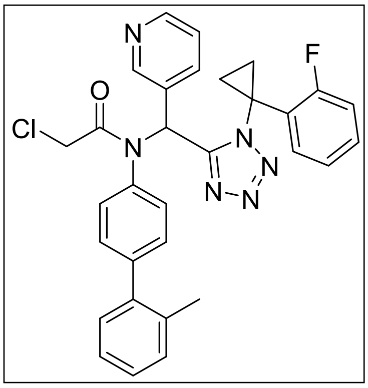

Synthesis according to procedure **B** afforded **2d** (22.7 mg, 0.041 mmol, 39 %) as a pale yellow solid; **mp:** 80 - 85 ˚C; **^{1}H NMR** (500 MHz, MeOD) δ 8.67 (s, 1H), 8.65 (d, *J* = 5.8 Hz, 1H), 8.25 (d, *J* = 8.2 Hz, 1H), 7.76 (dd, *J* = 8.1, 5.9 Hz, 1H), 7.61 – 7.48 (m, 3H), 7.28 – 7.16 (m, 7H), 7.12 – 7.04 (m, 2H), 6.77 (dd, *J* = 11.1, 8.3 Hz, 1H), 4.08 (dd, *J* = 14.4, 1.4 Hz, 1H), 3.96 (dd, *J* = 14.3, 1.3 Hz, 1H), 2.33 (dt, *J* = 9.6, 4.5 Hz, 1H), 2.19 – 2.09 (m, 2H), 2.08 (s, 3H), 1.69 – 1.58 (m, 1H) ppm. **^{13}C NMR** (126 MHz, MeOD) δ 168.9, 163.6, 161.6, 155.1, 148.5, 145.2, 144.7, 143.6, 141.3, 136.1, 136.0, 134.6, 132.6, 131.8, 131.4, 130.2, 129.0, 128.1, 127.0, 125.9, 125.3, 125.2, 117.2, 117.1, 53.5, 53.4, 43.2, 39.5, 20.4, 15.9, 14.2 ppm. **HRMS** (ESI) m/z calculated for $C_{31}H_{26}ClFN_6O$ [M+H]$^+$: 553.1919, found [M+H]$^+$: 553.1913.

N-([1,1'-biphenyl]-4-yl)-2-cyano-N-((1-(1-phenylcyclopropyl)-1H-tetrazol-5-yl)(pyridin-3-yl)methyl)acetamide (3a)

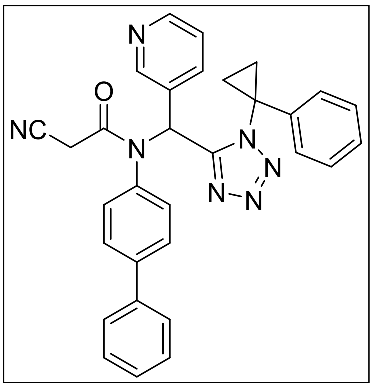

Synthesis according to procedure **C** afforded **3a** (42.6 mg, 0.083 mmol, 74 %) as an off-white solid; **mp:** 170 - 174 ˚C; **^{1}H NMR** (500 MHz, $CDCl_3$) δ 8.35 (dt, *J* = 4.8, 1.2 Hz, 1H), 8.14 (d, *J* = 2.3 Hz, 1H), 7.80 (d, *J* = 8.2 Hz, 1H), 7.69 – 7.62 (m, 1H), 7.51 – 7.46 (m, 2H), 7.43 (t, *J* = 7.5 Hz, 2H), 7.40 – 7.34 (m, 1H), 7.34 – 7.27 (m, 1H), 7.27 – 7.16 (m, 4H), 7.14 – 7.06 (m, 2H), 6.95 (s, 1H), 6.88 (dd, *J* = 8.1, 4.8 Hz, 1H), 6.46 (d, *J* = 8.3 Hz, 1H), 5.82 (d, *J* = 15.6 Hz, 1H), 5.76 (d, *J* = 15.5 Hz, 1H), 3.24 (d, *J* = 18.6 Hz, 1H), 3.17 (d, *J* = 18.8 Hz, 1H) ppm. **^{13}C NMR** (126 MHz, $CDCl_3$) δ 163.2, 153.3, 150.9, 150.4, 143.0, 139.0, 137.9, 135.0, 132.9, 131.4, 130.9, 129.3, 129.2, 129.1, 128.9, 128.4, 128.2, 127.8, 127.6, 127.1, 123.1, 113.4, 52.2, 51.9, 26.4 ppm. **HRMS** (ESI) m/z calculated for $C_{31}H_{25}N_7O$ [M+H]$^+$: 512.2199, found [M+H]$^+$: 512.2193.

2-cyano-N-((1-(1-(2-fluorophenyl)cyclopropyl)-1H-tetrazol-5-yl)(pyridin-3-yl)methyl)-N-(2-methyl-[1,1'-biphenyl]-4-yl)acetamide (3b)

Synthesis according to procedure **C** afforded **3b** (17.9 mg, 0.033 mmol, 31 %) as a white solid; **mp:** 182 - 186 ˚C; **^{1}H NMR** (500 MHz, $CDCl_3$) δ 8.32 – 8.23 (m, 2H), 7.91 – 7.84 (m, 1H), 7.40 – 7.31 (m, 3H), 7.30 – 7.17 (m, 2H), 7.17 – 7.12 (m, 2H), 7.09 – 7.02 (m, 1H), 7.02 – 6.94 (m, 1H), 6.94 – 6.88 (m, 2H), 6.80 (dt, *J* = 8.6, 4.5 Hz, 1H), 6.64 (td, *J* = 9.4, 8.1, 2.3 Hz, 1H), 6.45 – 6.40 (m, 1H), 3.35 – 3.22 (m, 2H), 2.24 (s, 3H), 2.23 – 2.12 (m, 2H), 2.03 (dt, *J* = 10.6, 6.1 Hz, 1H), 1.51 (ddd, *J* = 10.6, 7.5, 5.5 Hz, 1H) ppm. **^{13}C NMR** (126 MHz, $CDCl_3$) δ 163.0, 161.6, 159.6, 154.8, 154.7, 151.3, 149.9, 143.5, 140.1, 138.0, 137.3, 137.1, 135.4, 132.7, 132.2, 131.5, 131.0, 130.9, 130.8, 130.3, 128.9, 128.5, 128.3, 127.7, 127.5, 124.6, 124.5, 123.7, 123.6, 122.8, 122.7, 116.3,

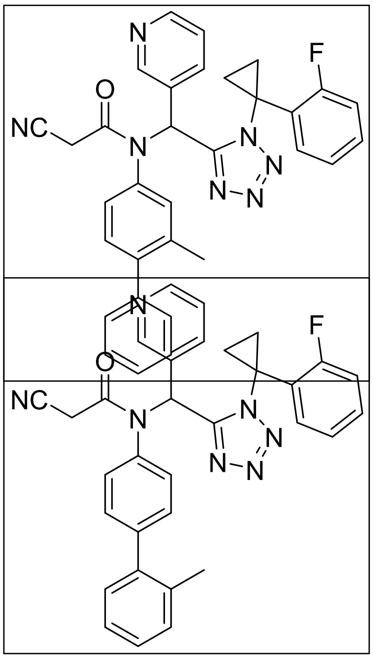

116.1, 113.6, 53.3, 37.9, 26.6, 20.6, 20.2, 15.7, 13.9 ppm. **HRMS** (ESI) m/z calculated for $C_{32}H_{26}FN_7O$ $[M+H]^+$: 544.2261, found $[M+H]^+$: 544.2256.

2-cyano-N-((1-(1-(2-fluorophenyl)cyclopropyl)-1H-tetrazol-5-yl)(pyridin-3-yl)methyl)-N-(2'-methyl-[1,1'-biphenyl]-4-yl)acetamide (3c)

Synthesis according to procedure **C** afforded **3c** (17.9 mg, 0.033 mmol, 23 %) as a white solid; **mp:** 198 - 202 ˚C; **^{1}H NMR** (500 MHz, $CDCl_3$) δ 8.28 (d, *J* = 2.3 Hz, 1H), 8.23 (dt, *J* = 4.9, 1.3 Hz, 1H), 8.15 (d, *J* = 8.1 Hz, 1H), 7.32 (d, *J* = 8.2 Hz, 1H), 7.28 – 7.18 (m, 6H), 7.09 – 7.05 (m, 1H), 7.02 (ddd, *J* = 14.6, 6.7, 1.9 Hz, 1H), 6.95 – 6.88 (m, 2H), 6.74 (dd, *J* = 8.0, 4.8 Hz, 1H), 6.62 (ddd, *J* = 10.6, 8.2, 1.7 Hz, 2H), 3.34 (d, *J* = 18.7 Hz, 1H), 3.26 (d, *J* = 18.8 Hz, 1H), 2.28 – 2.13 (m, 2H), 2.10 – 2.00 (m, 4H), 1.50 (ddd, *J* = 10.7, 7.3, 5.3 Hz, 1H) ppm. **^{13}C NMR** (126 MHz, $CDCl_3$) δ 162.9, 161.6, 159.6, 154.8, 151.3, 149.9, 143.7, 140.0, 137.3, 135.2, 135.0, 131.1, 131.04, 131.0, 130.9, 130.6, 130.4, 130.35, 130.33, 130.31, 129.4, 128.0, 127.6, 126.0, 124.6, 124.5, 123.69, 123.60, 122.7, 116.3, 116.1, 113.5, 53.5, 37.9, 26.6, 20.3, 15.8, 13.9, 13.8 ppm. **HRMS** (ESI) m/z calculated for $C_{32}H_{26}FN_7O$ $[M+H]^+$: 544.2261, found $[M+H]^+$: 544.2256.

2-methyl-[1,1'-biphenyl]-4-amine (4a)

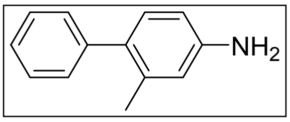

Synthesis according to procedure **D** afforded **4a** (3.62 g, 19.8 mmol, 73 %) as a dark red/brown oil. **^{1}H NMR** (500 MHz, $CDCl_3$) δ 7.39 (m, 2H), 7.35 – 7.28 (m, 3H), 7.06 (d, *J* = 8.0 Hz, 1H), 6.64 – 6.57 (m, 2H), 3.66 (s, 2H), 2.23 (s, 3H) ppm.

2-(trifluoromethyl)-[1,1'-biphenyl]-4-amine (4b)

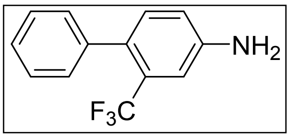

Synthesis according to procedure **D** afforded **4b** (3.32 g, 14.0 mmol, 67 %) as a dark red/brown oil. **^{1}H NMR** (500 MHz, $CDCl_3$) δ 7.37 – 7.26 (m, 5H), 7.07 (d, *J* = 8.2 Hz, 1H), 6.98 (d, *J* = 2.5 Hz, 1H), 6.75 (dd, *J* = 8.2, 2.5 Hz, 1H), 3.76 (s, 2H) ppm.

2-chloro-[1,1'-biphenyl]-4-amine (4c)

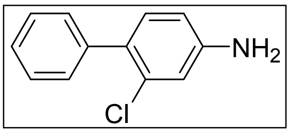

Synthesis according to procedure **D** afforded **4c** (0.54 g, 2.65 mmol, 67 %) as a dark red/brown oil. **^{1}H NMR** (500 MHz, $CDCl_3$) δ 7.44 – 7.36 (m, 5H), 7.33 – 7.29 (m, 1H), 7.11 (d, *J* = 8.2 Hz, 1H), 6.76 (d, *J* = 2.4 Hz, 1H), 6.58 (dd, *J* = 8.2, 2.4 Hz, 1H), 3.70 (s, 2H) ppm.

2'-methyl-[1,1'-biphenyl]-4-amine (4d)

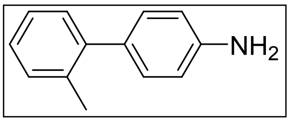

Synthesis according to procedure **D** afforded **4d** (2.50 g, 13.6 mmol, 75 %) as a dark red/brown oil. **^{1}H NMR** (400 MHz, CHLOROFORM-*D*) δ 7.25 – 7.18 (m, 4H), 7.17 – 7.10 (m, 2H), 6.79 – 6.72 (m, 2H), 3.84 (s, 2H), 2.29 (s, 3H) ppm.

2'-(trifluoromethyl)-[1,1'-biphenyl]-4-amine (4e)

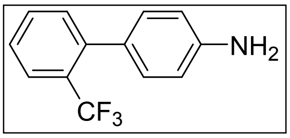

Synthesis according to procedure **D** afforded **4e** (2.30 g, 9.69 mmol, 71 %) as a dark red/brown oil. **^{1}H NMR** (400 MHz, CHLOROFORM-*D*) δ 7.71 (dd, *J* = 7.9, 1.3 Hz, 1H), 7.52 (t, *J* = 7.5 Hz, 1H), 7.41 (t, *J* = 7.7 Hz, 1H), 7.32 (d, *J* = 7.6 Hz, 1H), 7.13 (d, *J* = 8.1 Hz, 2H), 6.76 – 6.68 (m, 2H), 3.88 (s, 2H) ppm.

2'-chloro-[1,1'-biphenyl]-4-amine (4f)

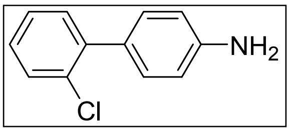

Synthesis according to procedure **D** afforded **4f** (3.30 g, 16.2 mmol, 81 %) as a dark red/brown oil. **^{1}H NMR** (400 MHz, CHLOROFORM-*D*) δ 7.44 (dd, *J* = 7.8, 1.5 Hz, 1H), 7.37 – 7.17 (m, 4H), 6.80 – 6.72 (m, 2H), 6.62 – 6.52 (m, 1H), 3.80 (s, 2H) ppm.

NMR spectra

N-((1-benzyl-1H-tetrazol-5-yl)(pyridin-3-yl)methyl)-4-(thiophen-2-yl)aniline (1a)

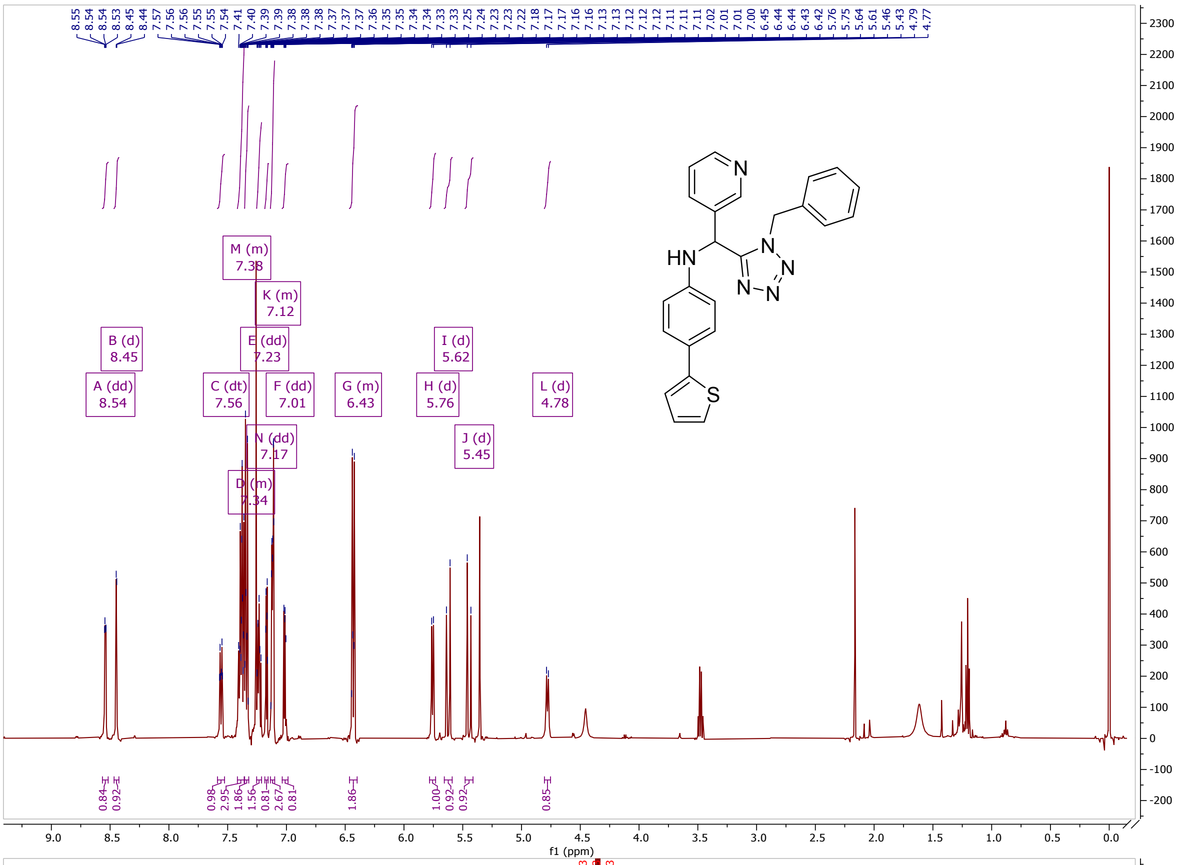
A (dd) 8.54
B (d) 8.45
C (dt) 7.56
D (m) 7.34
E (dd) 7.23
F (dd) 7.01
G (m) 6.43
H (d) 5.76
I (d) 5.62
J (d) 5.45
K (m) 7.12
L (d) 4.78
M (m) 7.38
N (dd) 7.17
HN
N
S
f1 (ppm)

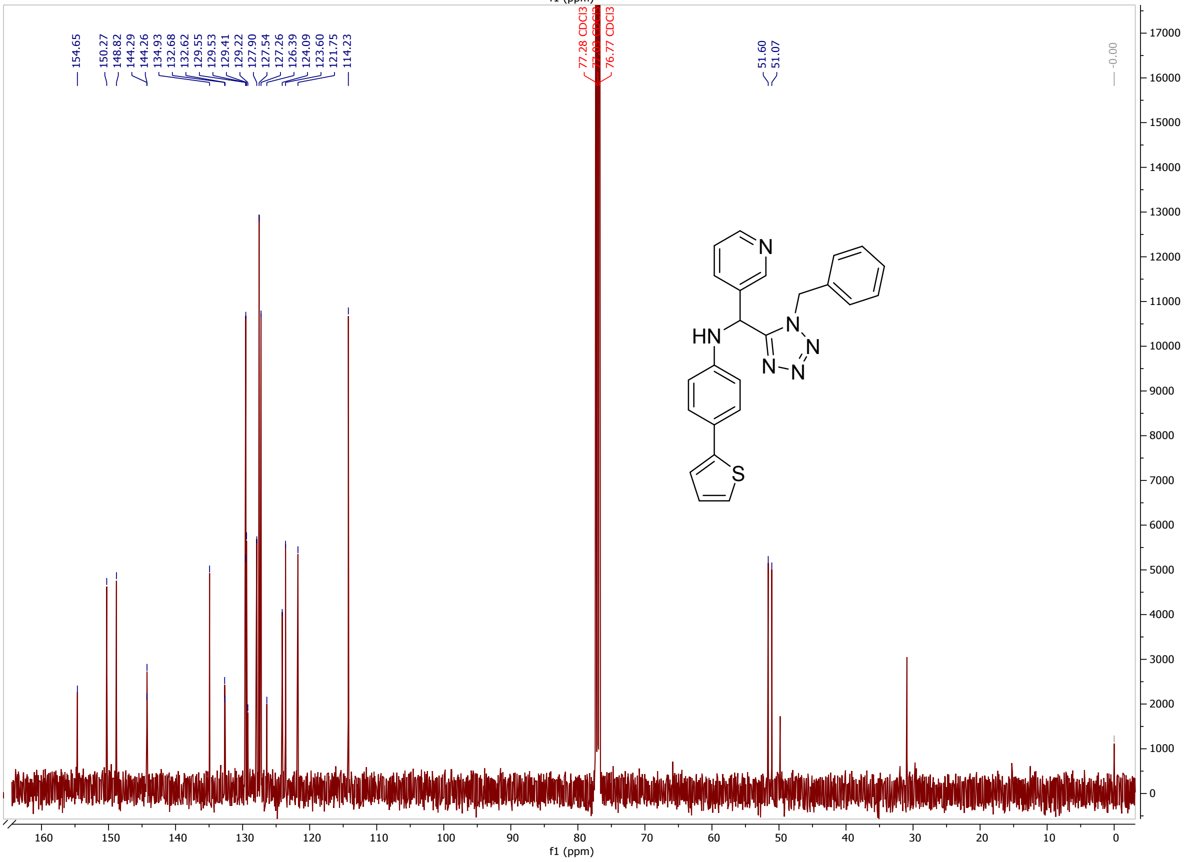
154.65
150.27
148.82
144.29
144.26
134.93
132.68
132.62
129.55
129.53
129.41
129.22
127.90
127.54
127.26
126.39
124.09
123.60
121.75
114.23
77.28 CDCl3
76.77 CDCl3
51.60
51.07
-0.00
HN
N
S
f1 (ppm)

N-((1-benzyl-1H-tetrazol-5-yl)(pyridin-3-yl)methyl)-3-fluoro-4-morpholinoaniline (1b)

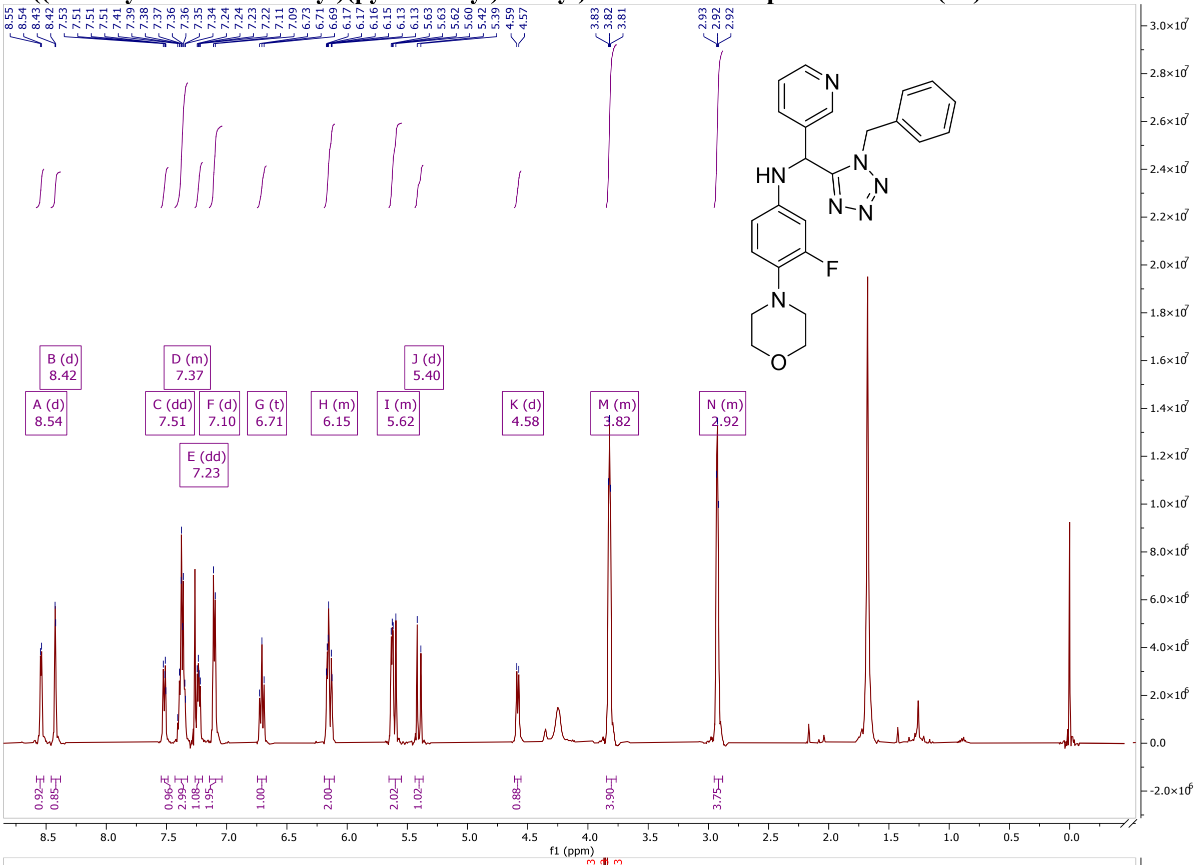

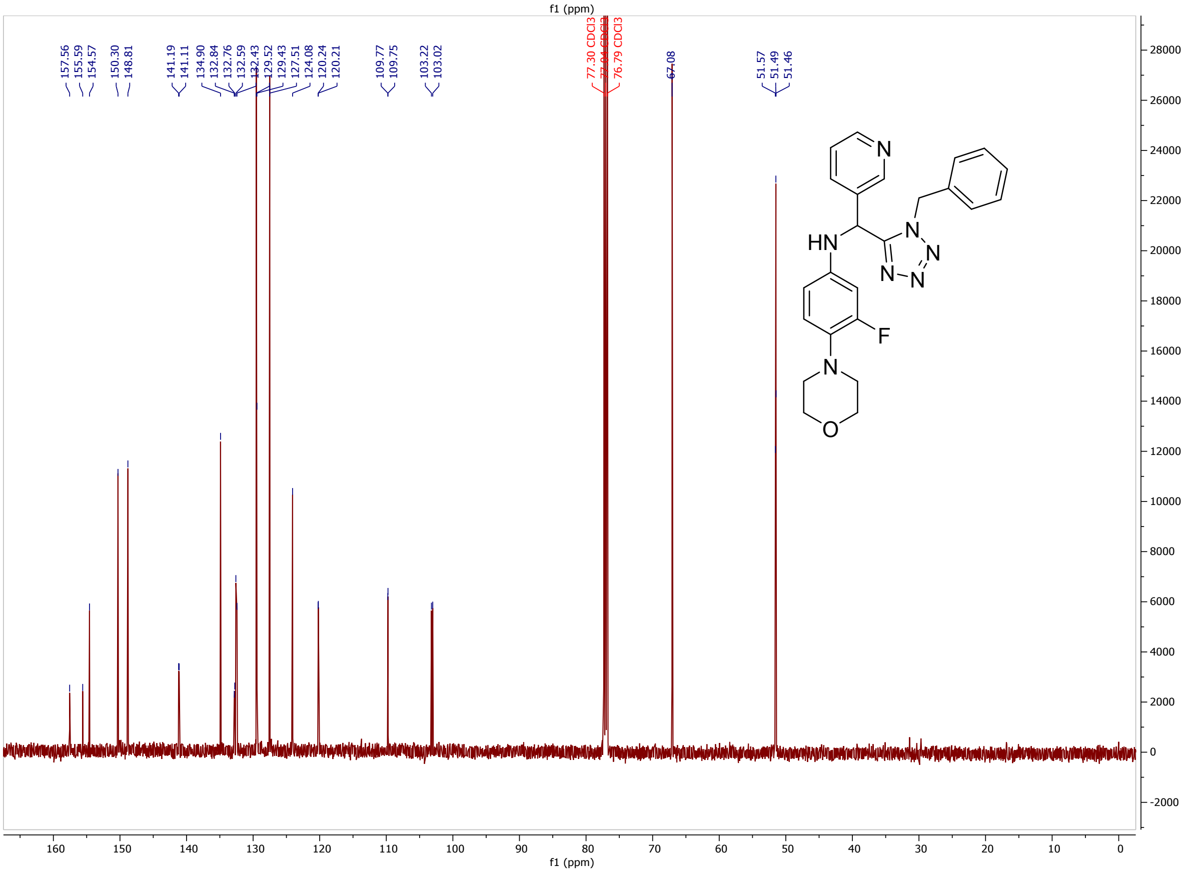

N-((1-benzyl-1H-tetrazol-5-yl)(pyridin-3-yl)methyl)-4-(oxazol-5-yl)aniline (1c)

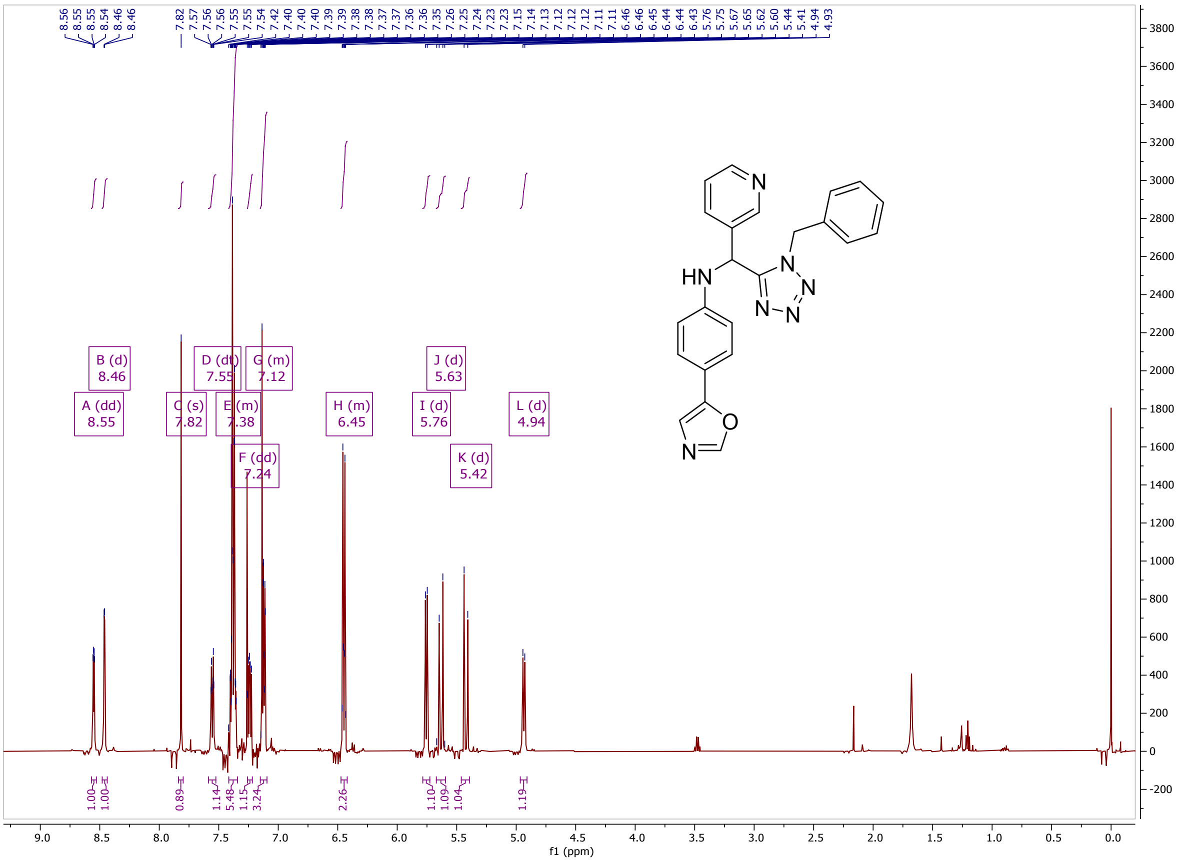

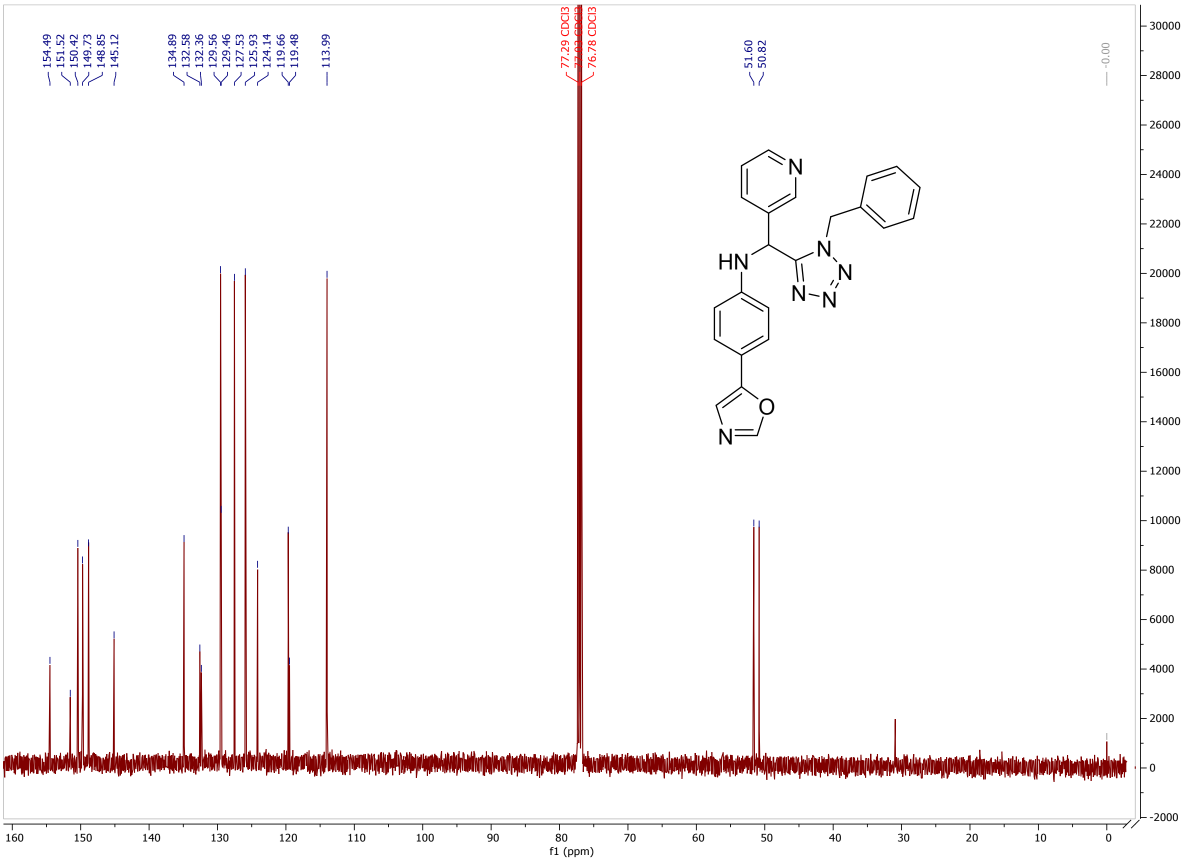

N-((1-benzyl-1H-tetrazol-5-yl)(pyridin-3-yl)methyl)-4-(1H-pyrazol-3-yl)aniline (1d)

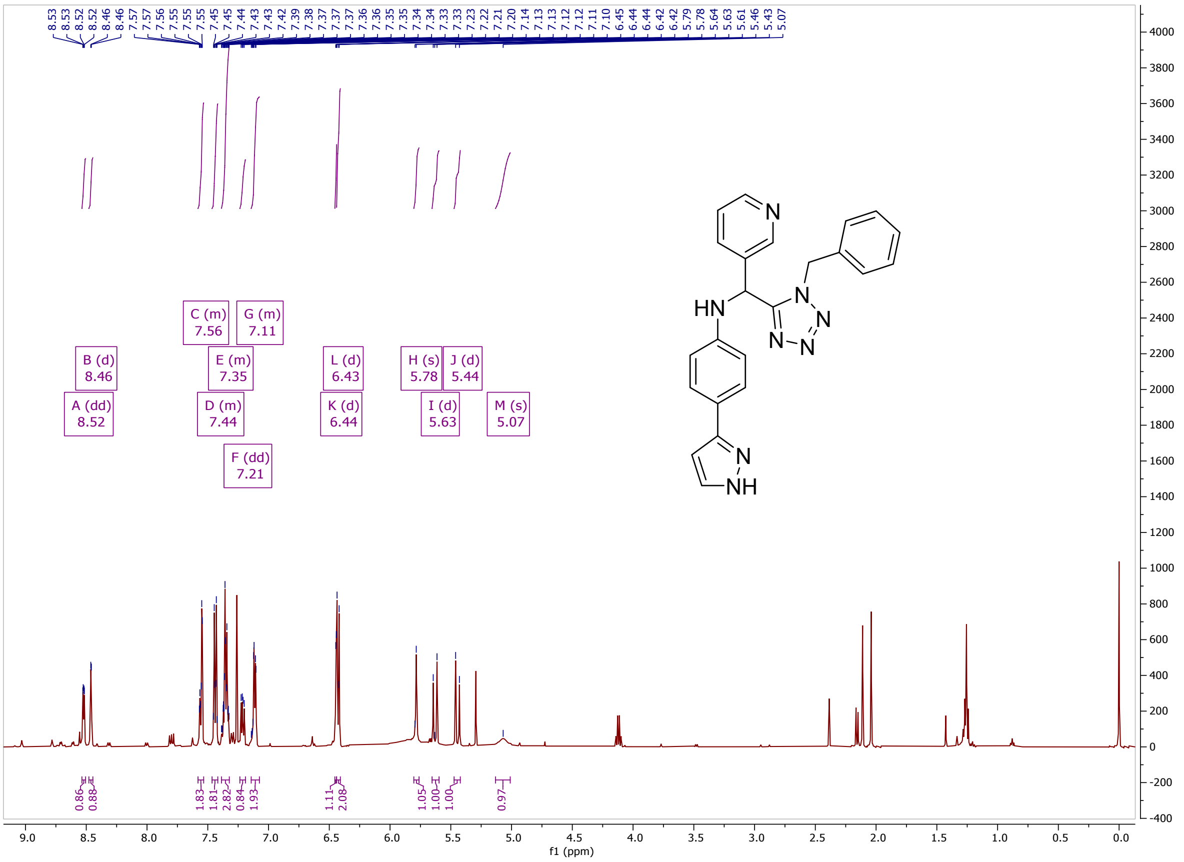

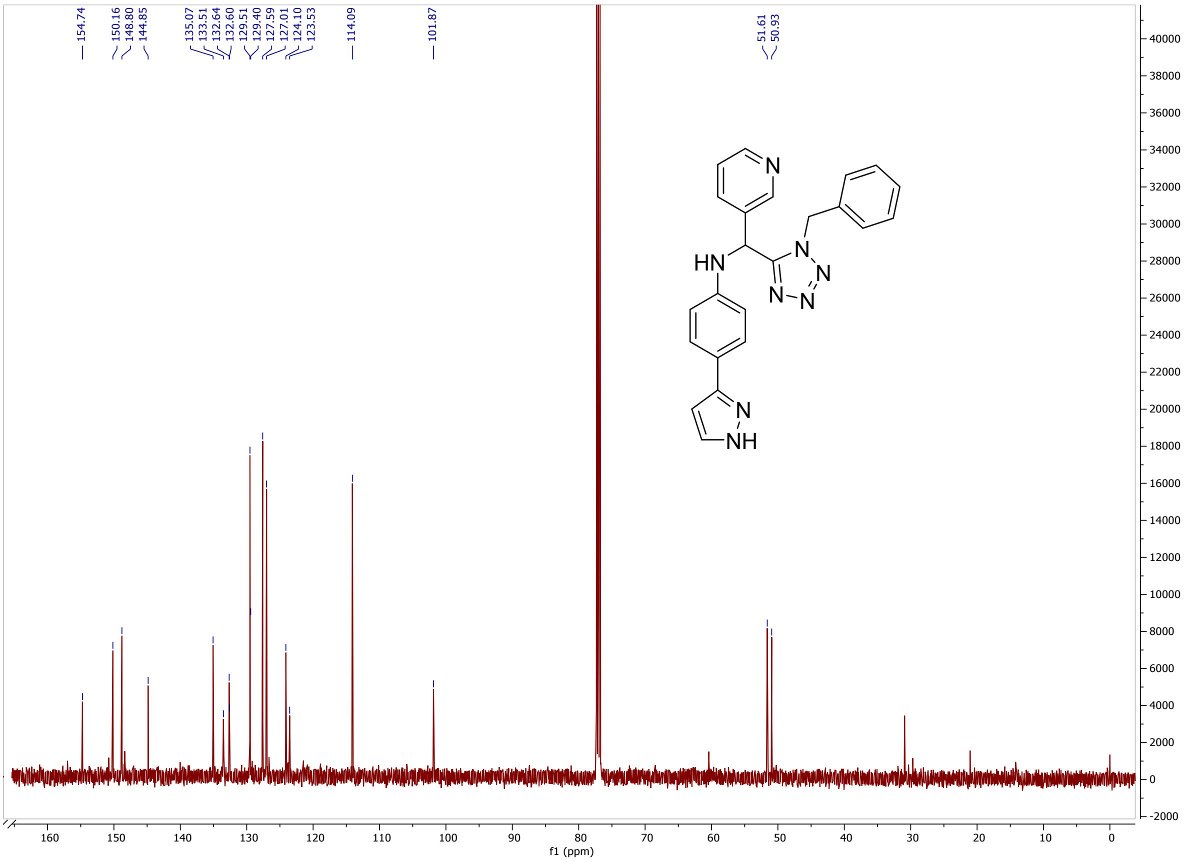

N-((1-benzyl-1H-tetrazol-5-yl)(pyridin-3-yl)methyl)-4-(1,3,4-oxadiazol-2-yl)aniline (1e)

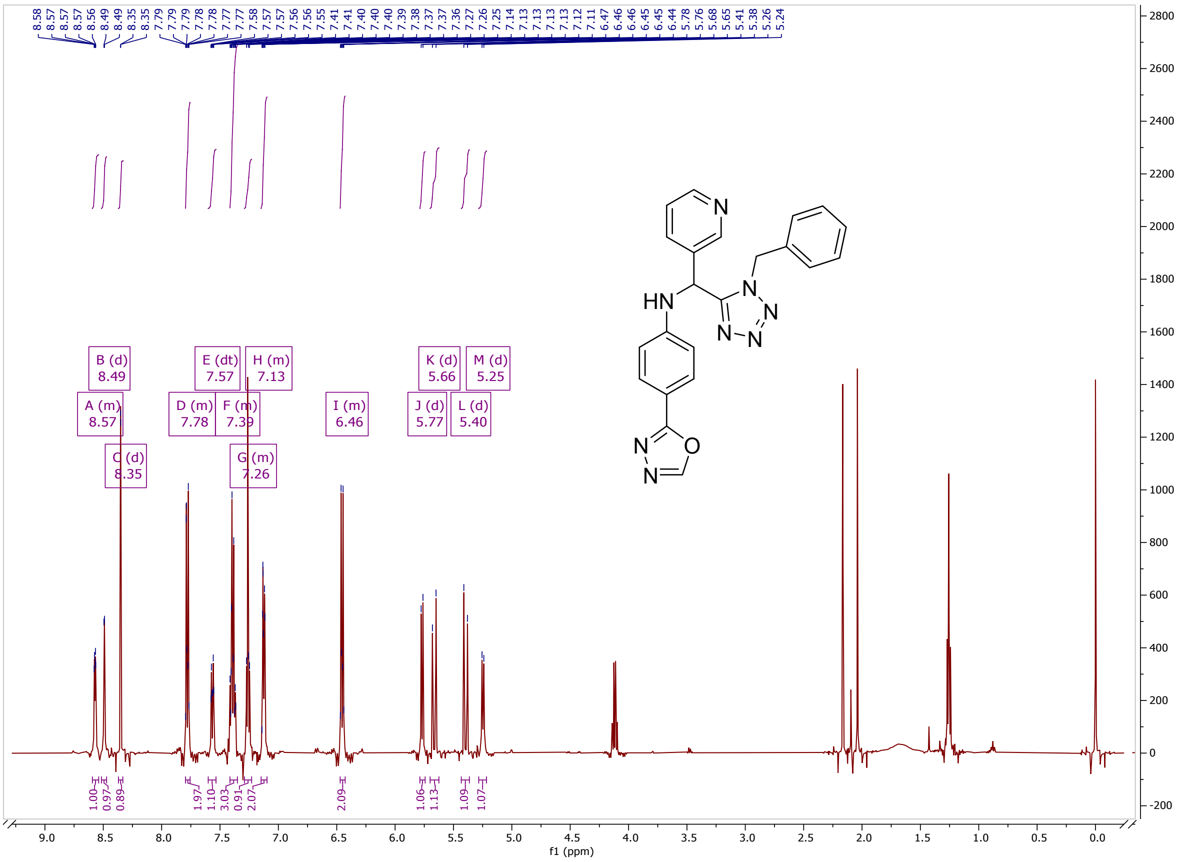

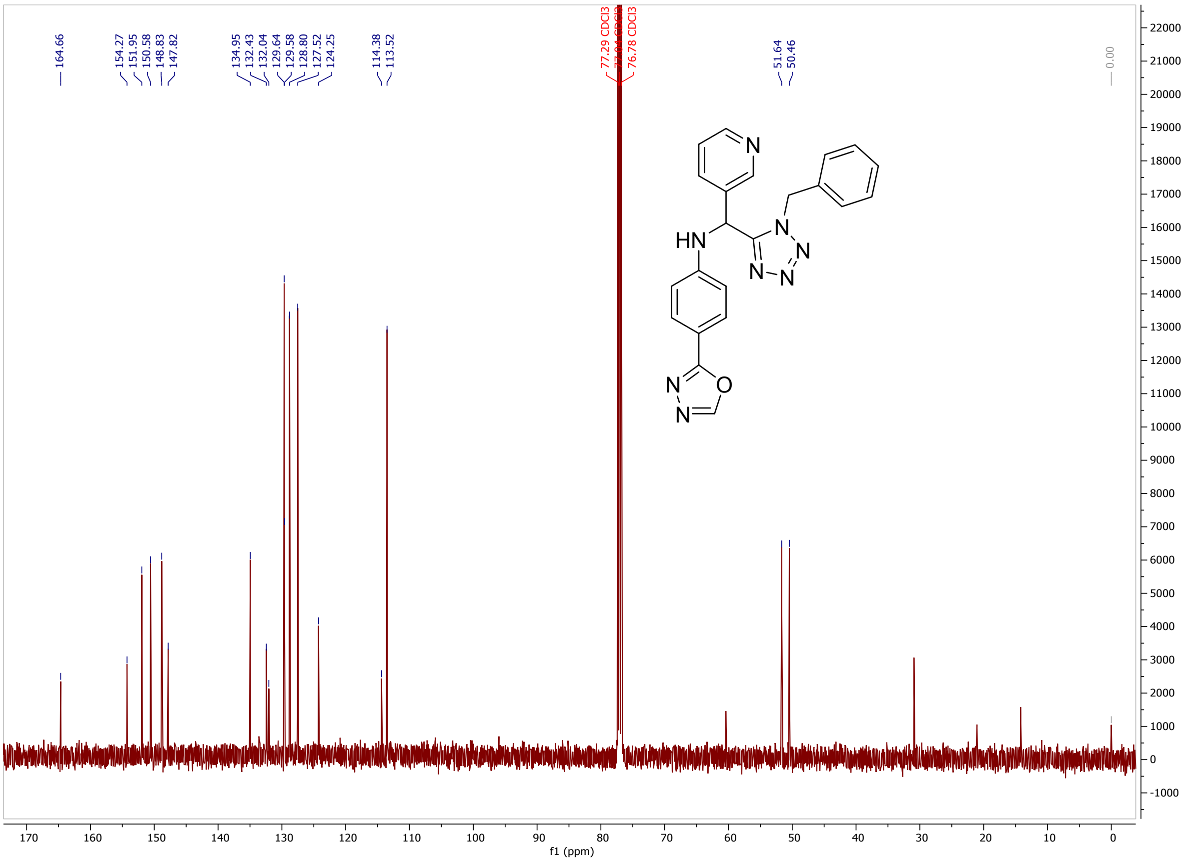

N-((1-benzyl-1H-tetrazol-5-yl)(pyridin-3-yl)methyl)-4-(tert-butyl)aniline (1f)

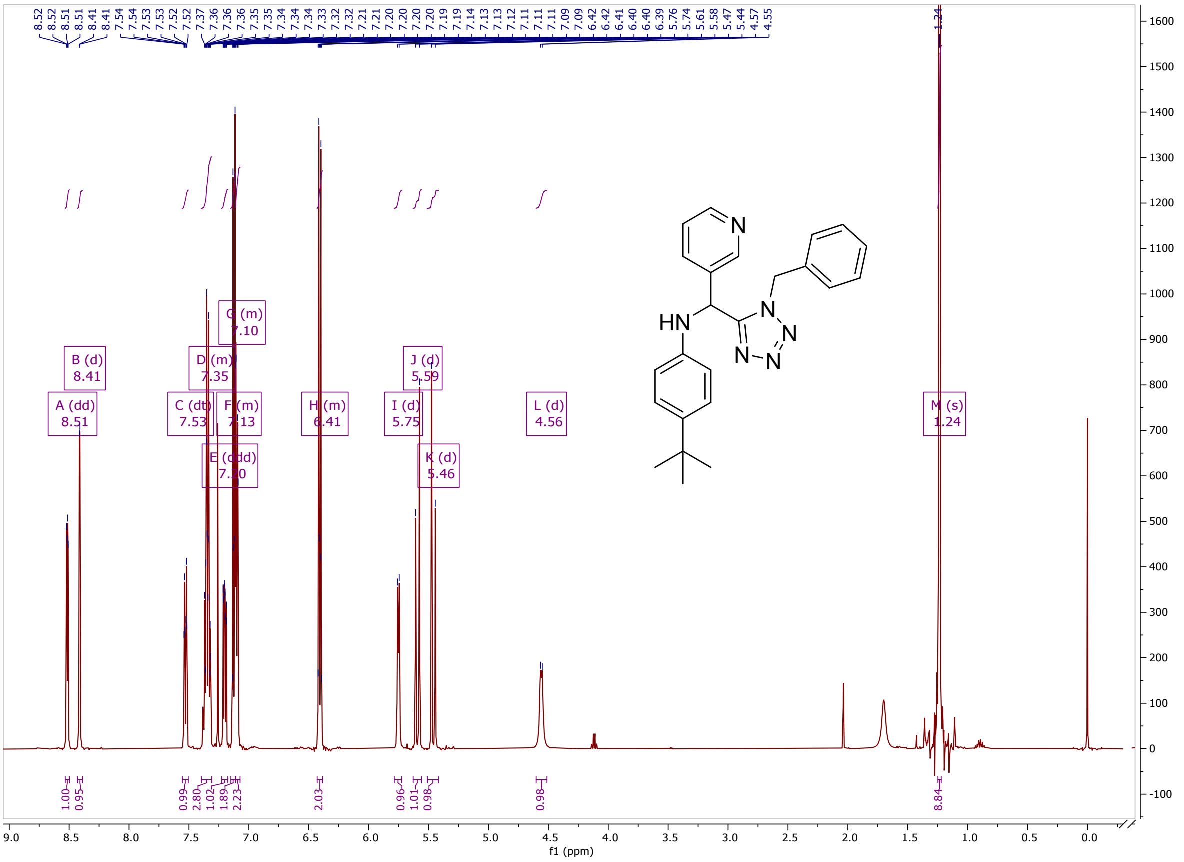

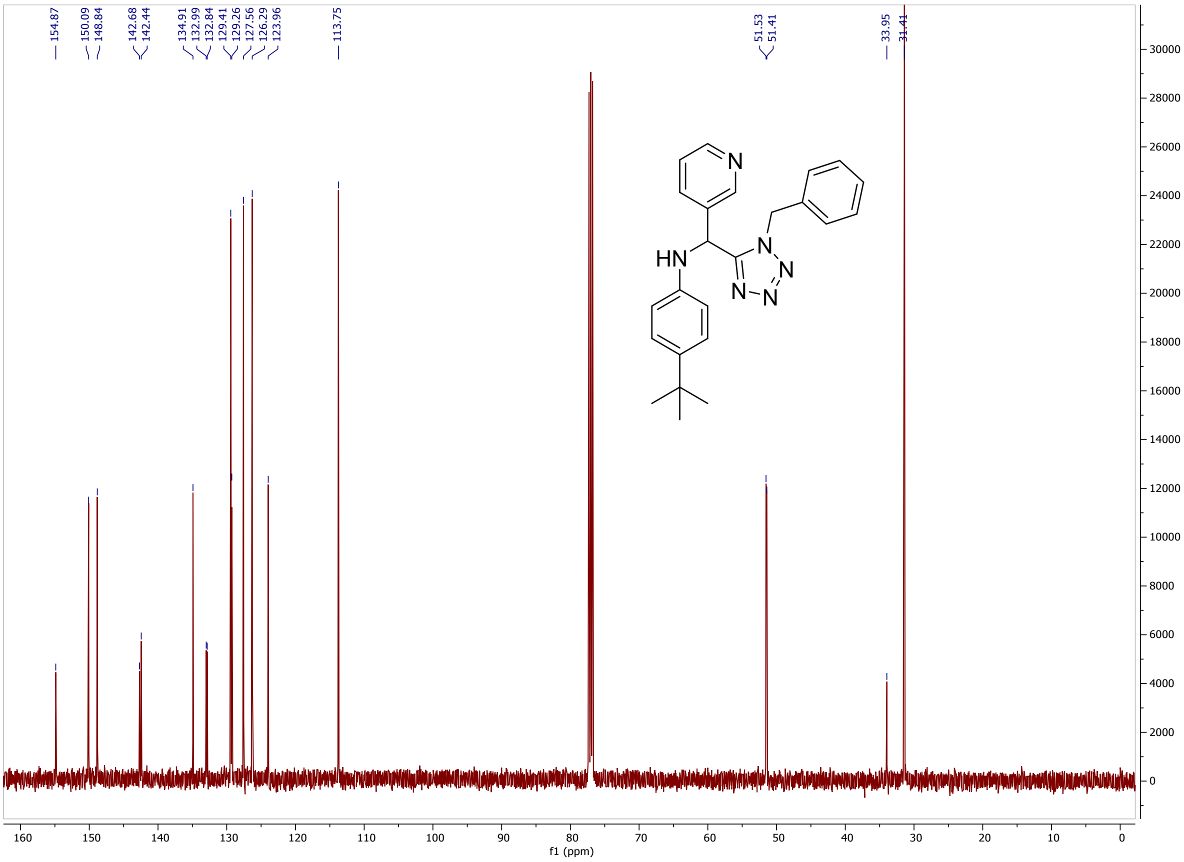

N-((1-benzyl-1H-tetrazol-5-yl)(pyridin-3-yl)methyl)-[1,1'-biphenyl]-4-amine (1g)

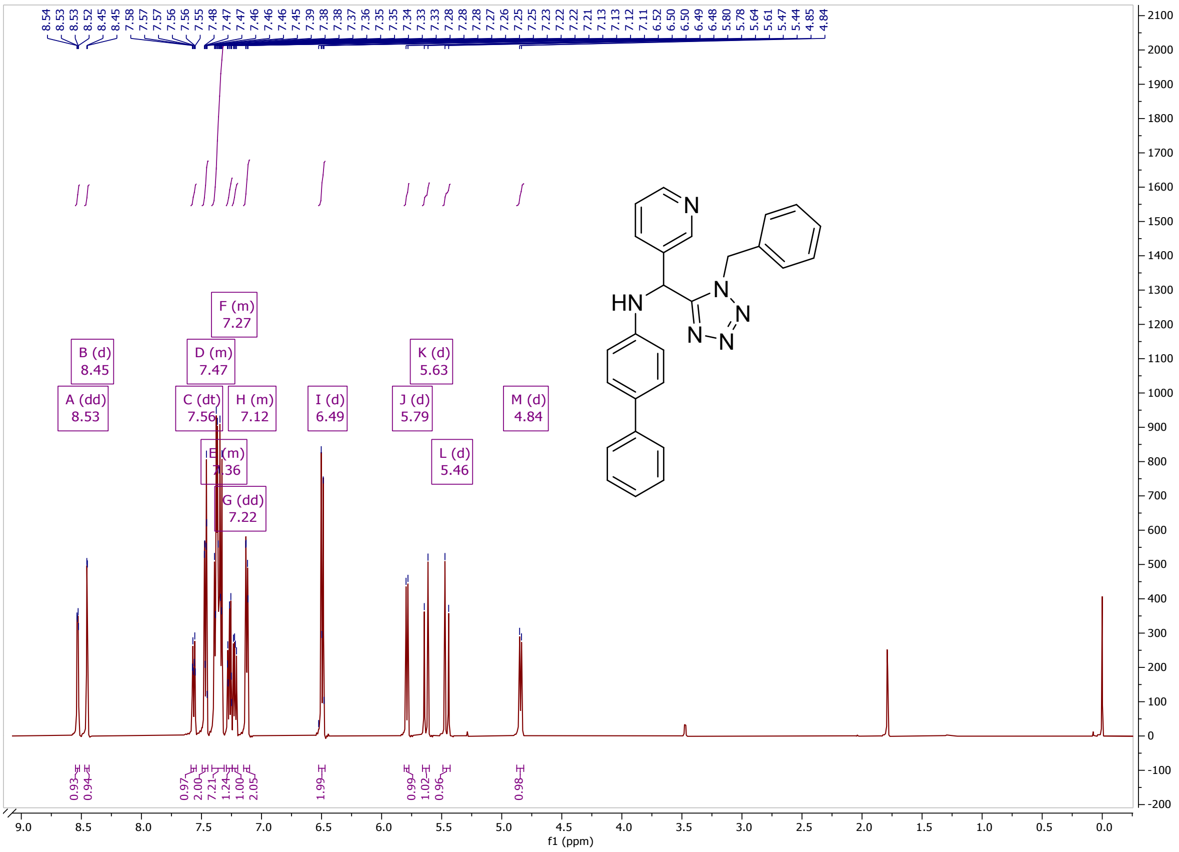

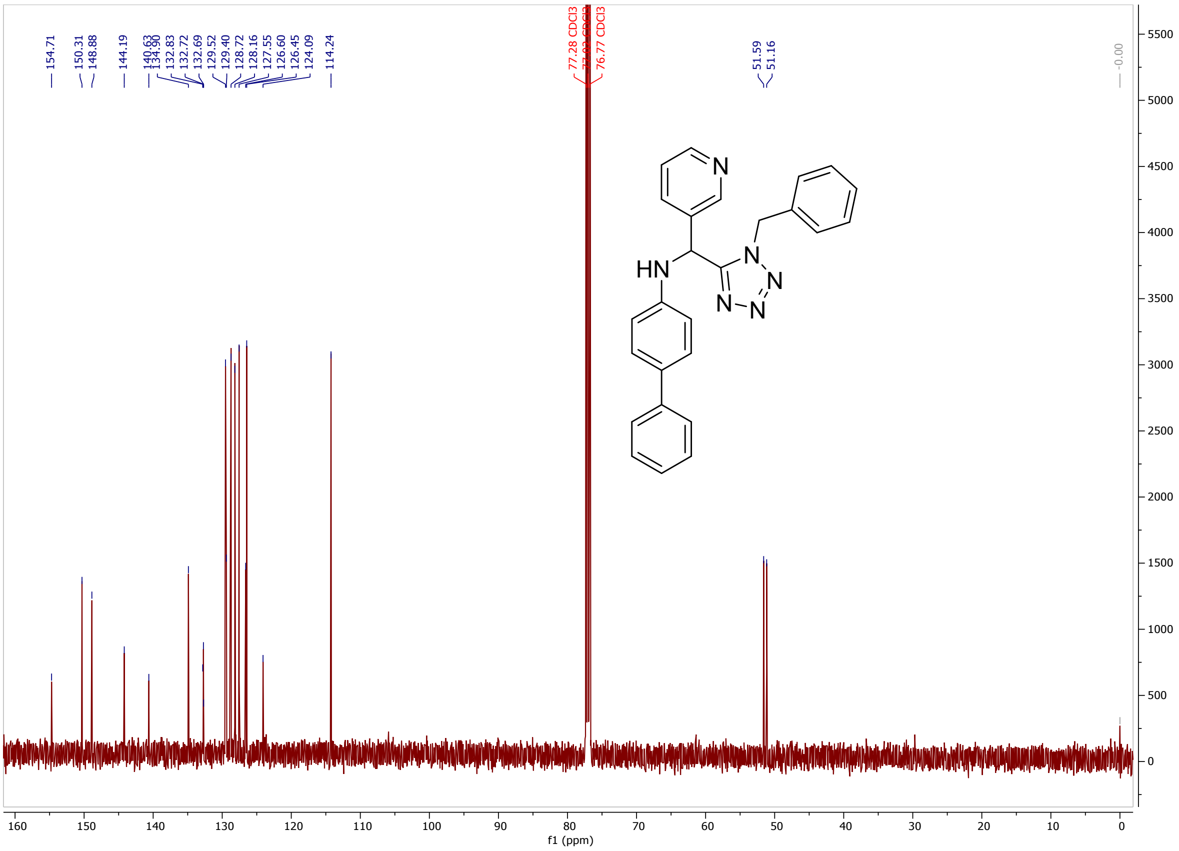

N-((1-benzyl-1H-tetrazol-5-yl)(1H-pyrazol-3-yl)methyl)-4-(tert-butyl)aniline (1h)

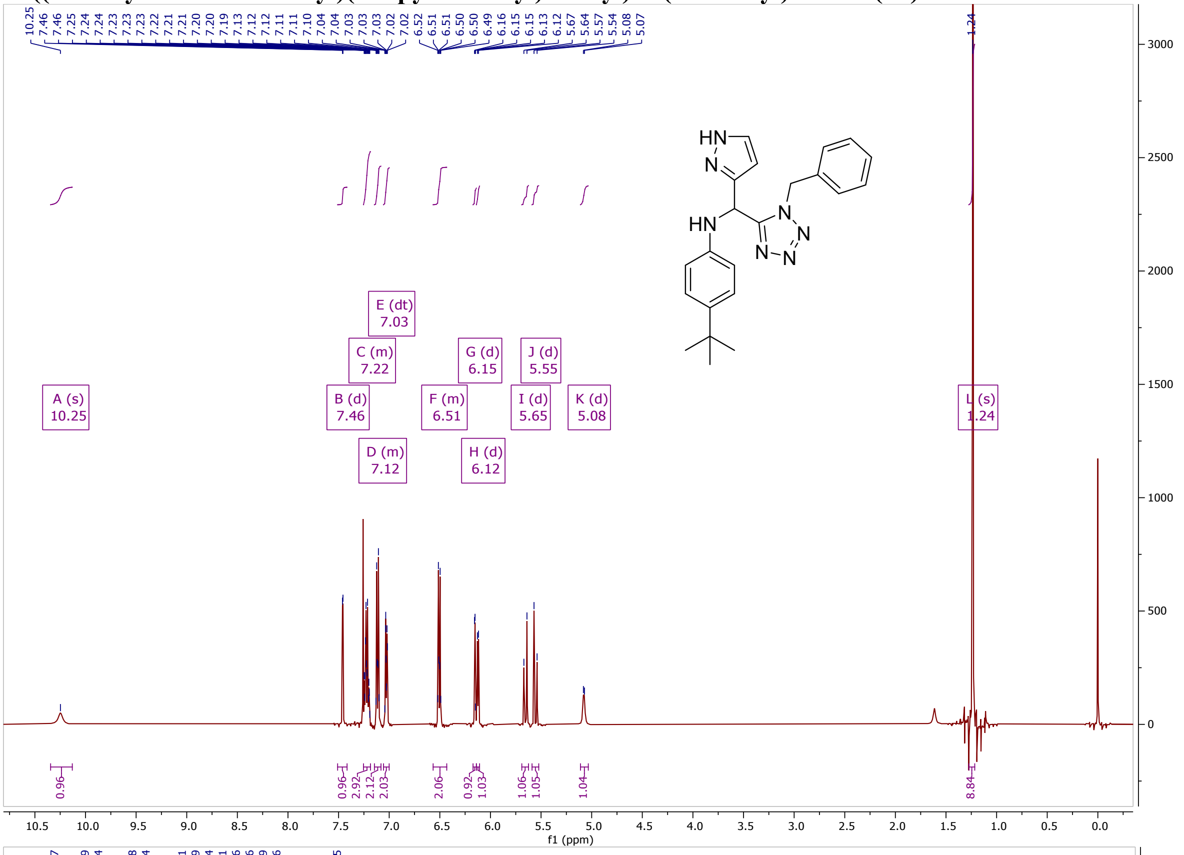

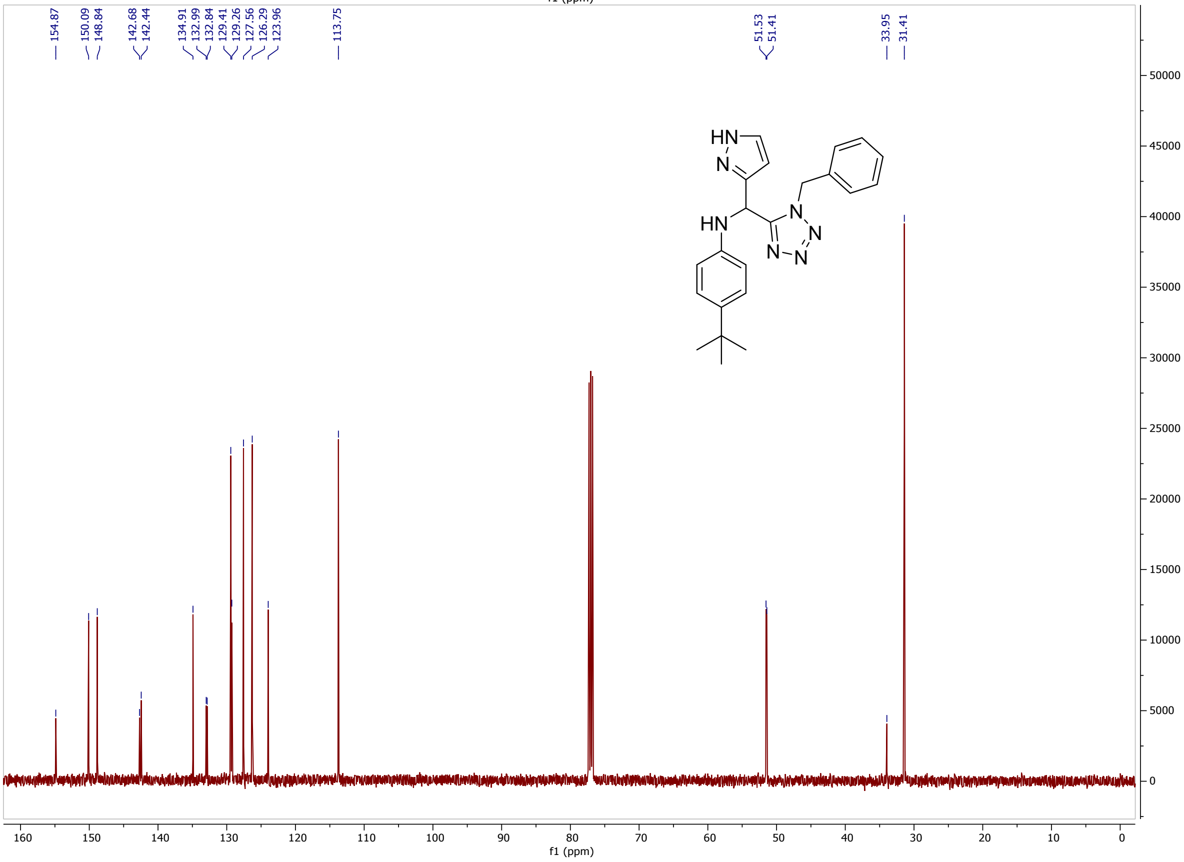

N-((1-benzyl-1H-tetrazol-5-yl)(imidazo[1,2-a]pyridin-3-yl)methyl)-4-(tert-butyl)aniline (1i)

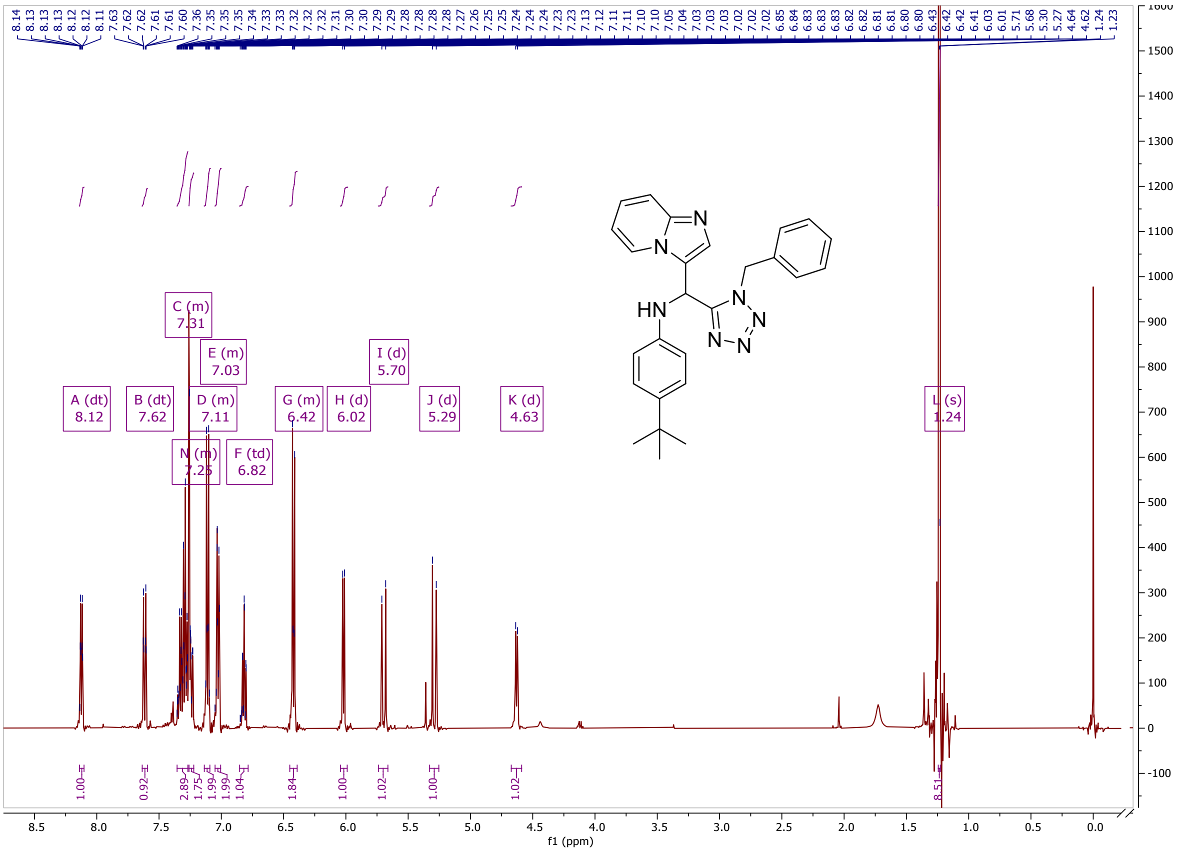

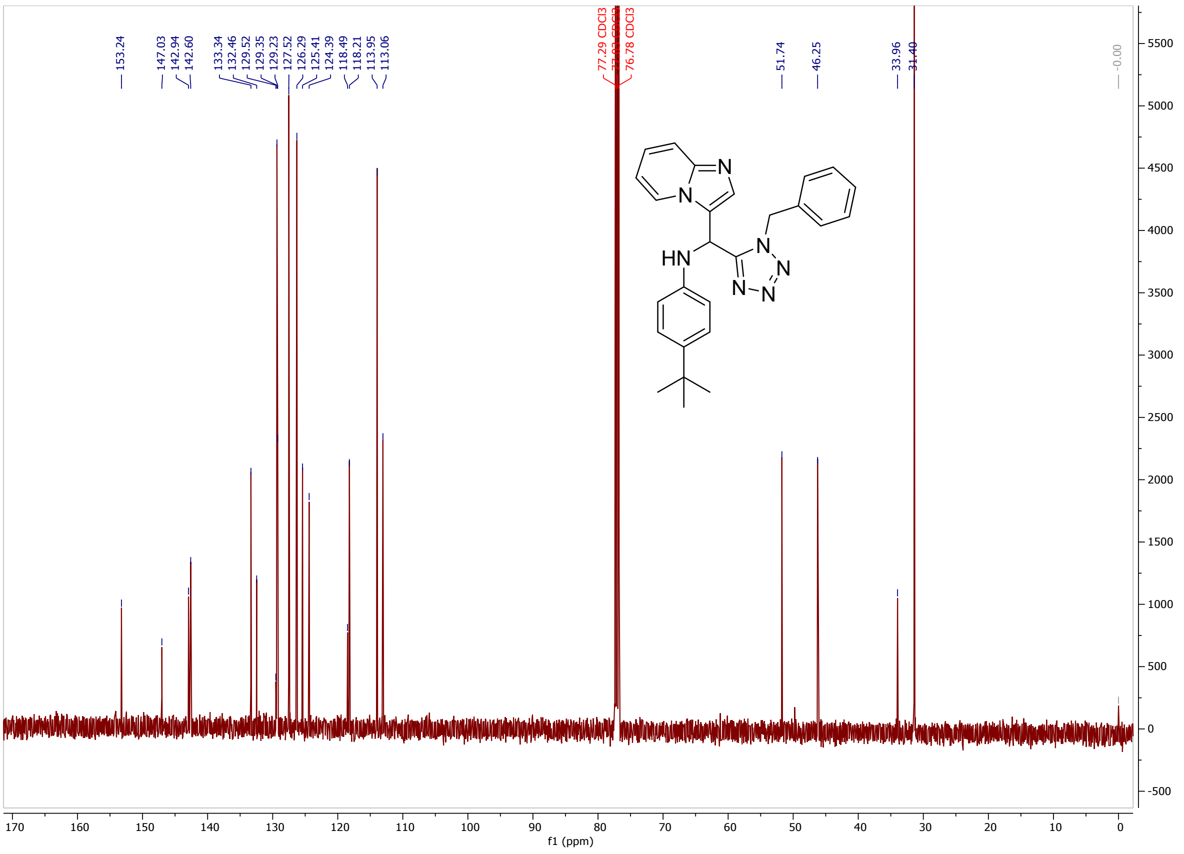

N-((1-benzyl-1H-tetrazol-5-yl)(pyridazin-3-yl)methyl)-4-(tert-butyl)aniline (1j)

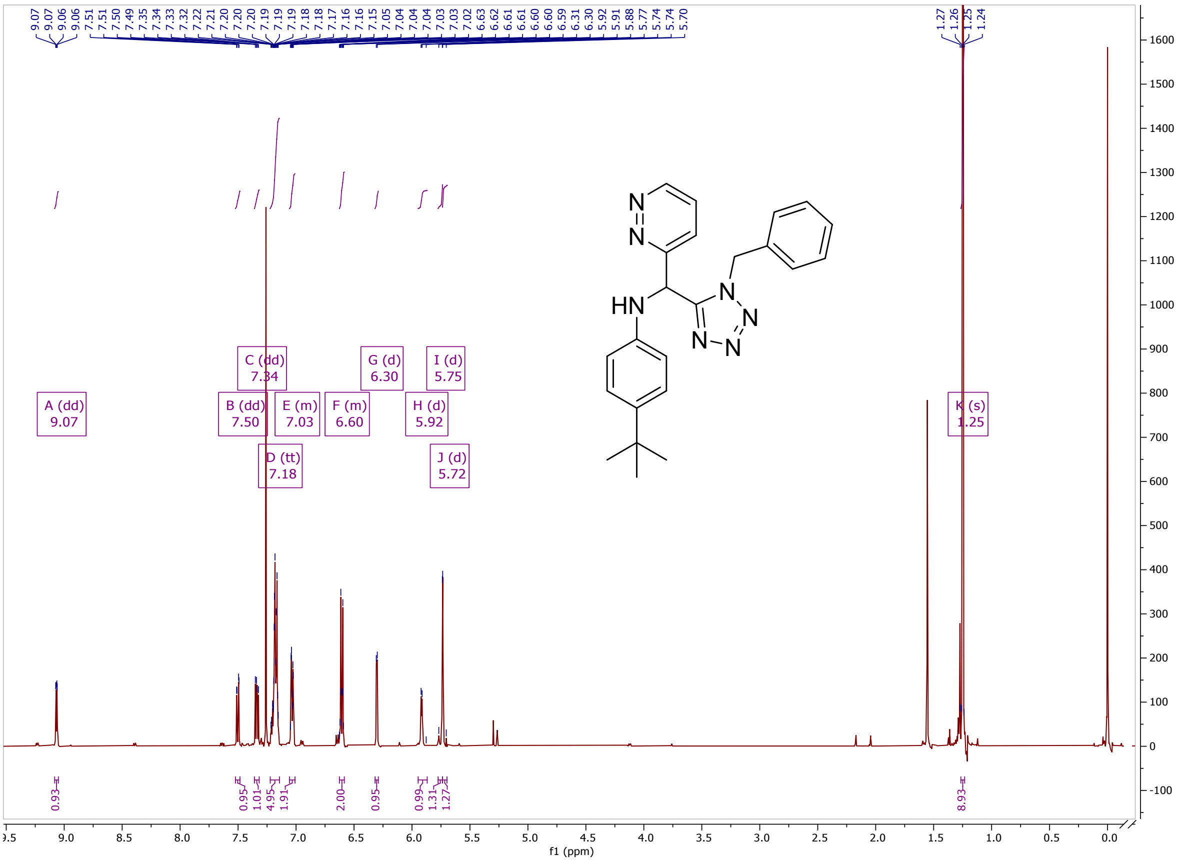

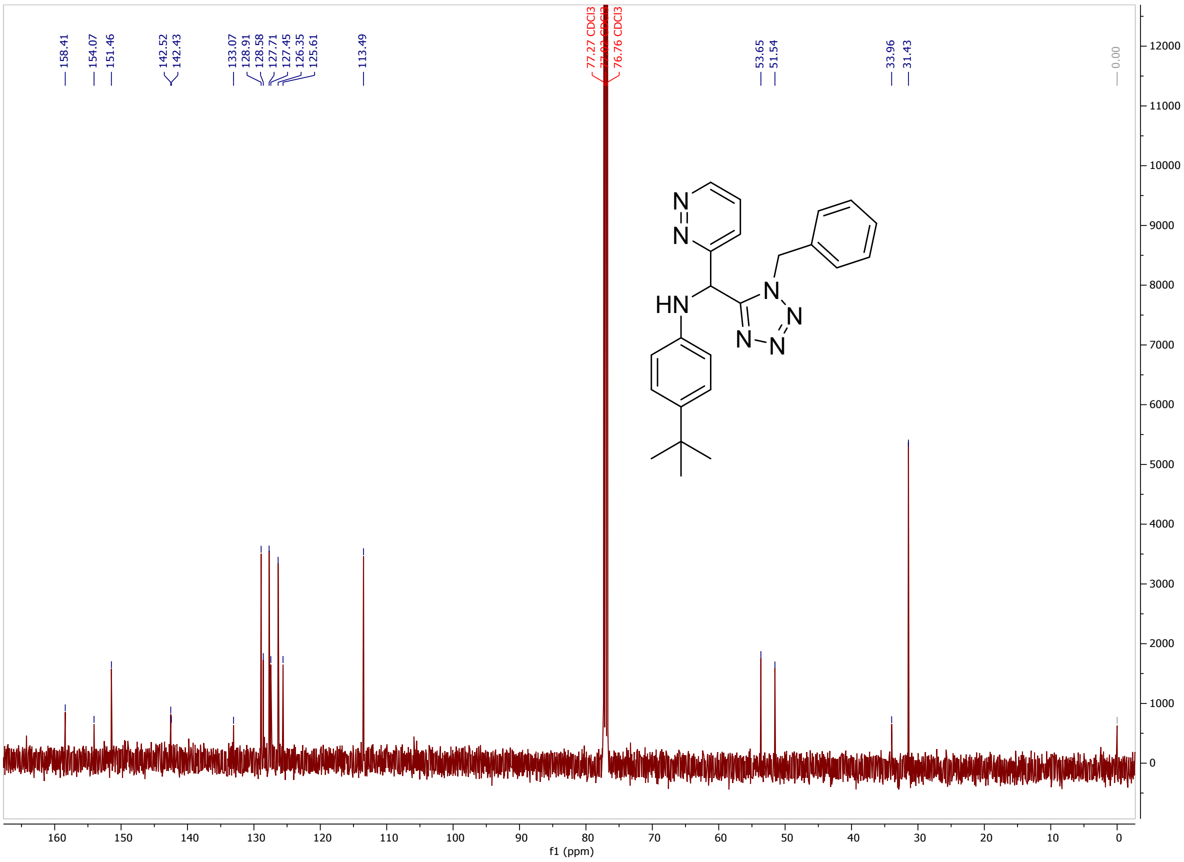

N-((1-benzyl-1H-tetrazol-5-yl)(1-methyl-1H-pyrazol-4-yl)methyl)-4-(tert-butyl)aniline (1k)

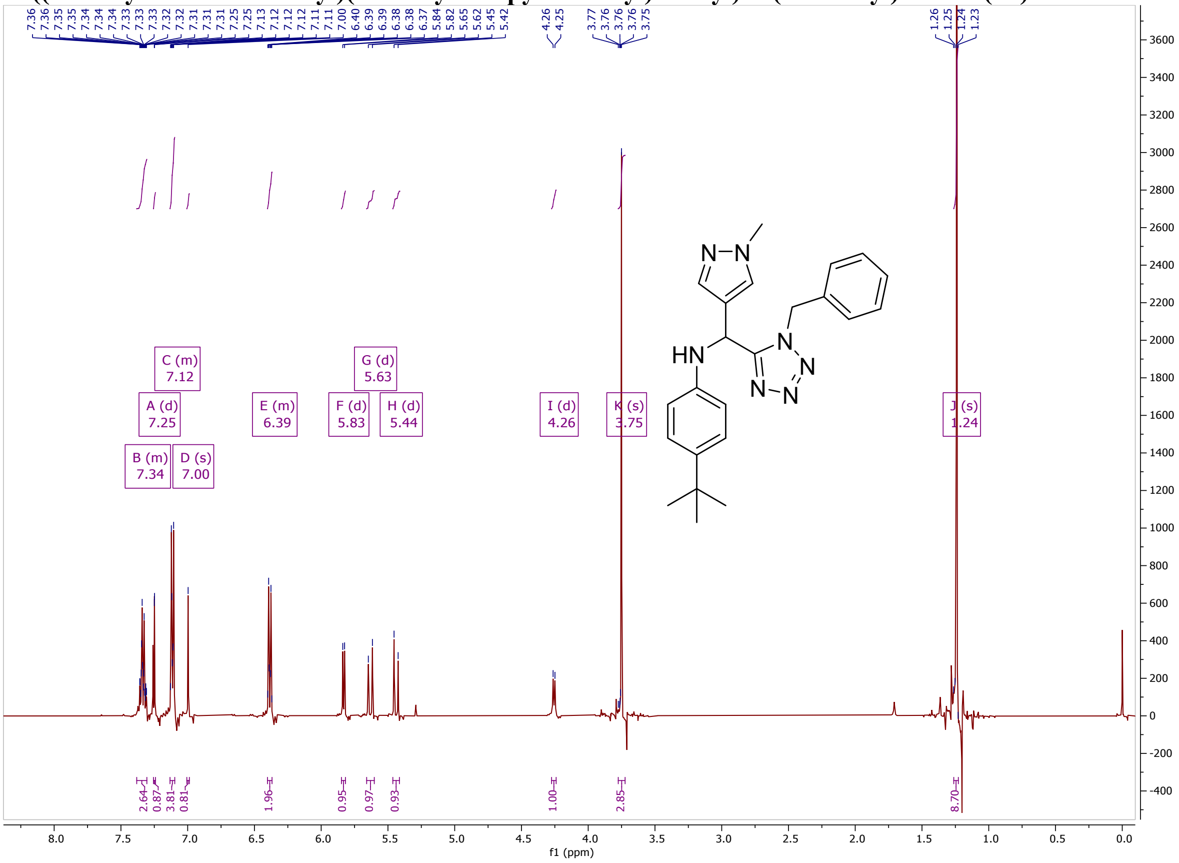

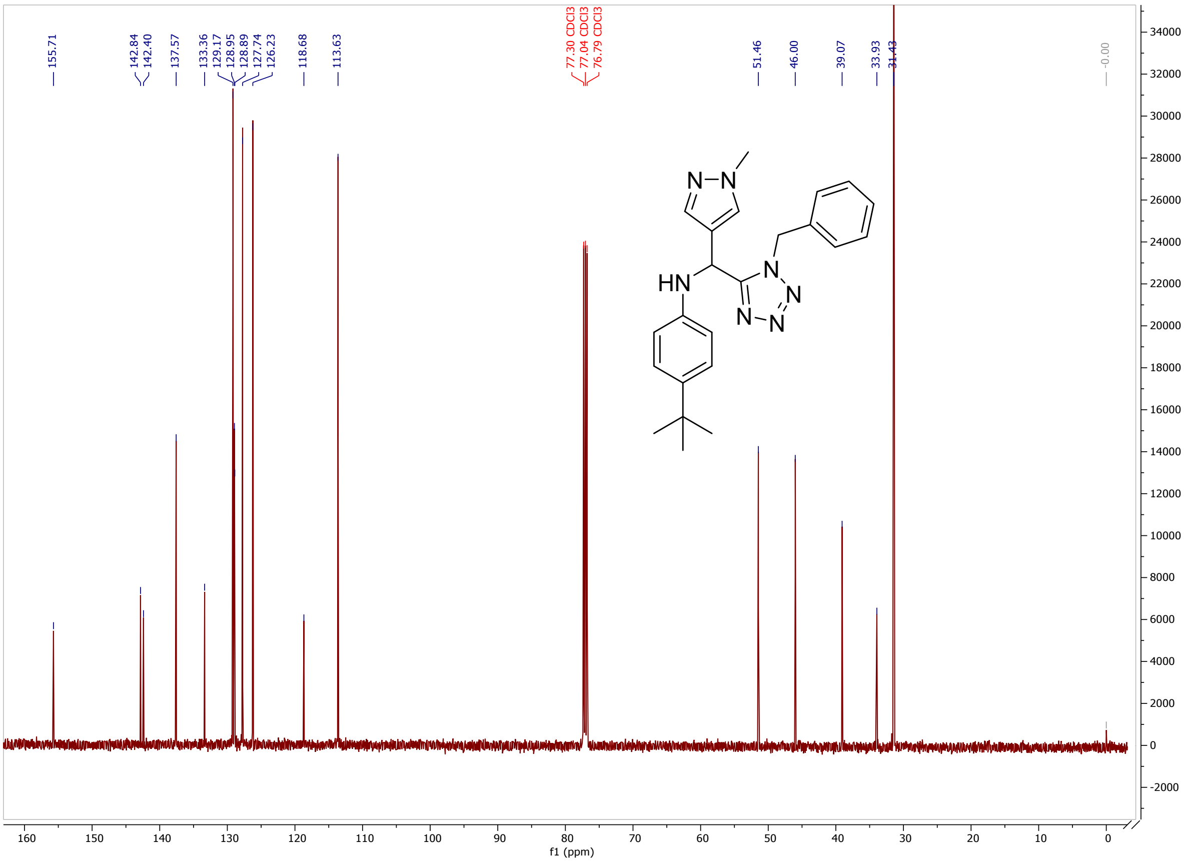

N-((1-benzyl-1H-tetrazol-5-yl)(5-methylpyridin-3-yl)methyl)-4-(tert-butyl)aniline (1l)

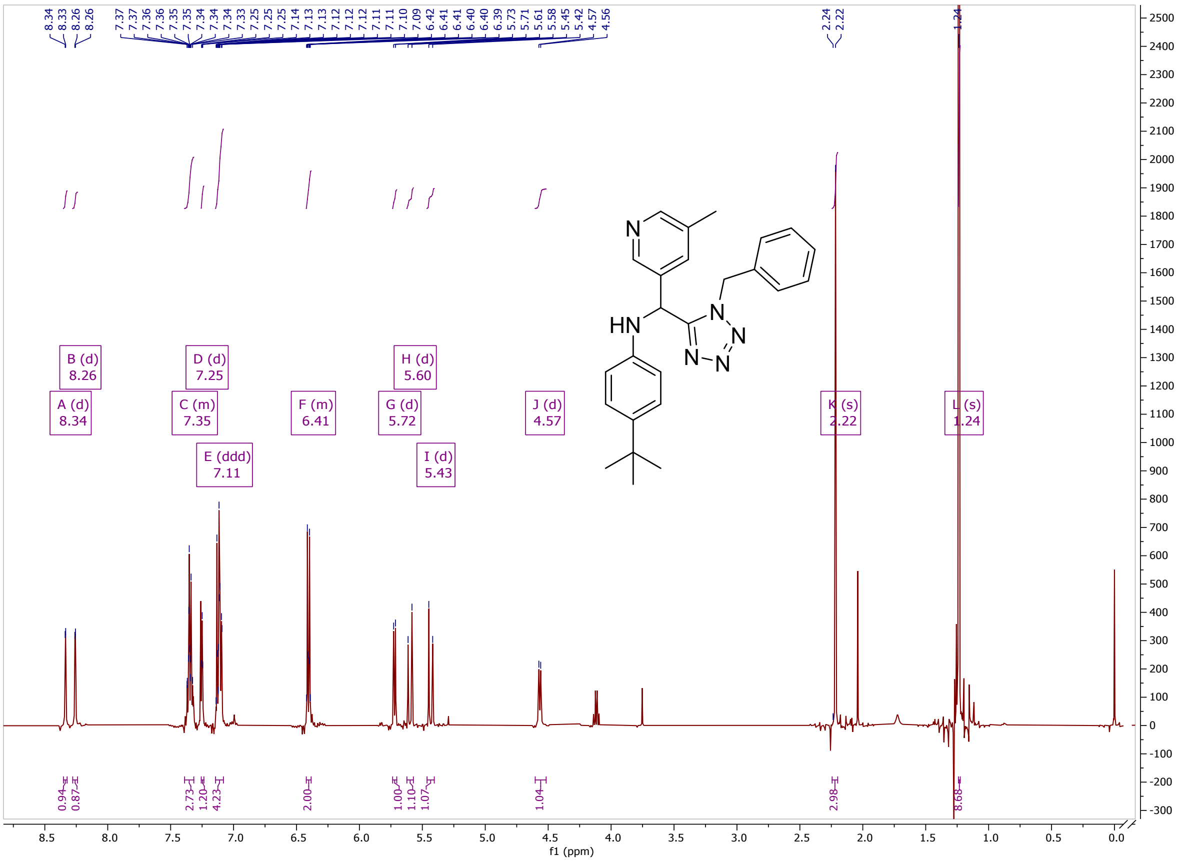

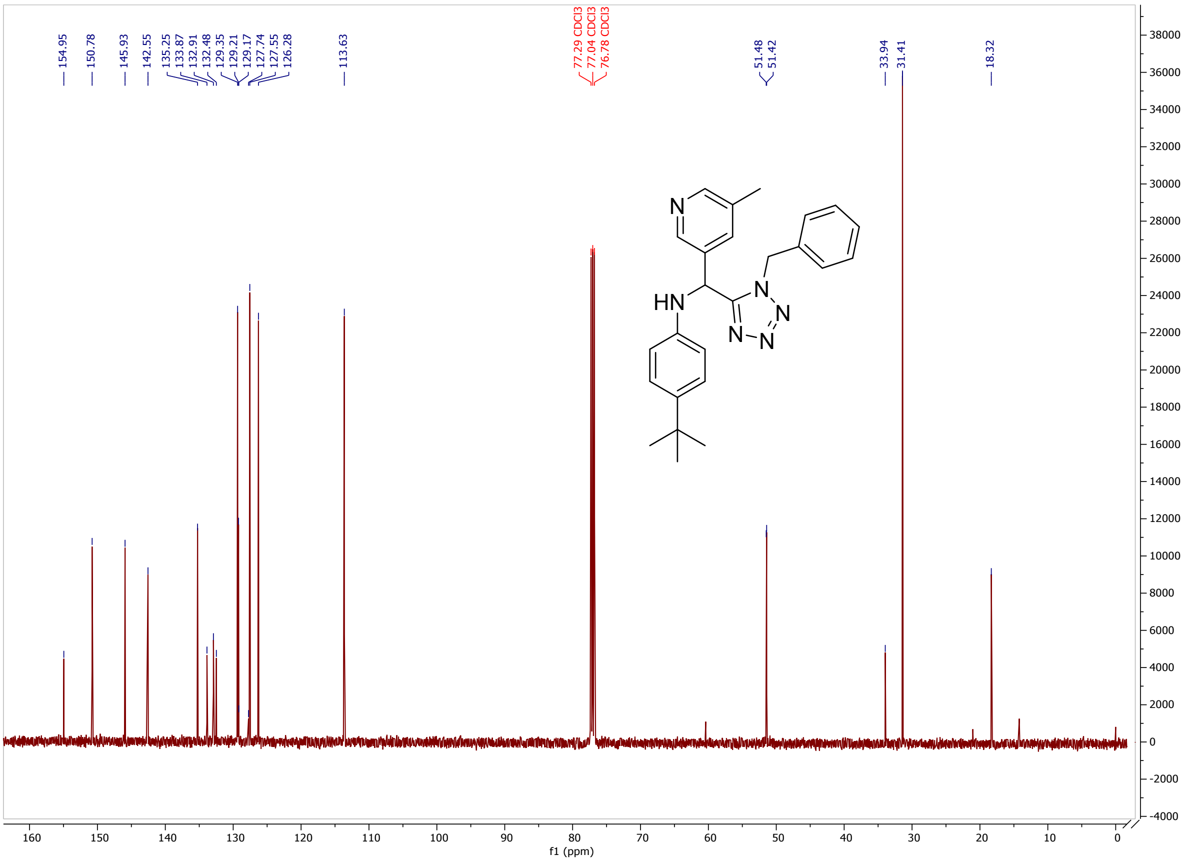

N-((1-benzyl-1H-tetrazol-5-yl)(1H-pyrazol-3-yl)methyl)-[1,1'-biphenyl]-4-amine (1m)

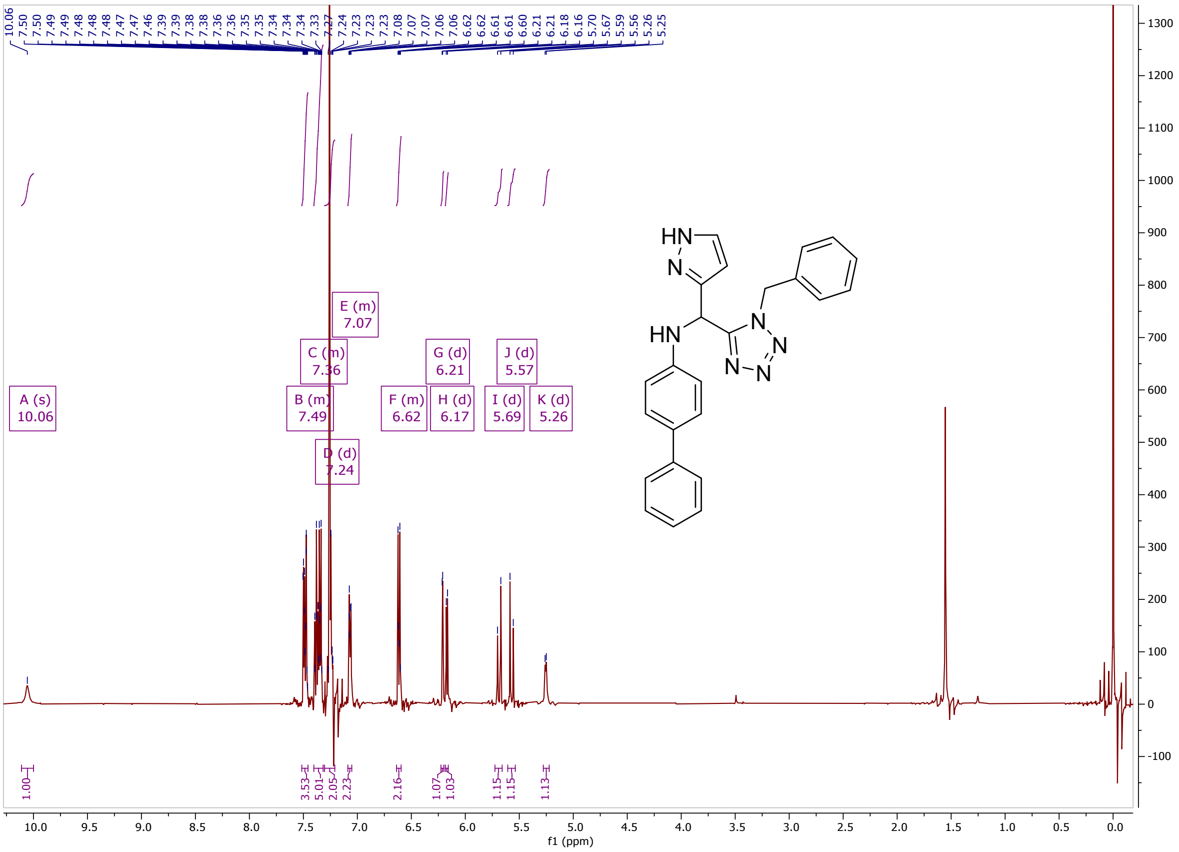

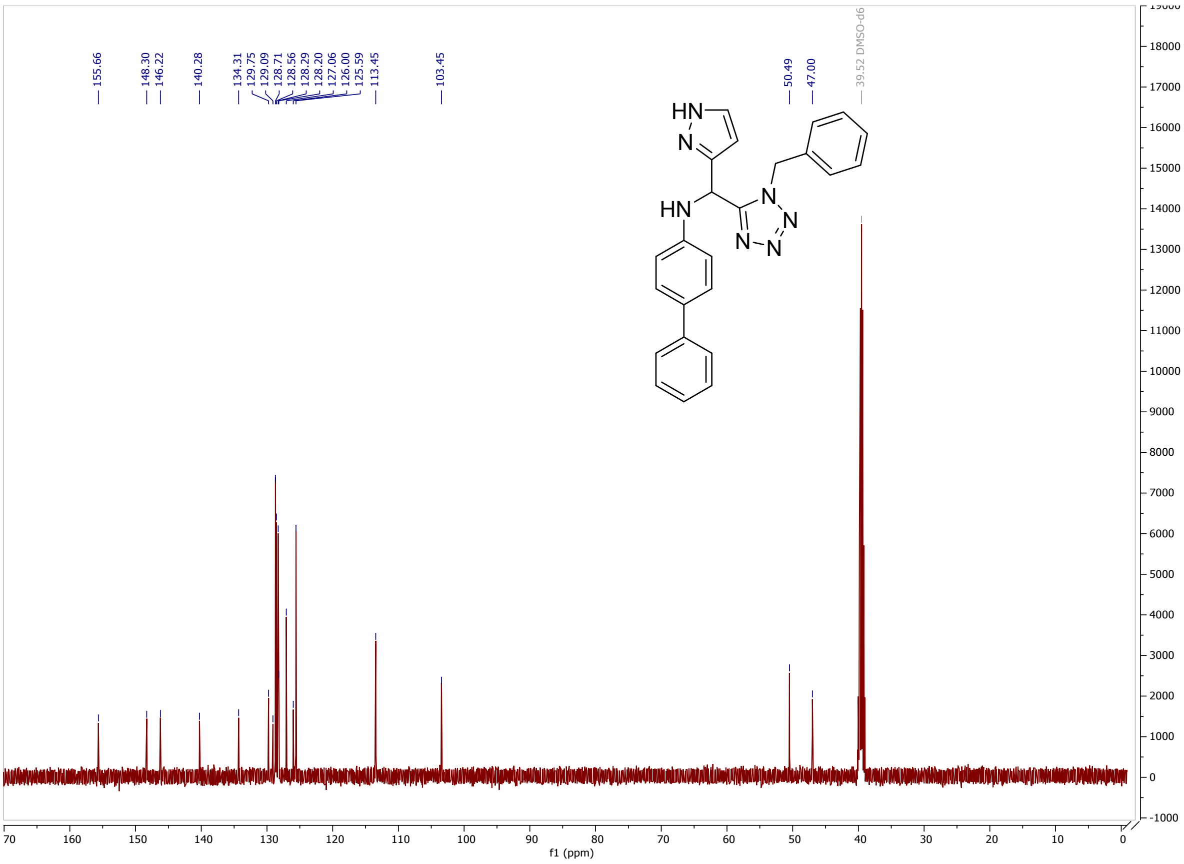

N-((1-benzyl-1H-tetrazol-5-yl)(imidazo[1,2-a]pyridin-3-yl)methyl)-[1,1'-biphenyl]-4-amine (1n)

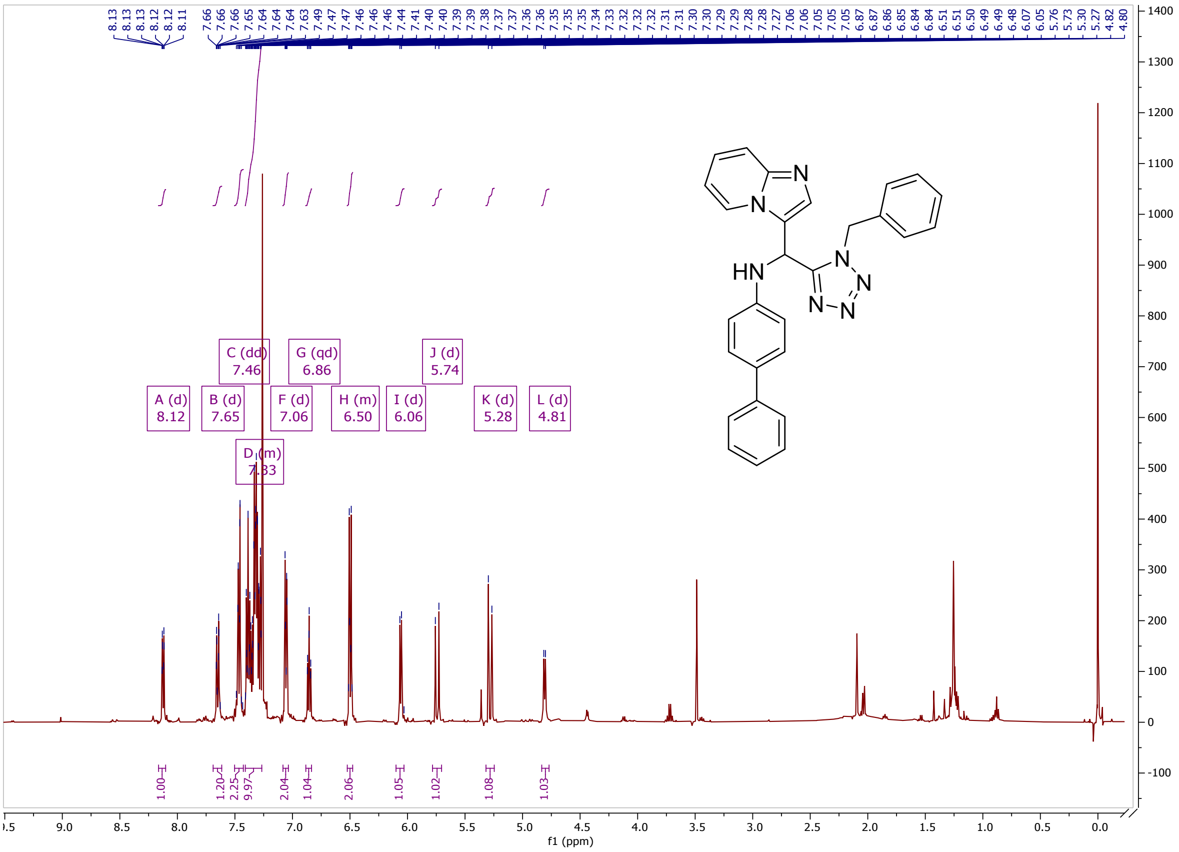

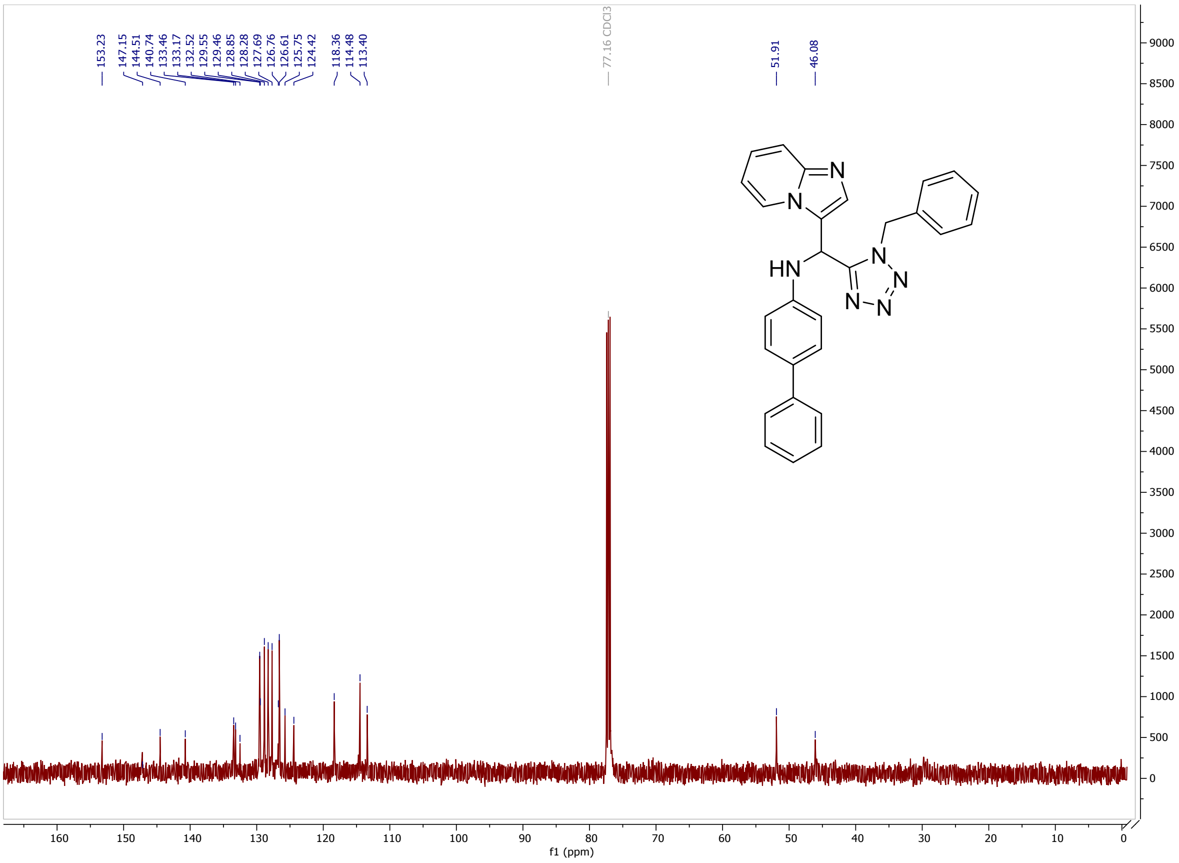

N-((1-benzyl-1H-tetrazol-5-yl)(pyridazin-3-yl)methyl)-[1,1'-biphenyl]-4-amine (1o)

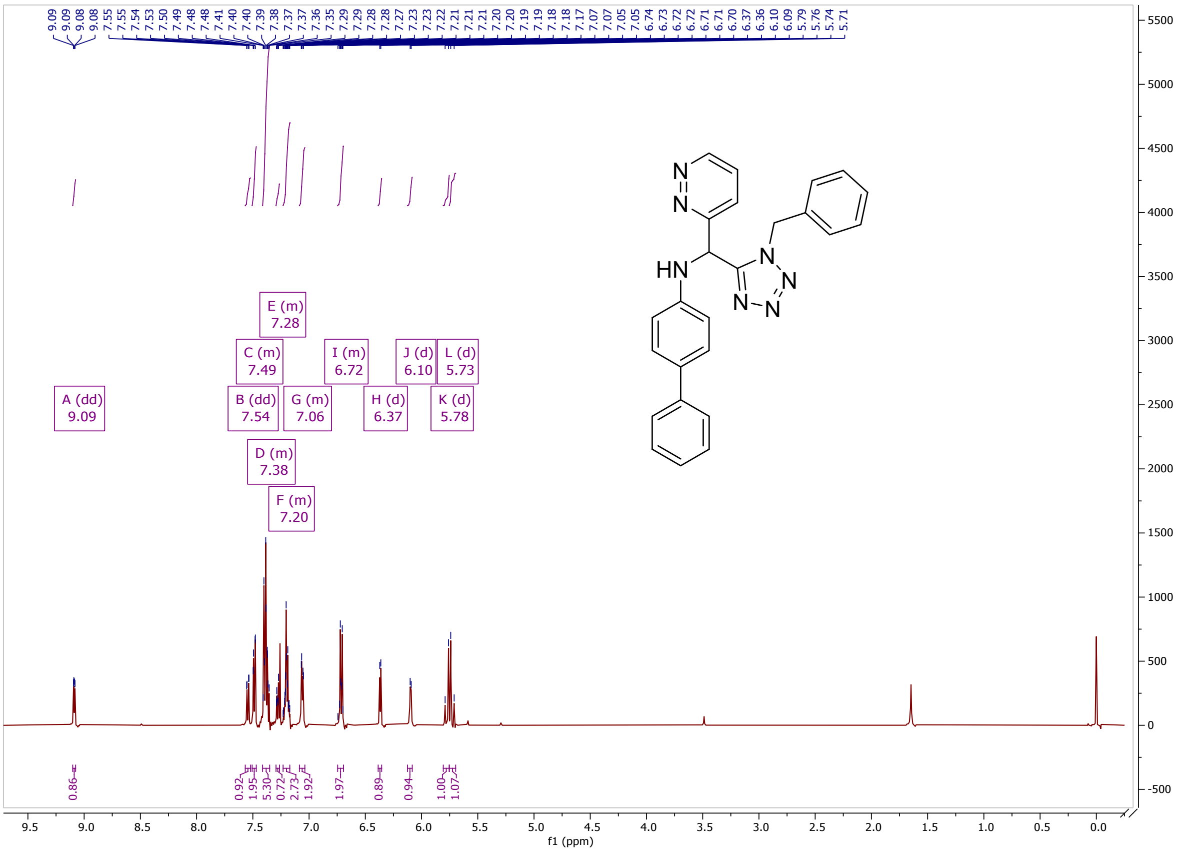

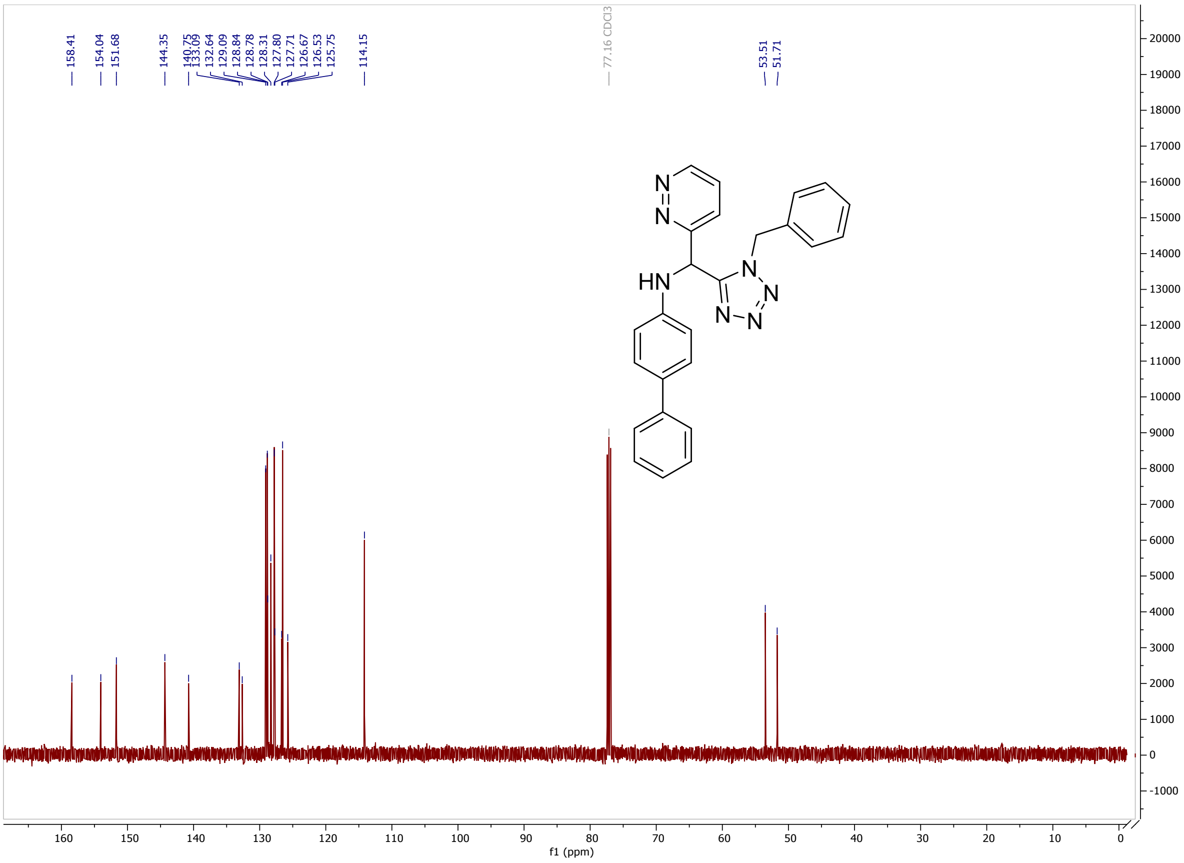

N-((1-(1-phenylcyclopropyl)-1H-tetrazol-5-yl)(pyridin-3-yl)methyl)-[1,1'-biphenyl]-4-amine (1p)

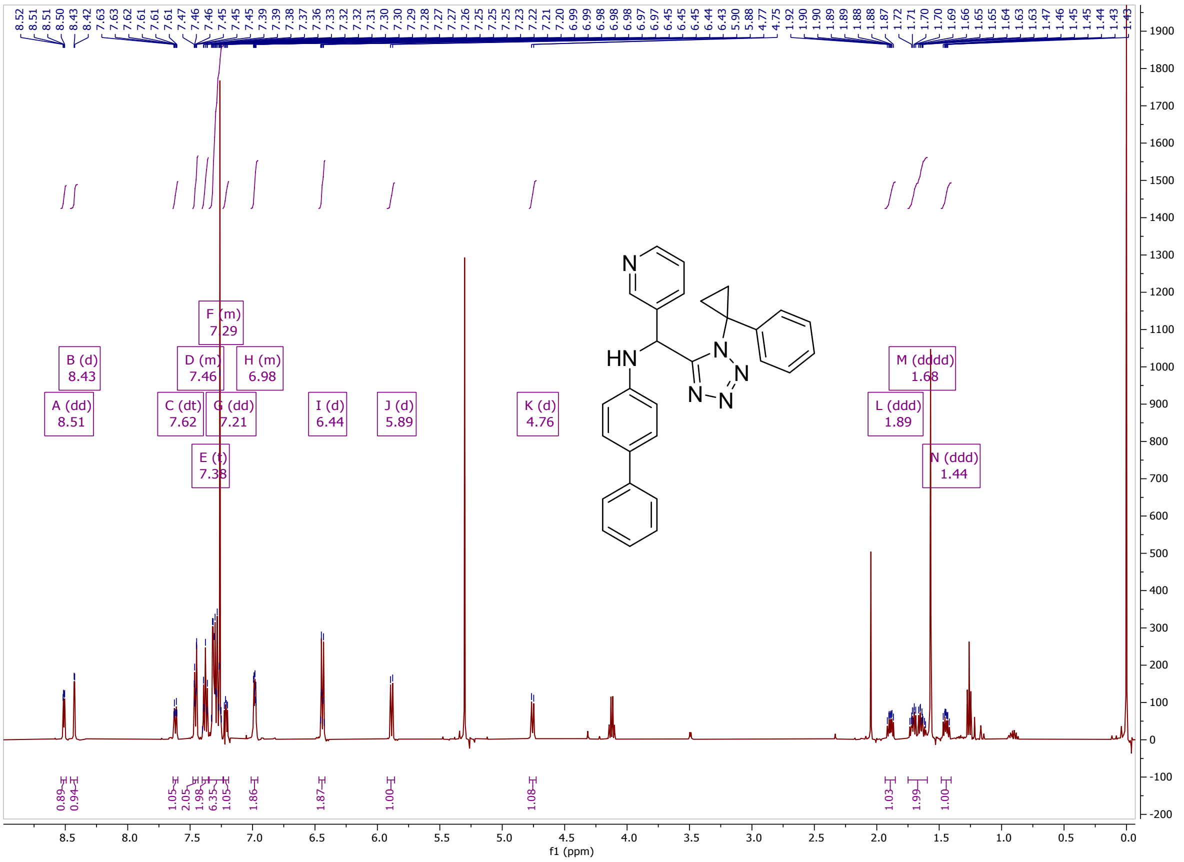

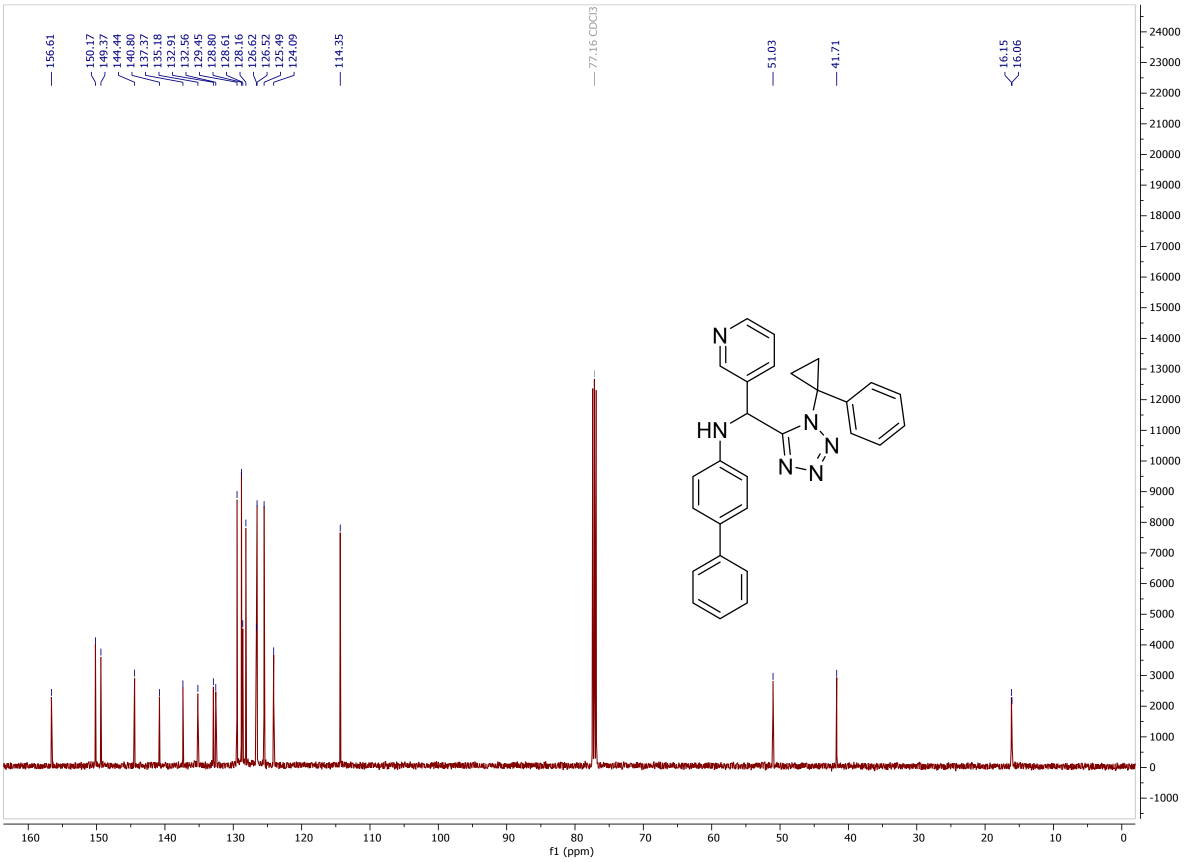

N-((1-((1-methylpiperidin-4-yl)methyl)-1H-tetrazol-5-yl)(pyridin-3-yl)methyl)-[1,1'-biphenyl]-4-amine (1q)

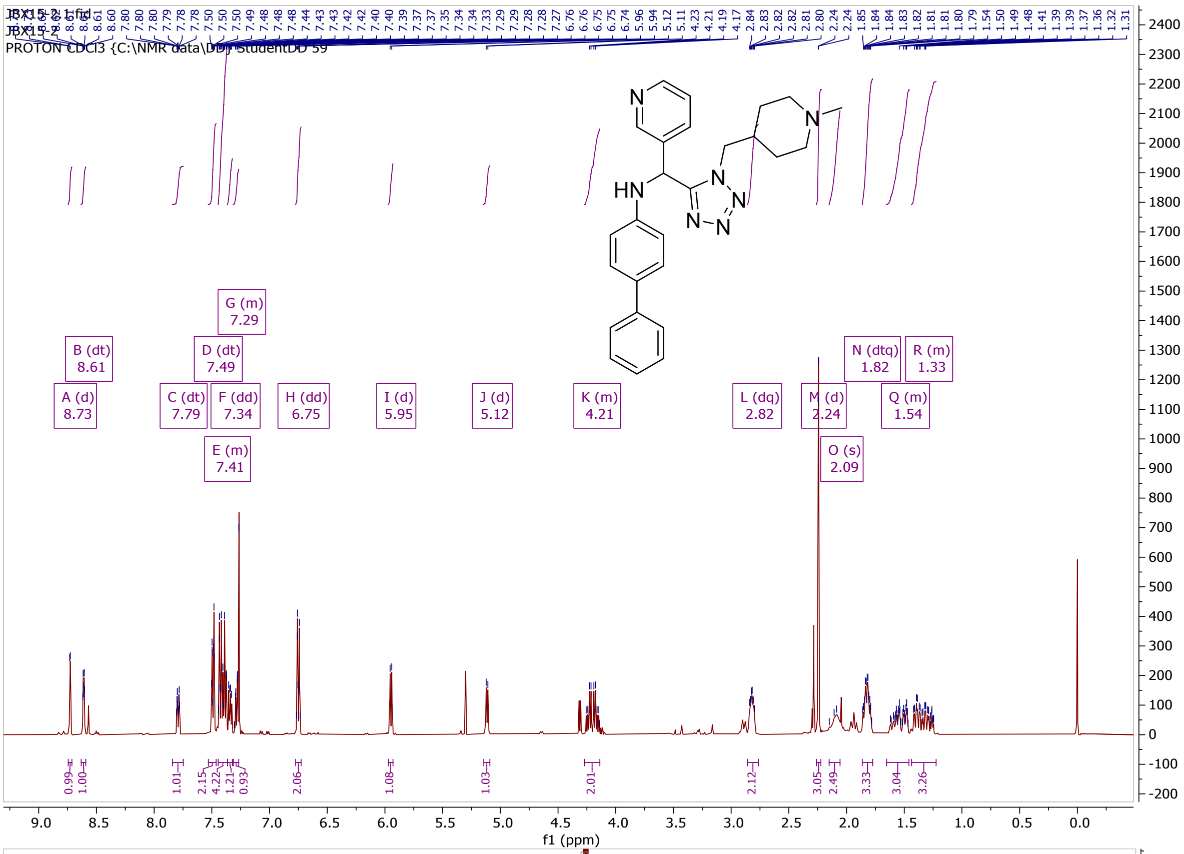

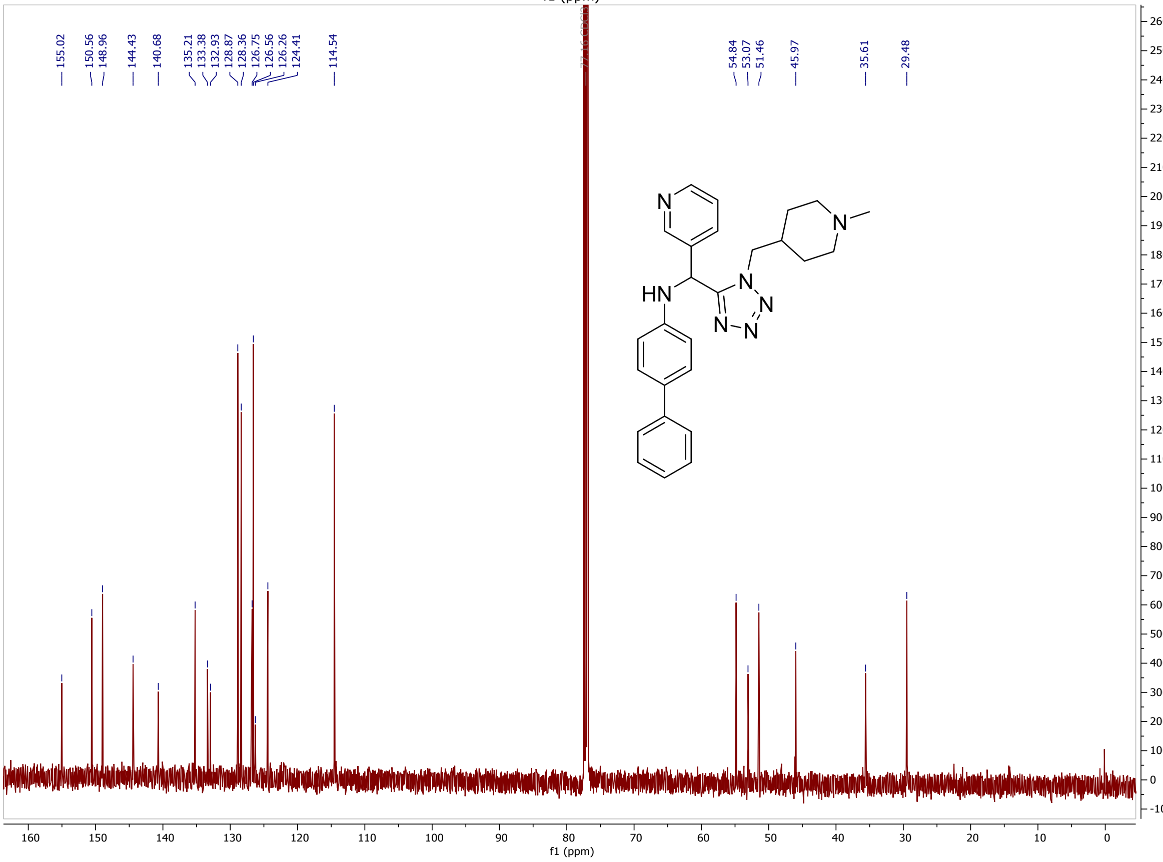

4-(5-(([1,1'-biphenyl]-4-ylamino)(pyridin-3-yl)methyl)-1H-tetrazol-1-yl)cyclohexan-1-ol (1r)

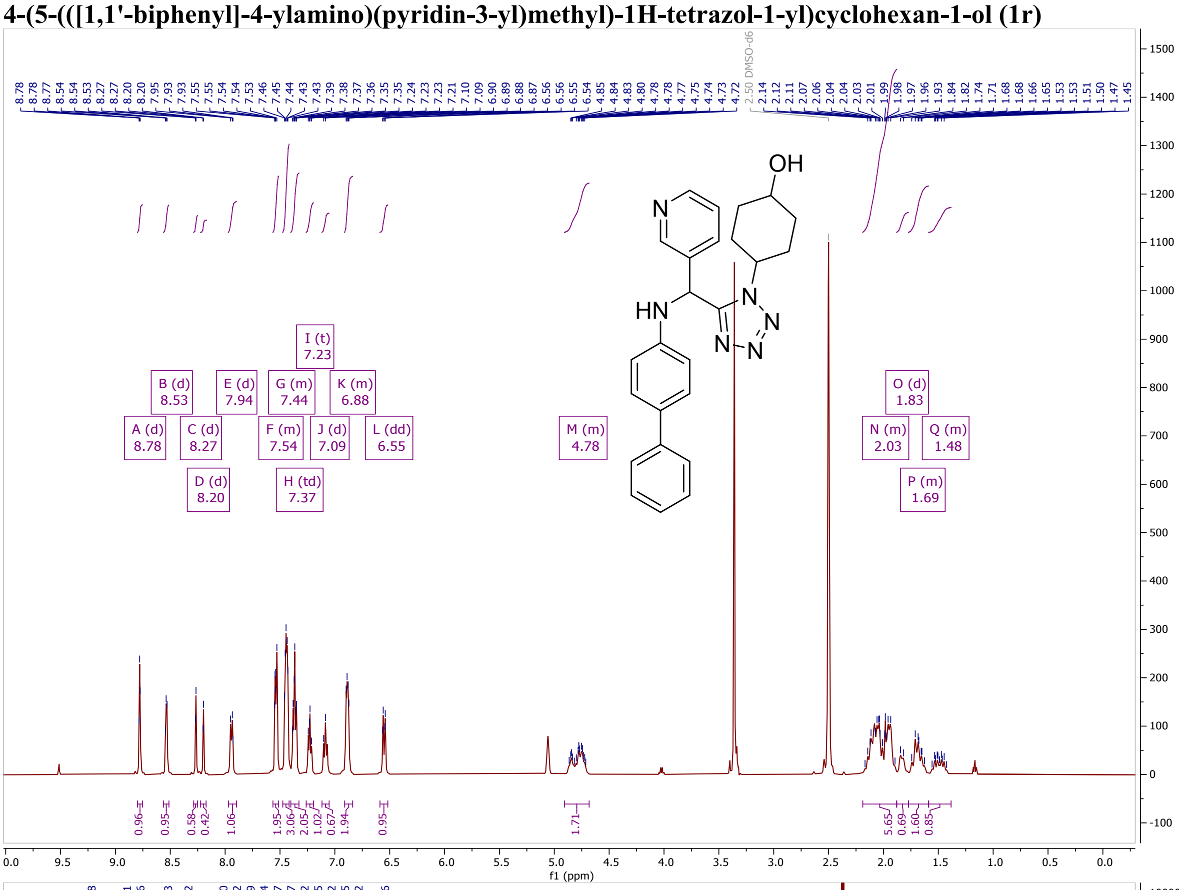

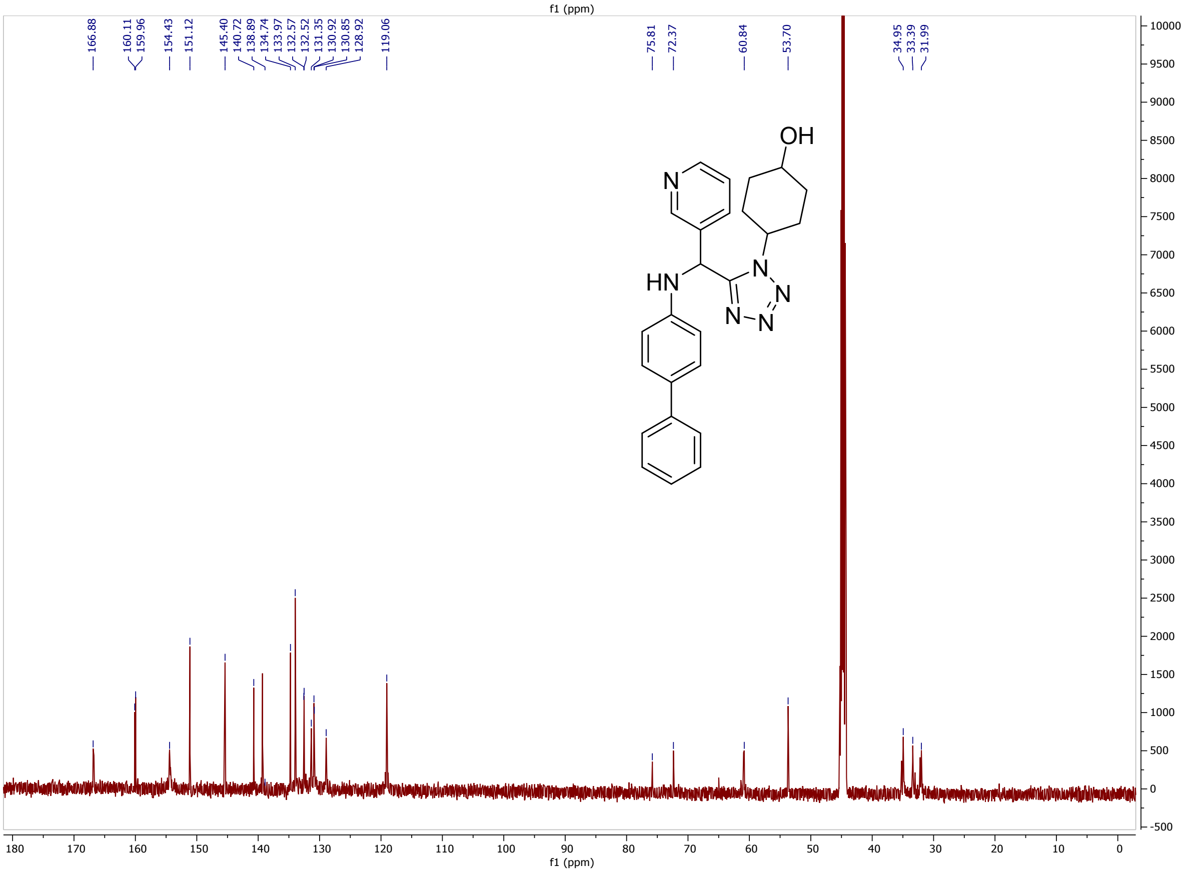

4-(tert-butyl)-N-((1-(3-fluorobenzyl)-1H-tetrazol-5-yl)(pyridin-3-yl)methyl)aniline (1s)

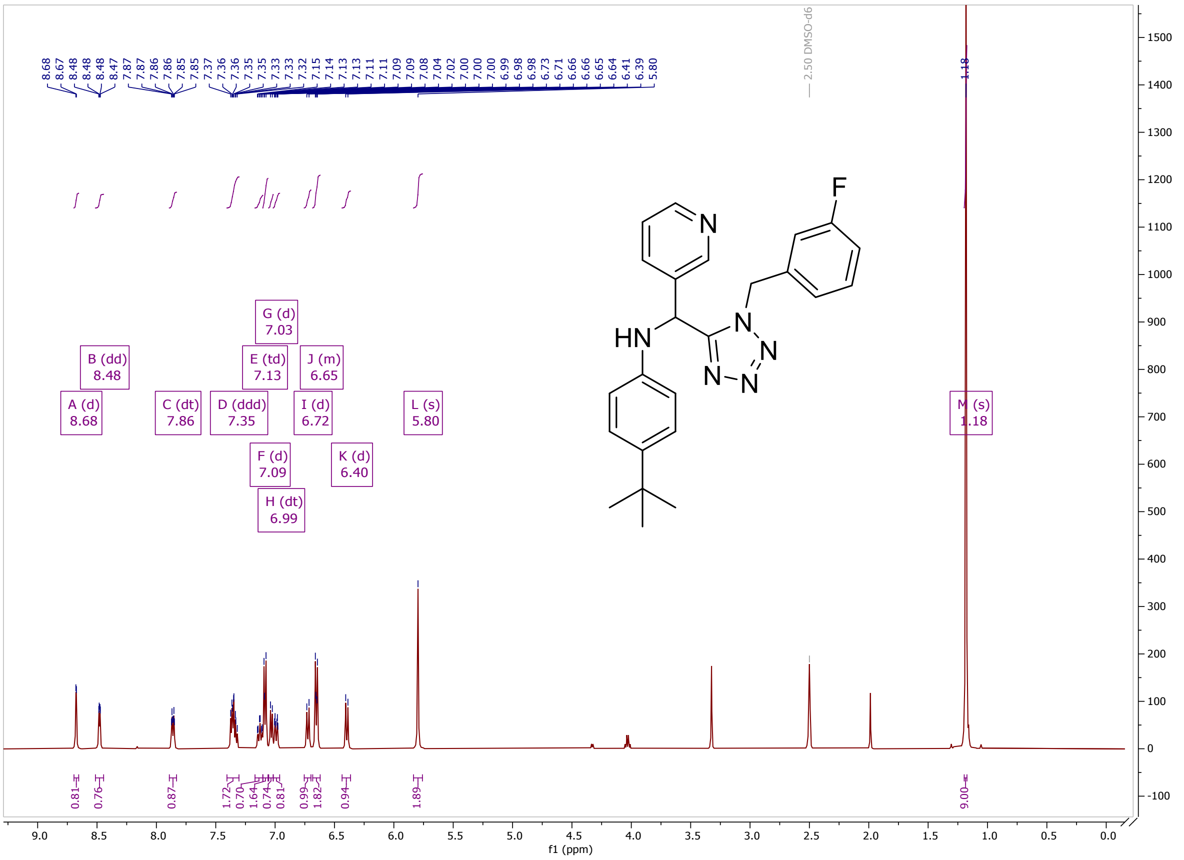

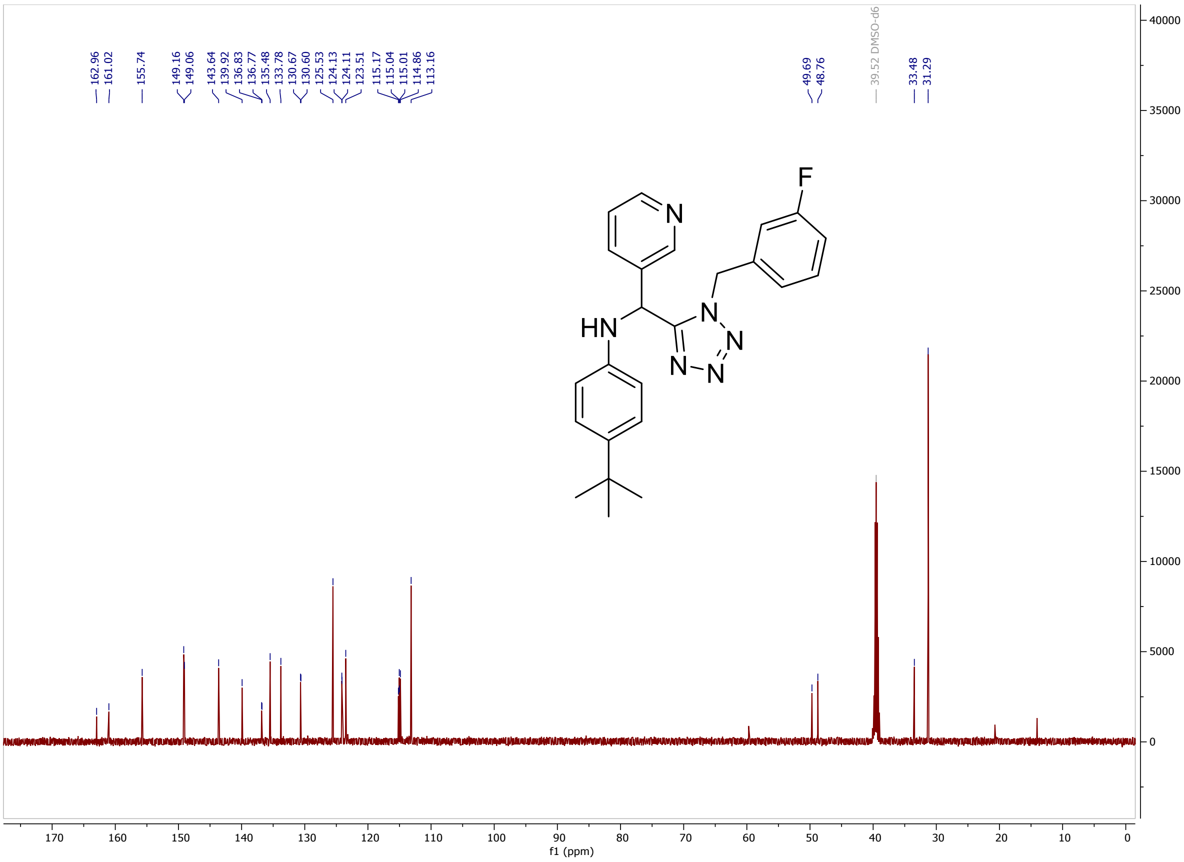

4-(tert-butyl)-N-((1-(3-fluorophenethyl)-1H-tetrazol-5-yl)(pyridin-3-yl)methyl)aniline (1t)

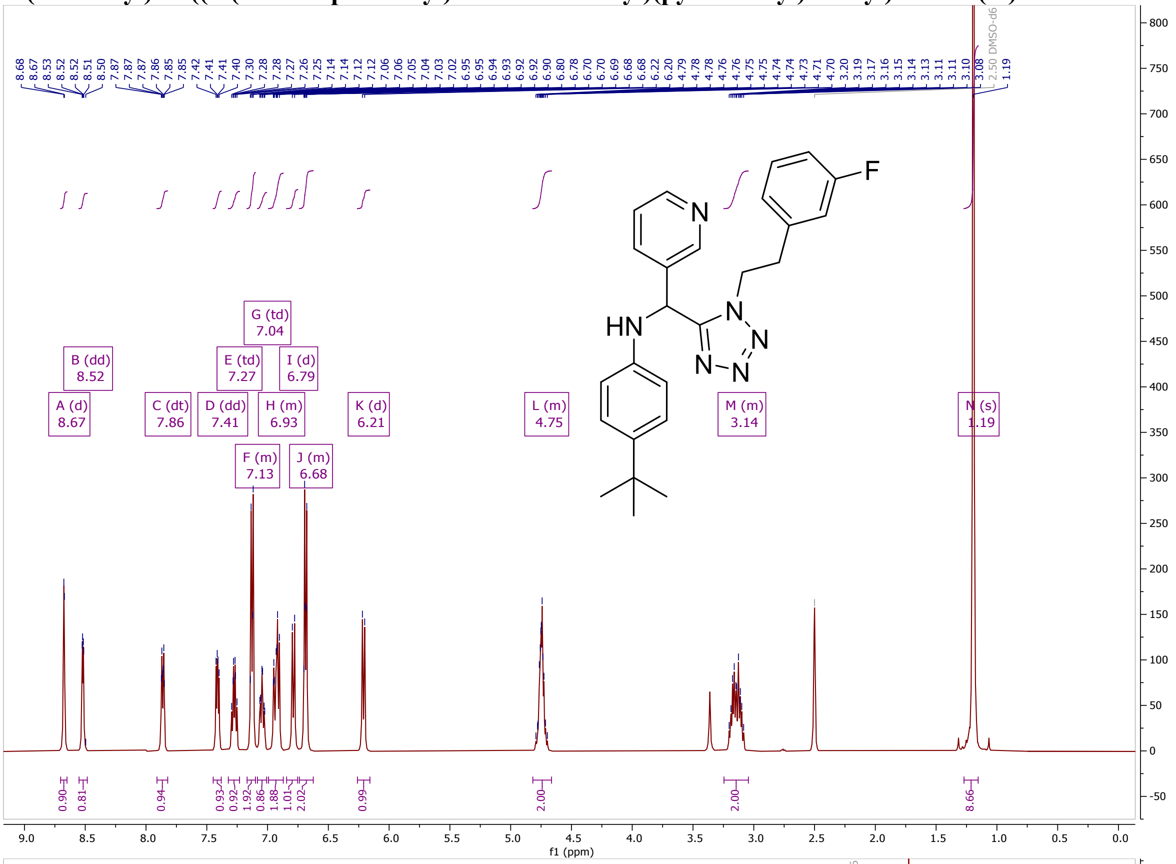

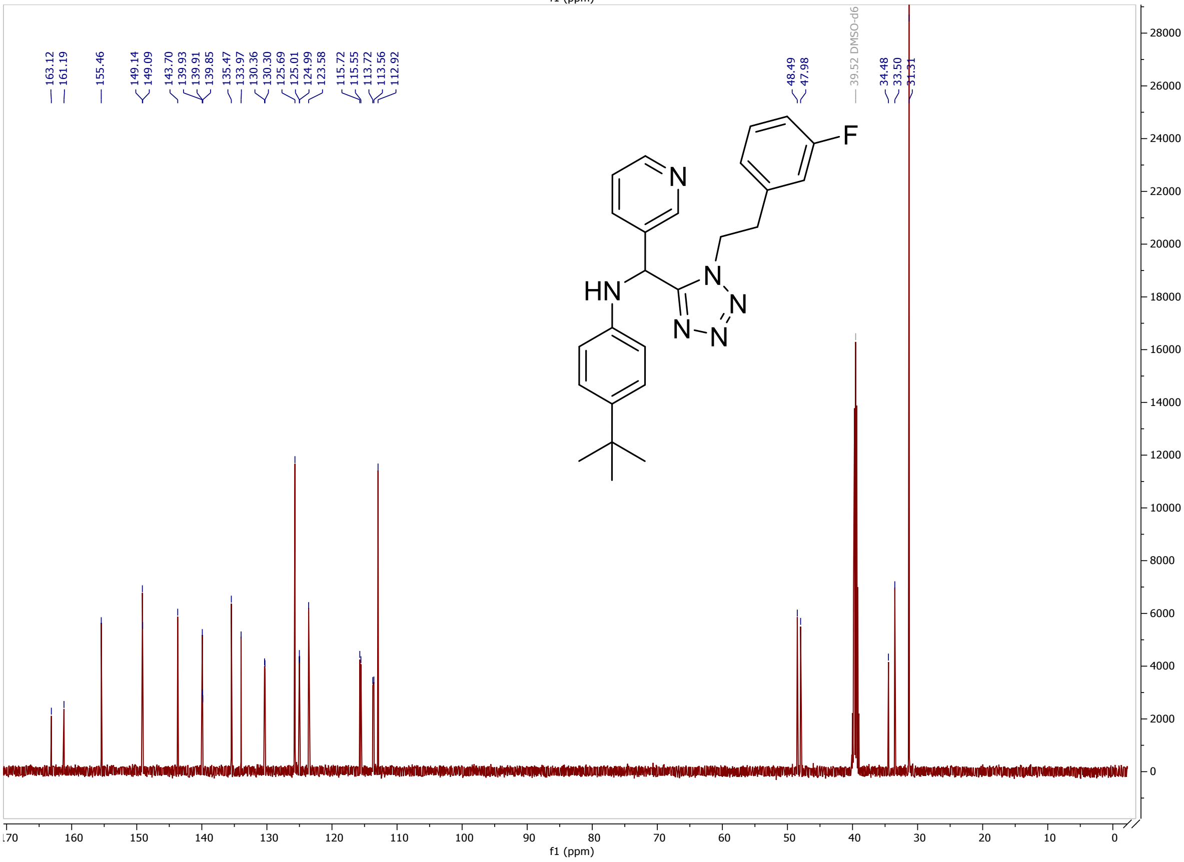

2-methyl-N-((1-(1-phenylcyclopropyl)-1H-tetrazol-5-yl)(pyridin-3-yl)methyl)-[1,1'-biphenyl]-4-amine (1u)

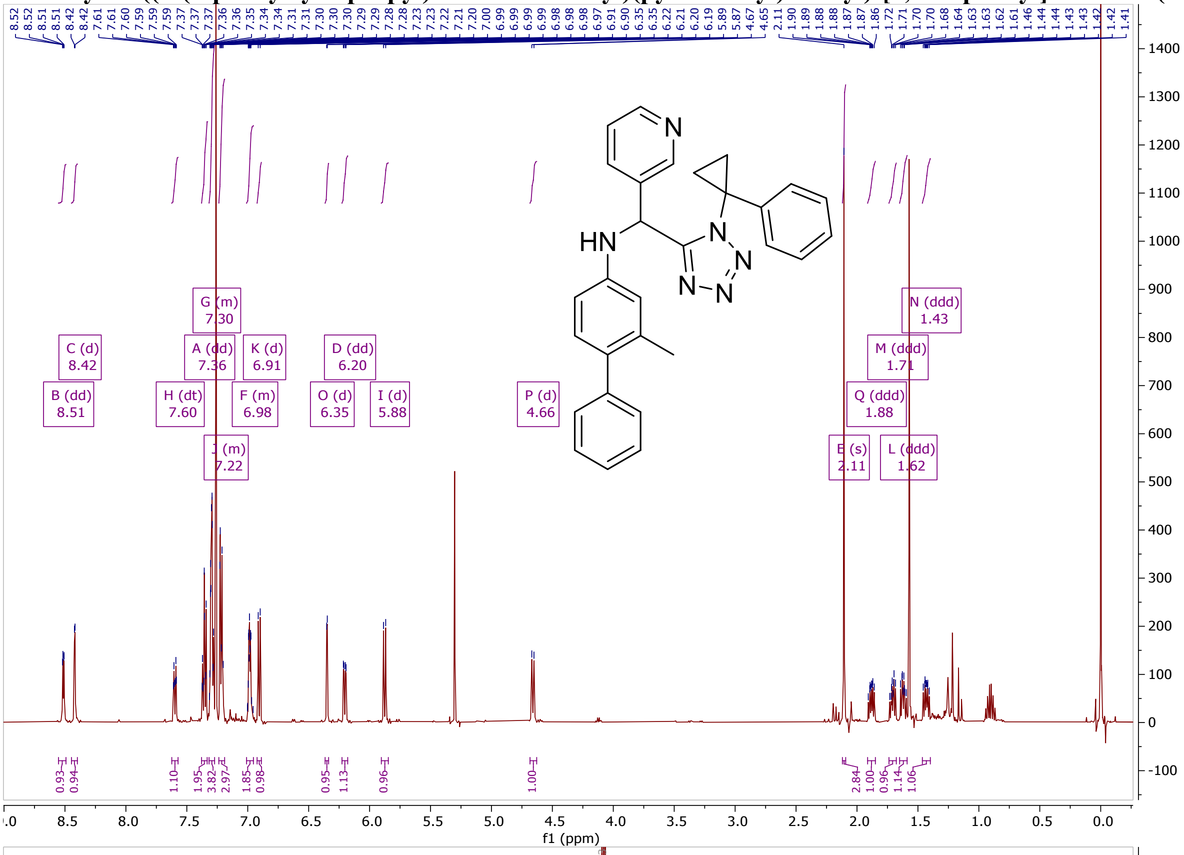

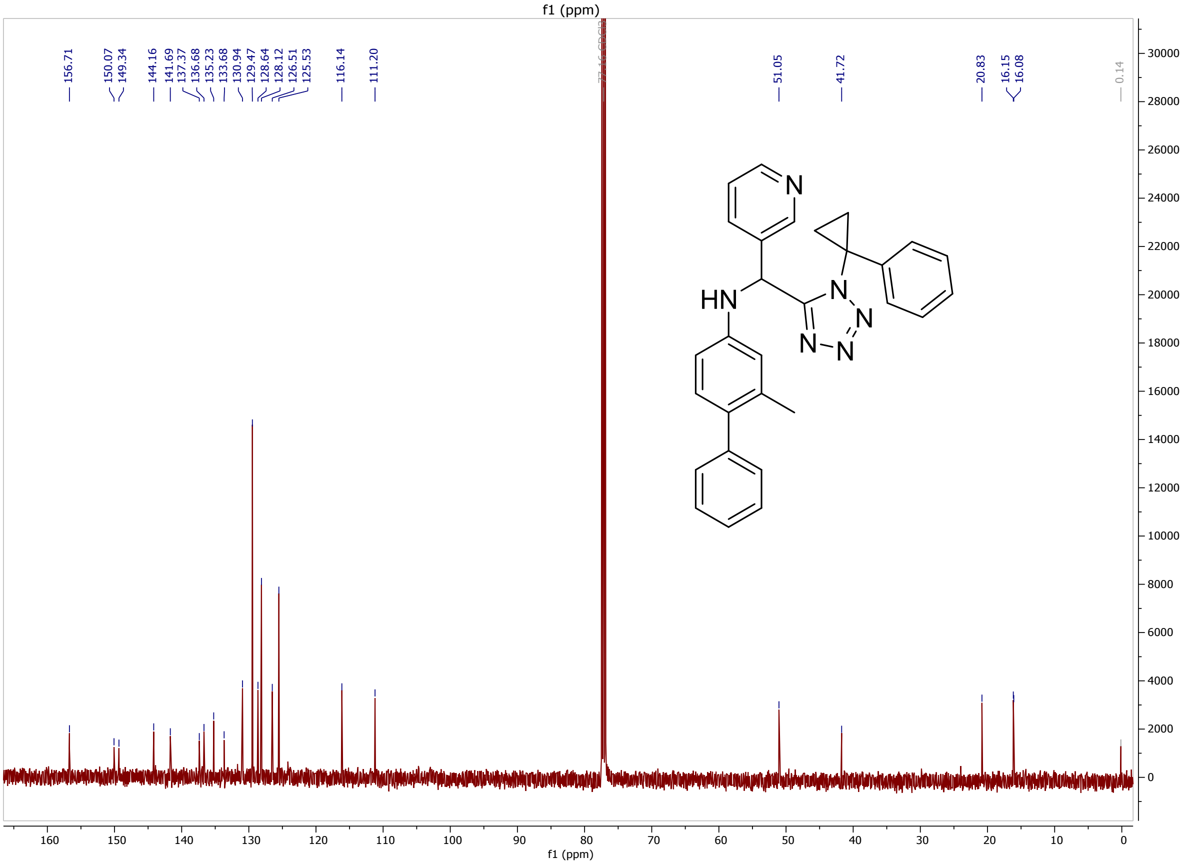

2-chloro-N-((1-(1-phenylcyclopropyl)-1H-tetrazol-5-yl)(pyridin-3-yl)methyl)-[1,1'-biphenyl]-4-amine (1v)

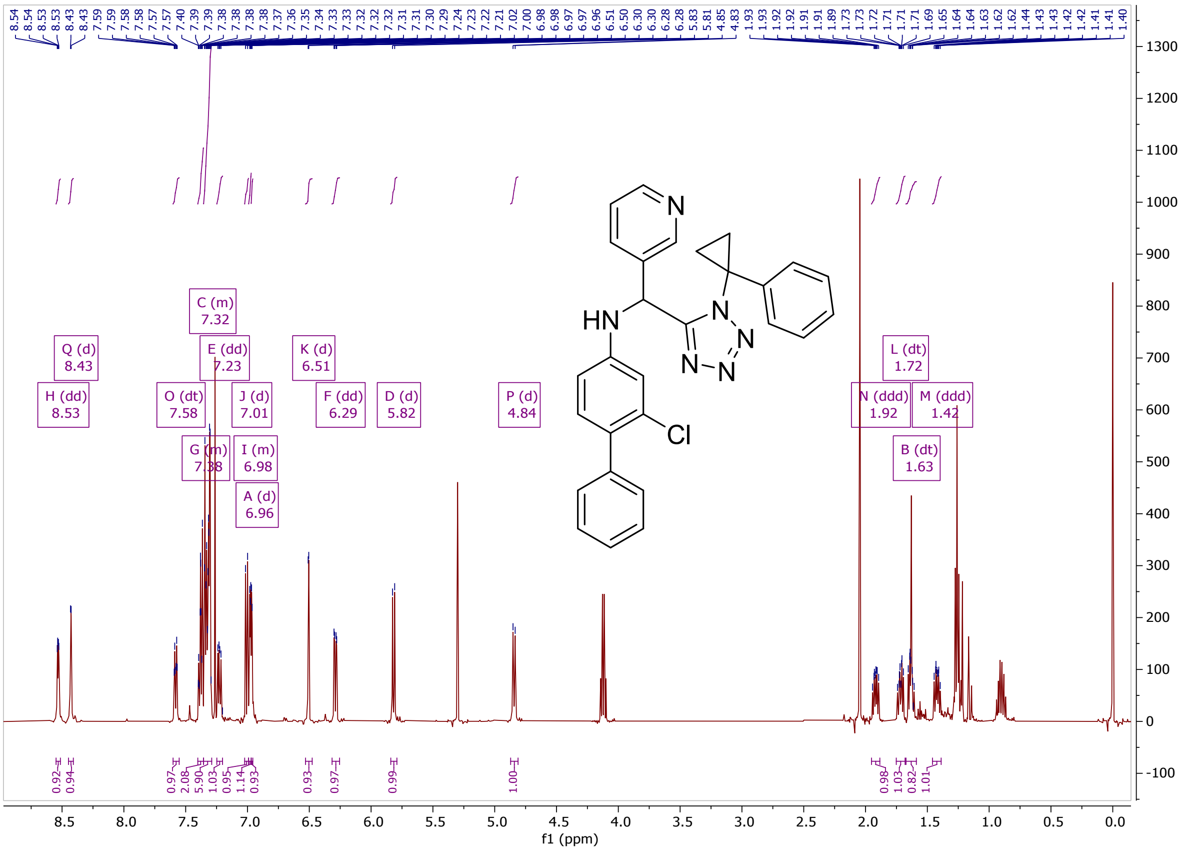

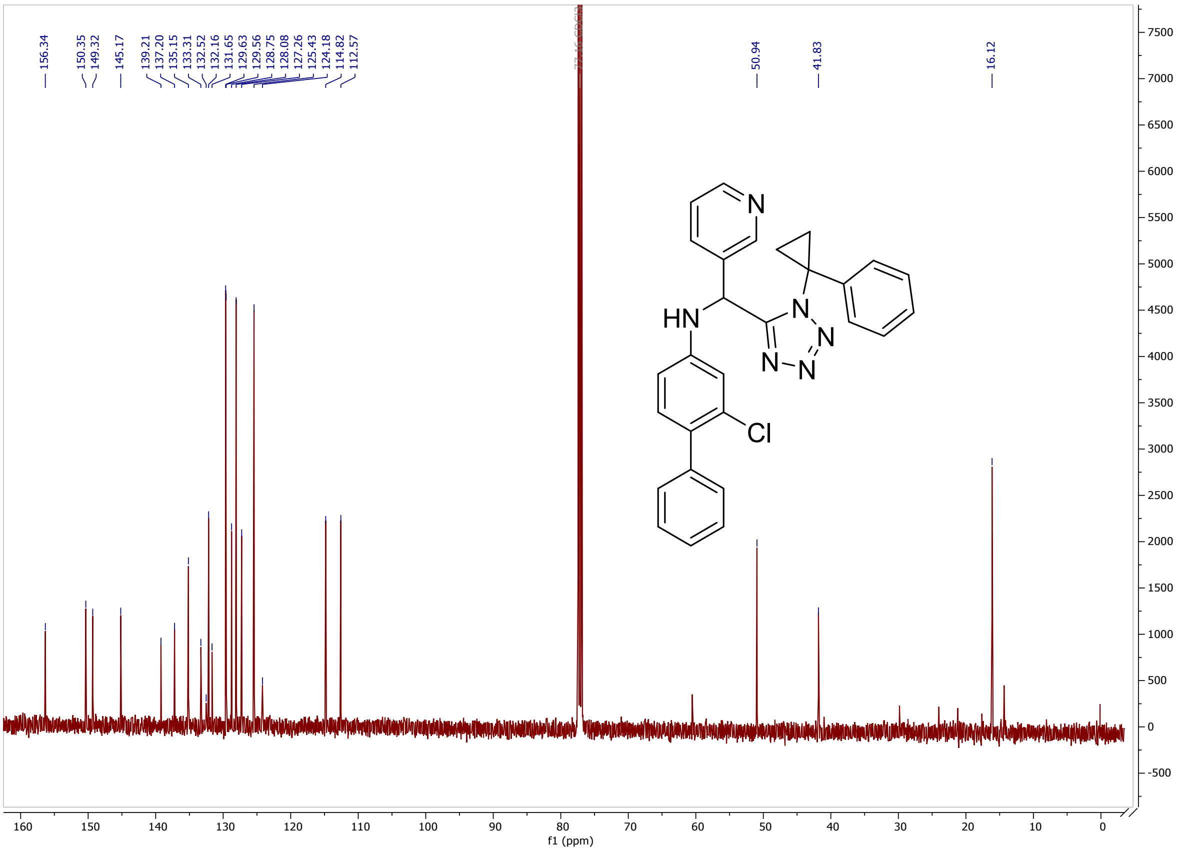

N-((1-(1-phenylcyclopropyl)-1H-tetrazol-5-yl)(pyridin-3-yl)methyl)-2-(trifluoromethyl)-[1,1'-biphenyl]-4-amine (1w)

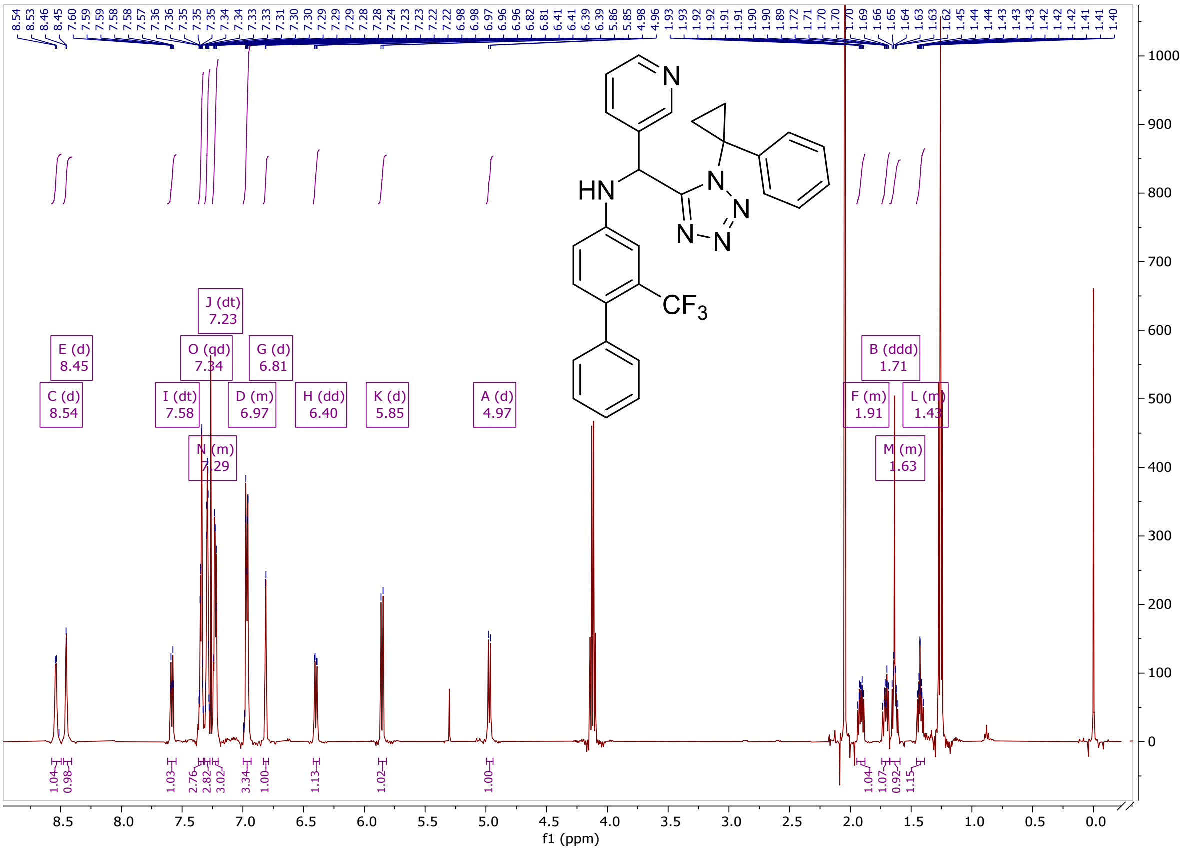

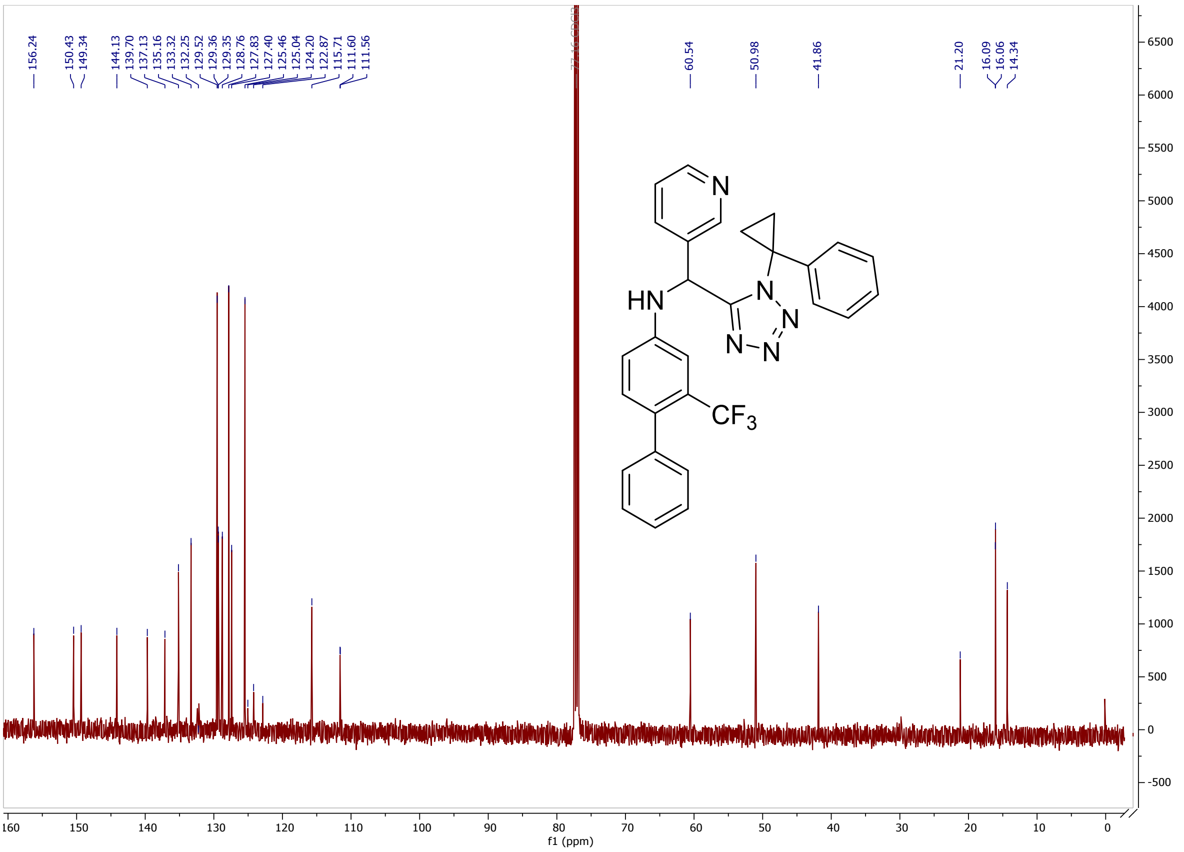

2'-methyl-N-((1-(1-phenylcyclopropyl)-1H-tetrazol-5-yl)(pyridin-3-yl)methyl)-[1,1'-biphenyl]-4-amine (1x)

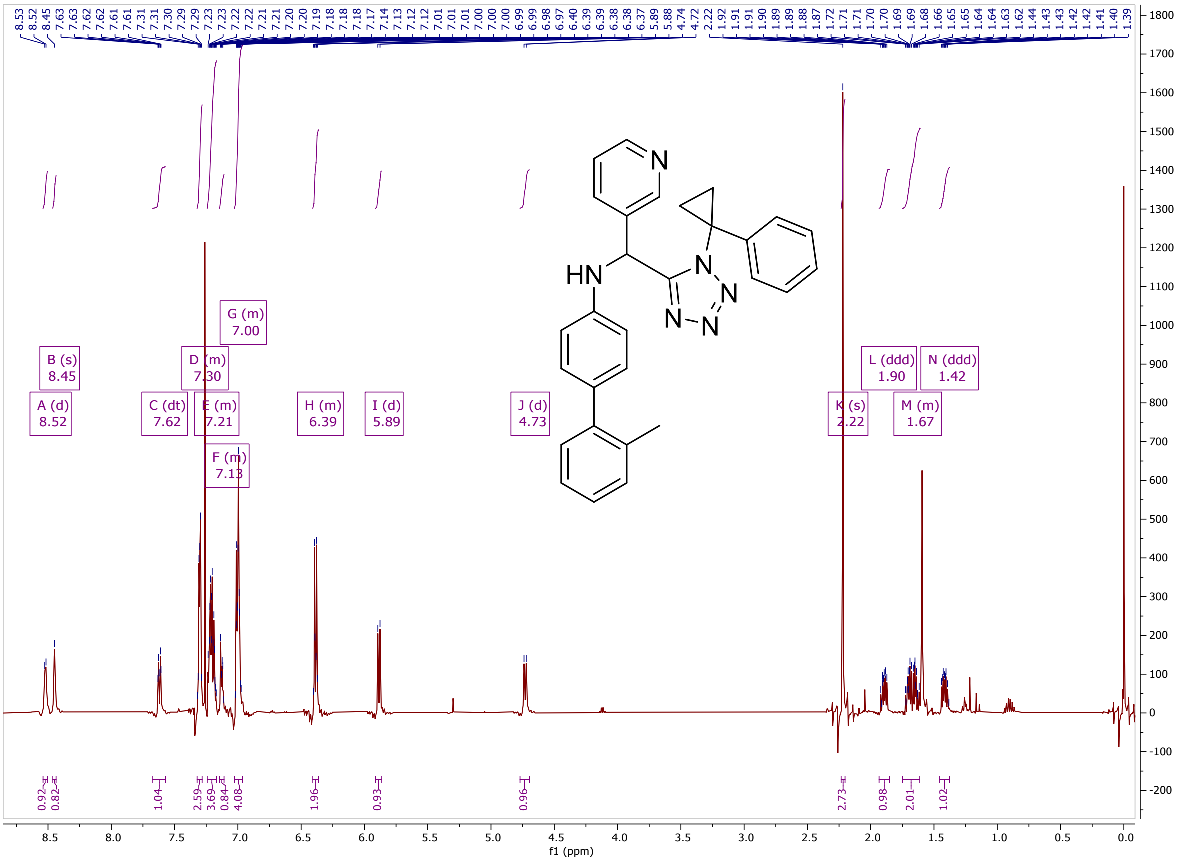

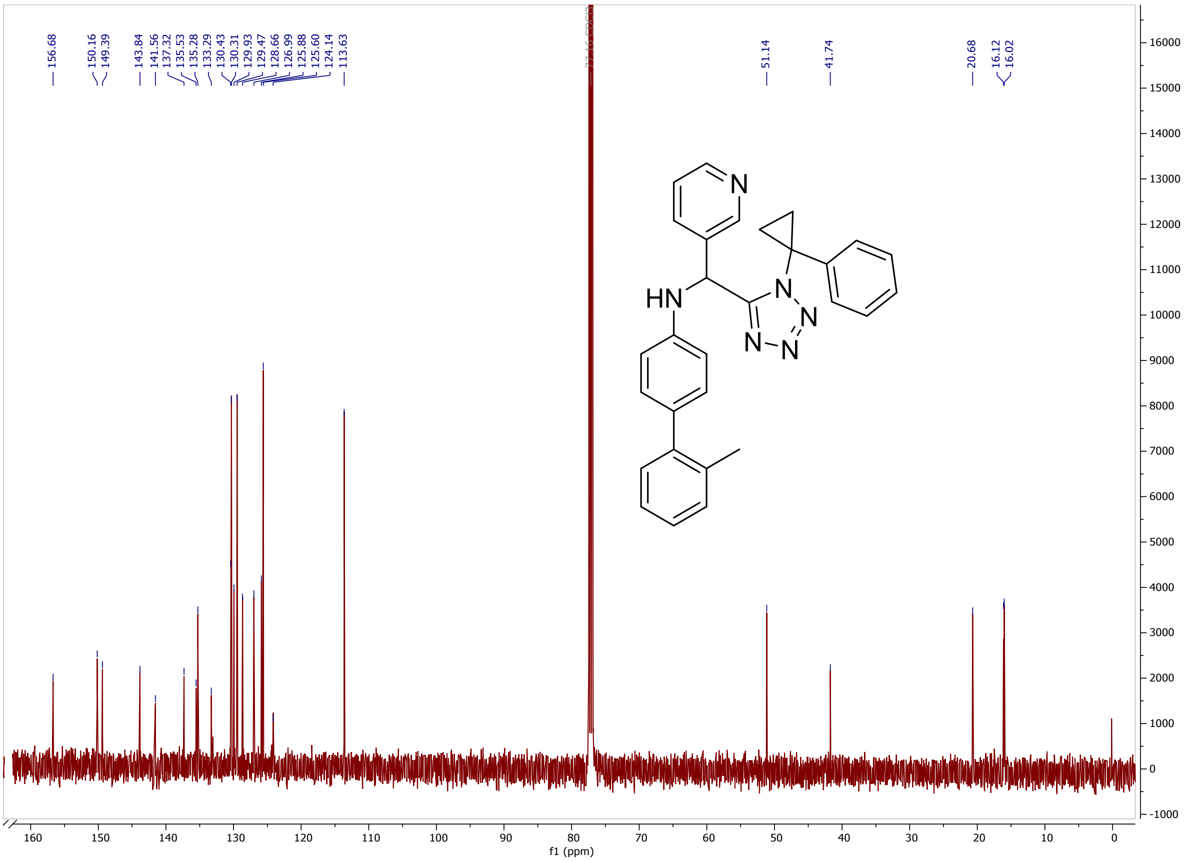

2'-methyl-N-((1-(1-phenylcyclopropyl)-1H-tetrazol-5-yl)(pyridin-3-yl)methyl)-[1,1'-biphenyl]-4-amine (1y)

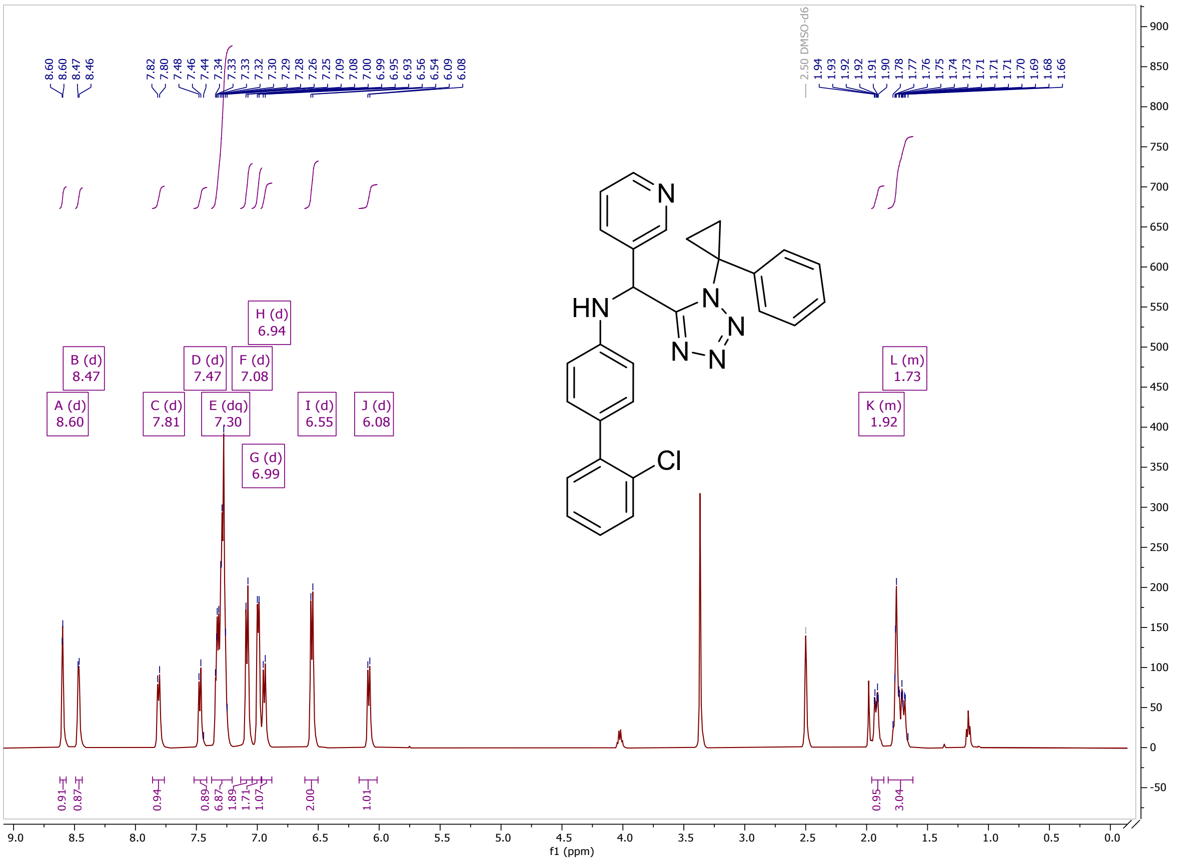

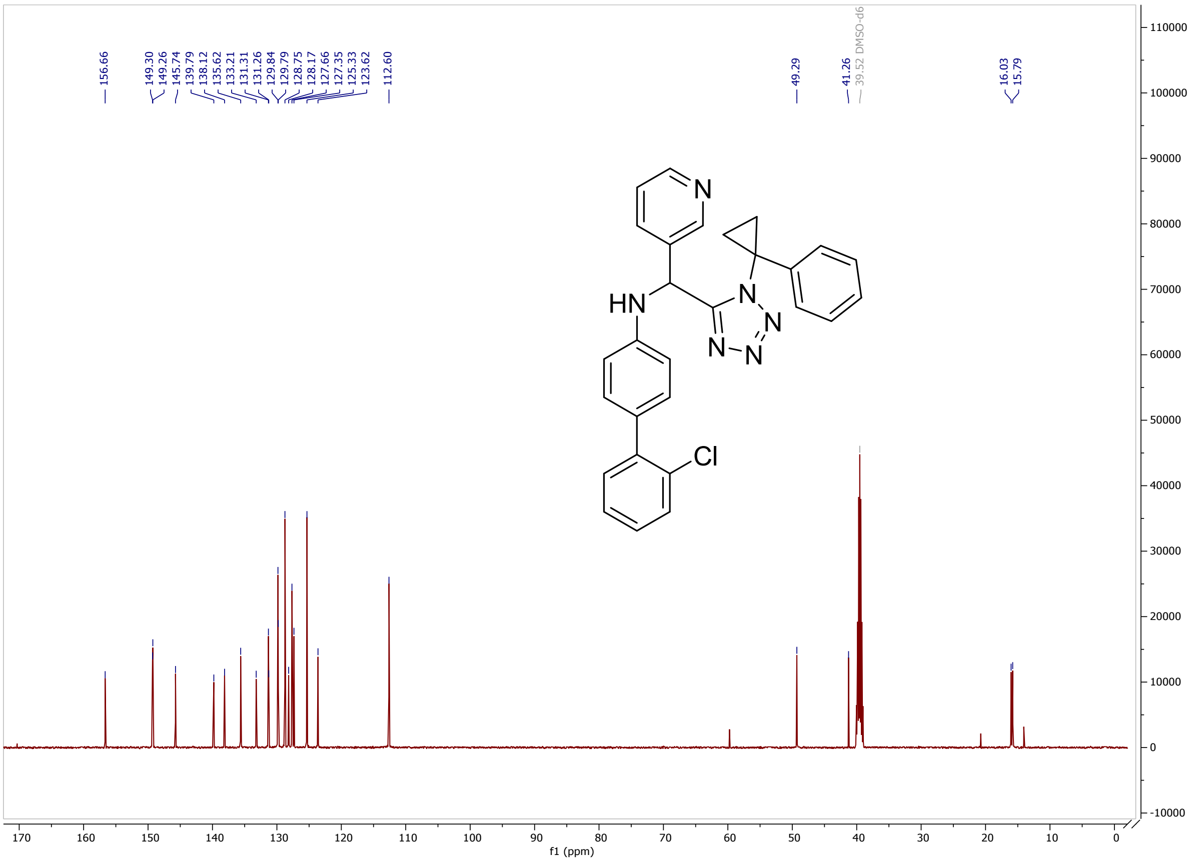

N-((1-(1-phenylcyclopropyl)-1H-tetrazol-5-yl)(pyridin-3-yl)methyl)-2'-(trifluoromethyl)-[1,1'-biphenyl]-4-amine (1z)

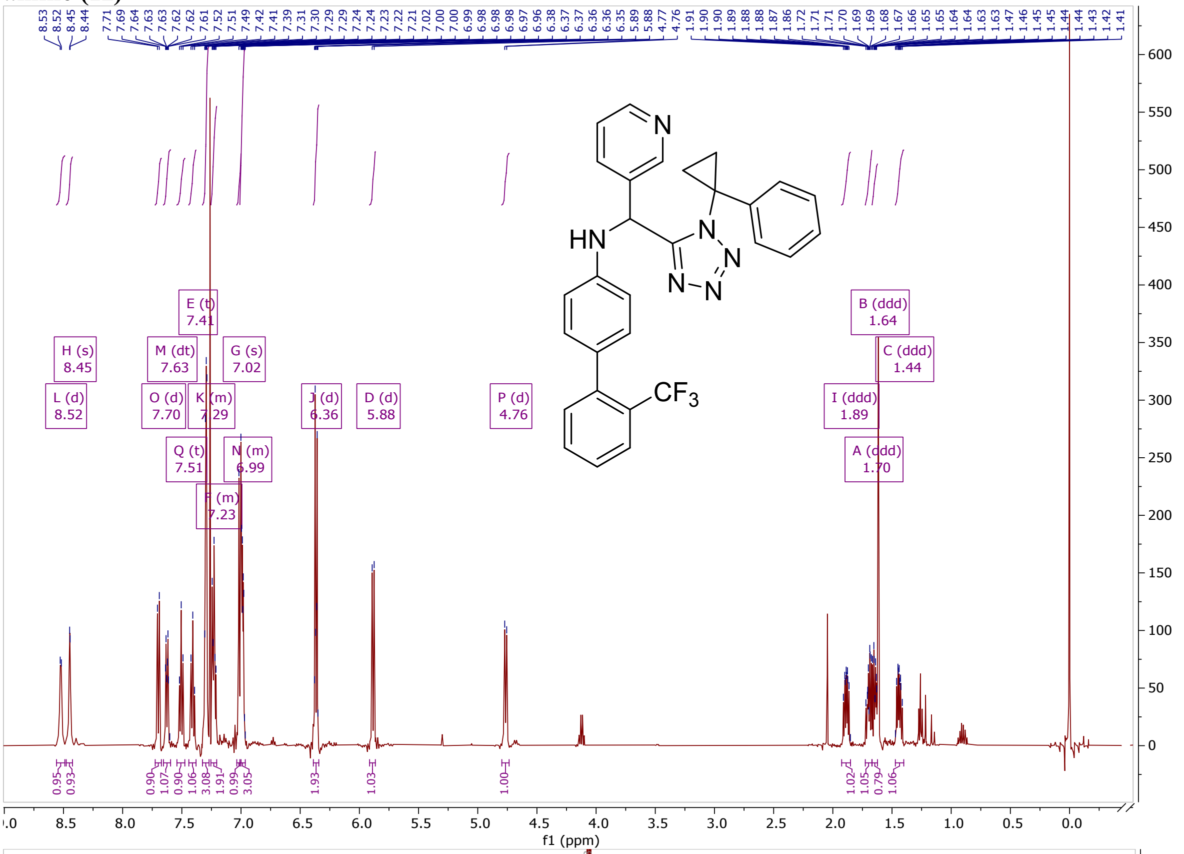

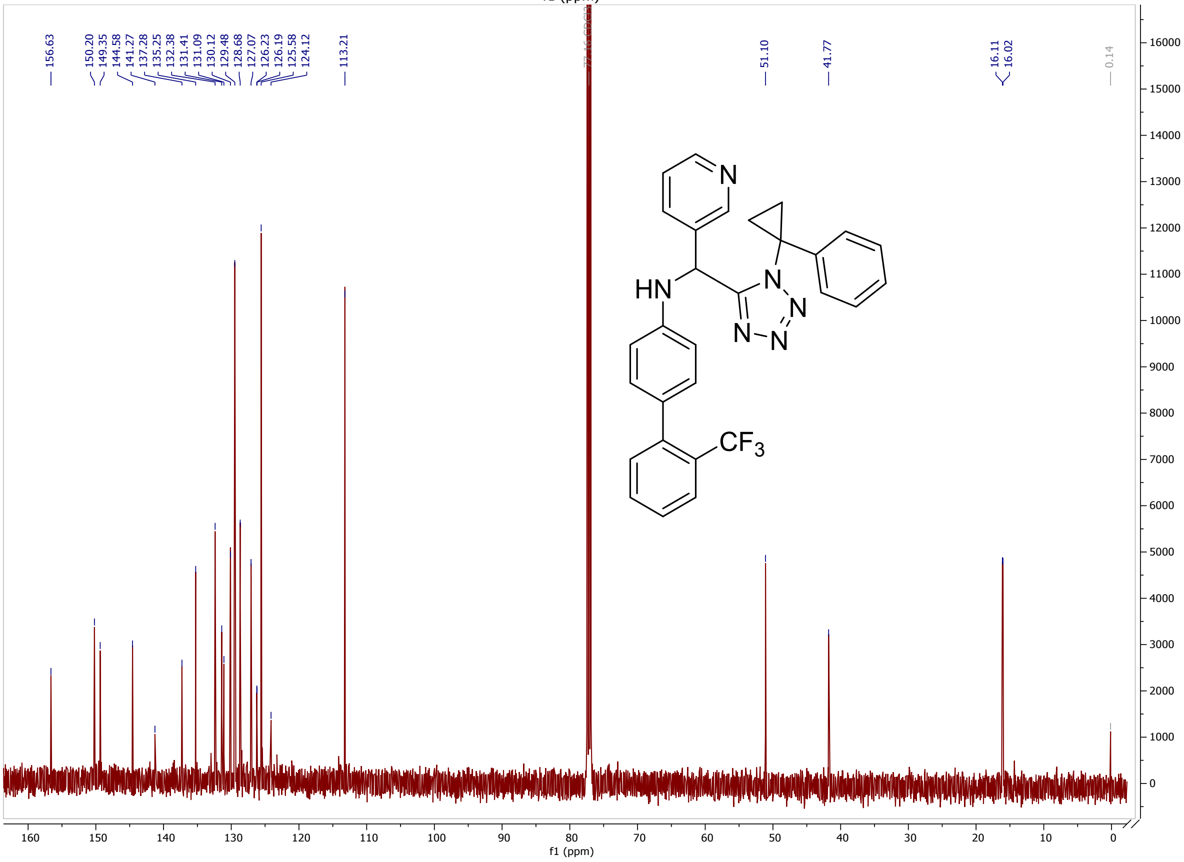

3-(([1,1'-biphenyl]-4-ylamino)(1-(3-methoxyphenyl)-1H-tetrazol-5-yl)methyl)phenol (1aa)

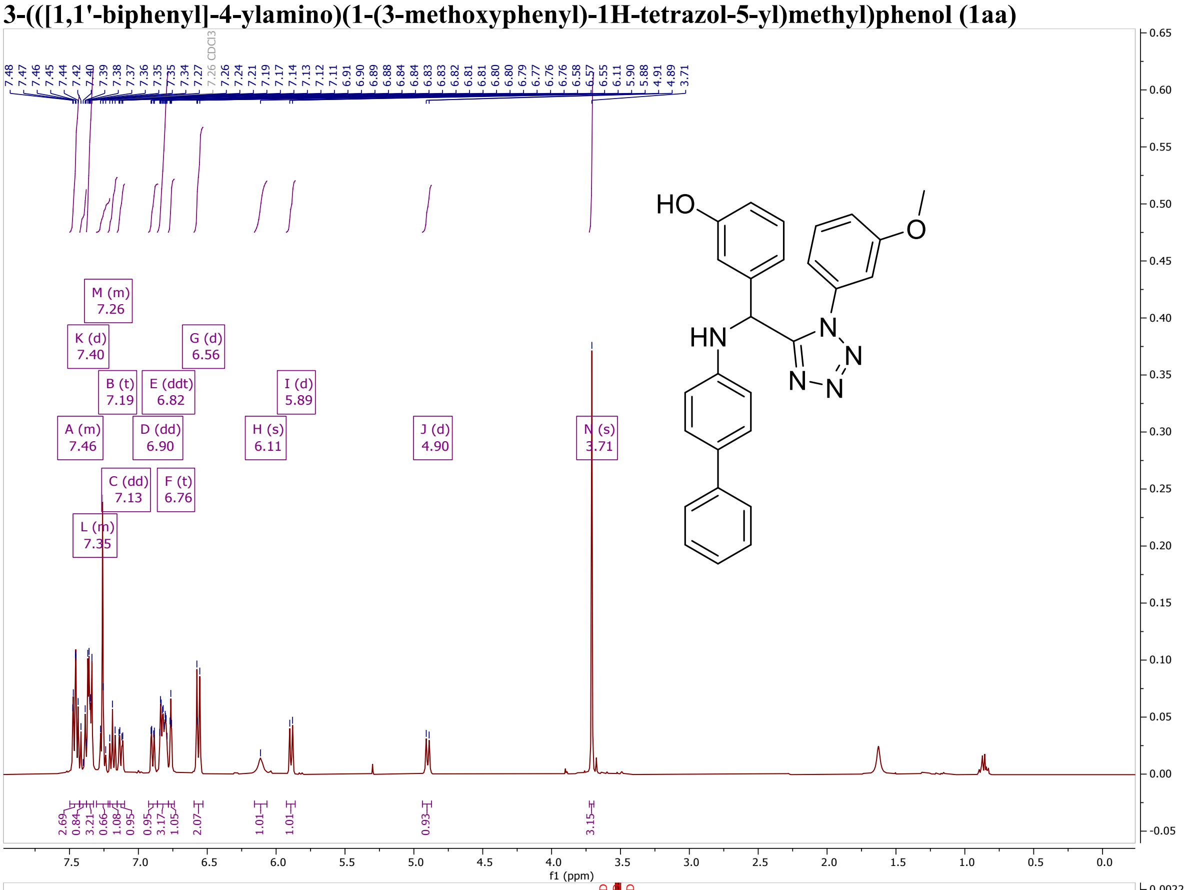

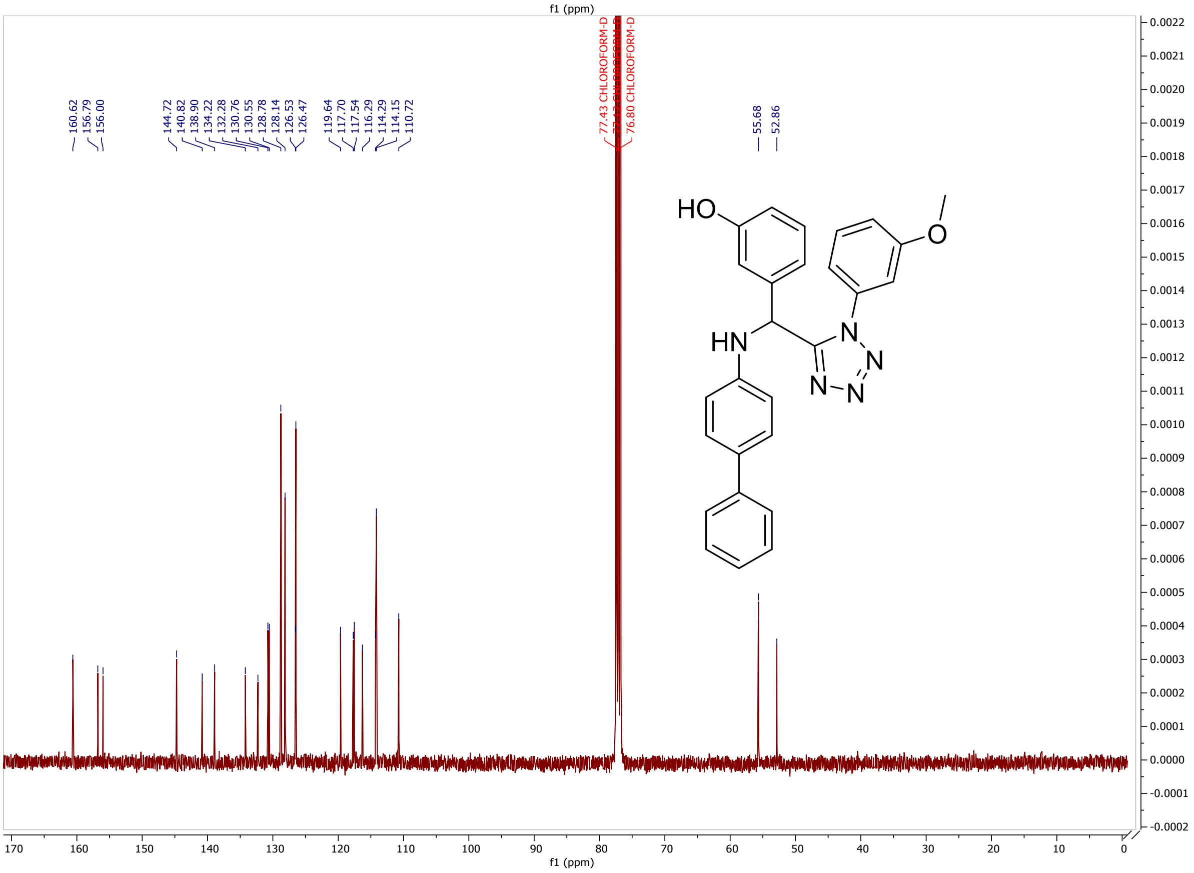

(3-(([1,1'-biphenyl]-4-ylamino)(1-(3-methoxyphenyl)-1H-tetrazol-5-yl)methyl)phenyl)boronic acid (1ab)

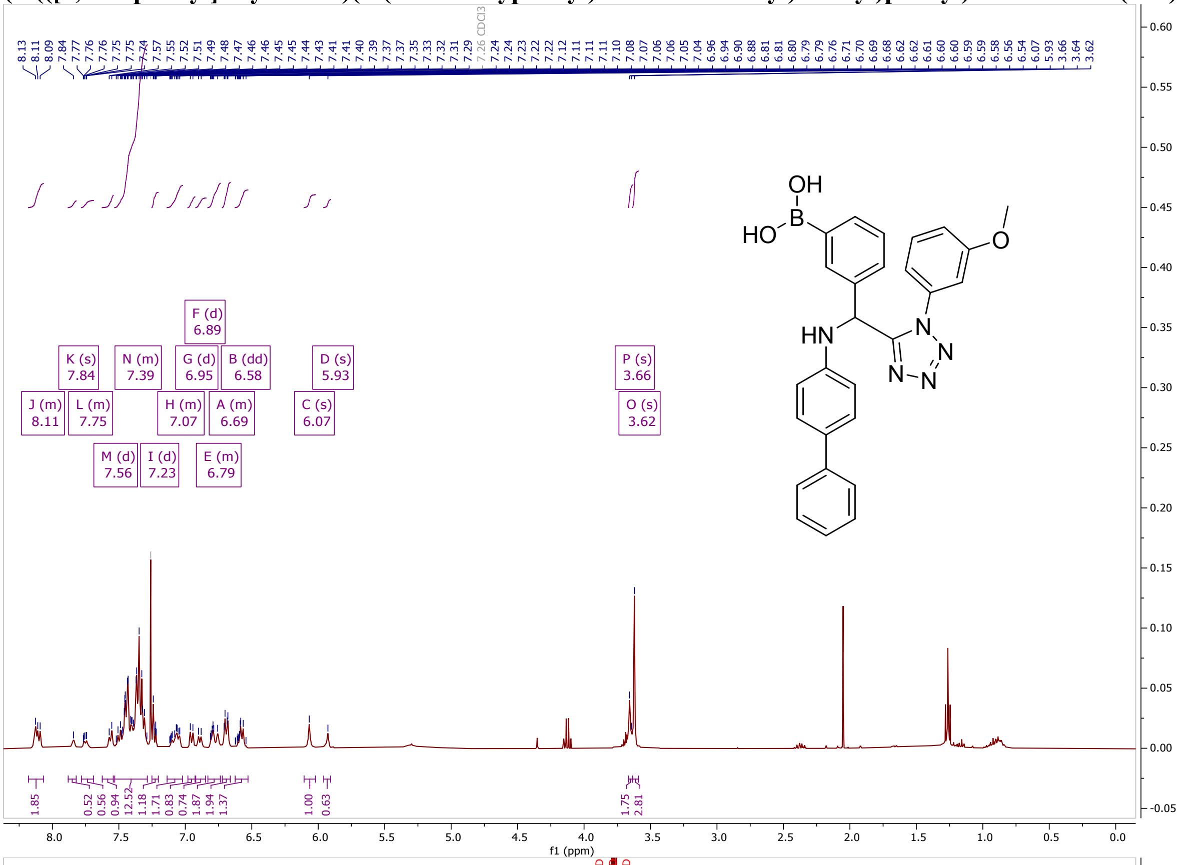

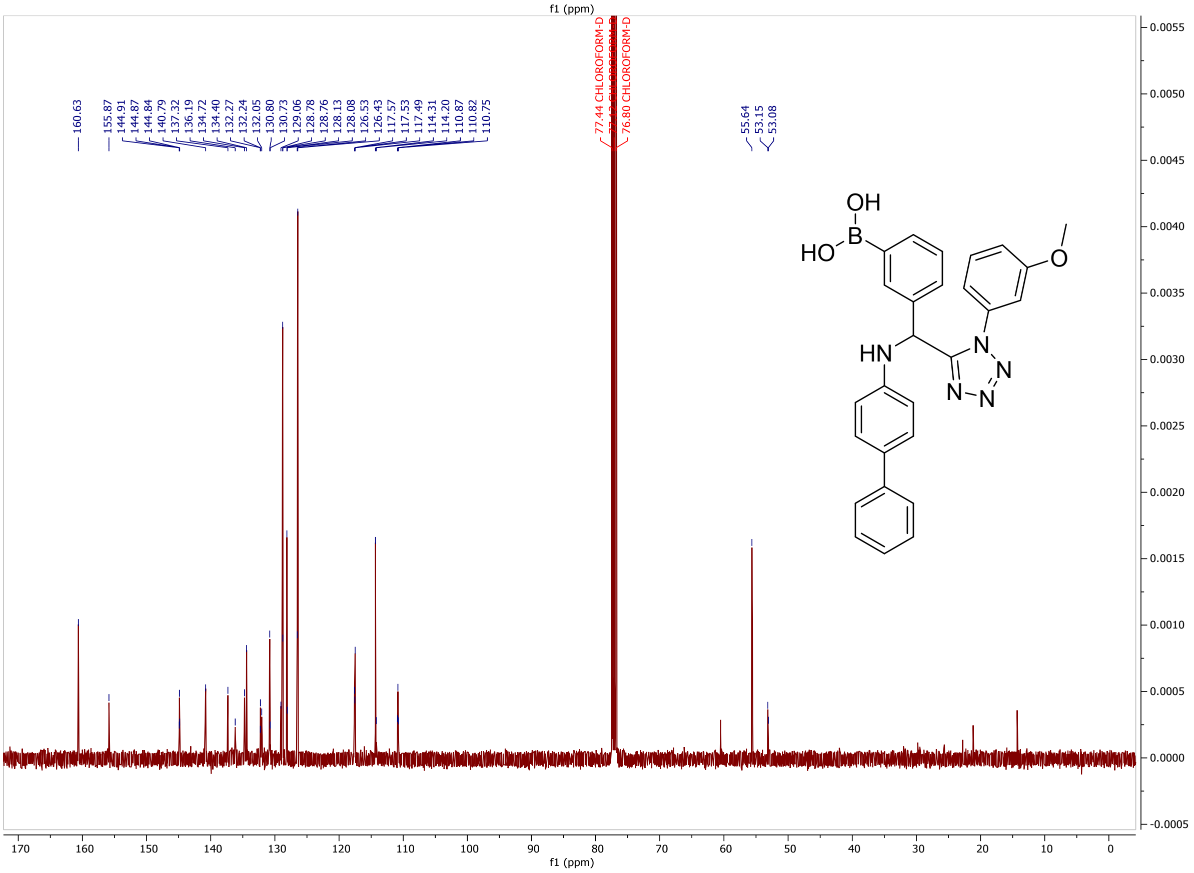

N-([1,1'-biphenyl]-4-yl)-N-((1-benzyl-1H-tetrazol-5-yl)(pyridin-3-yl)methyl)-2-chloroacetamide (2a)

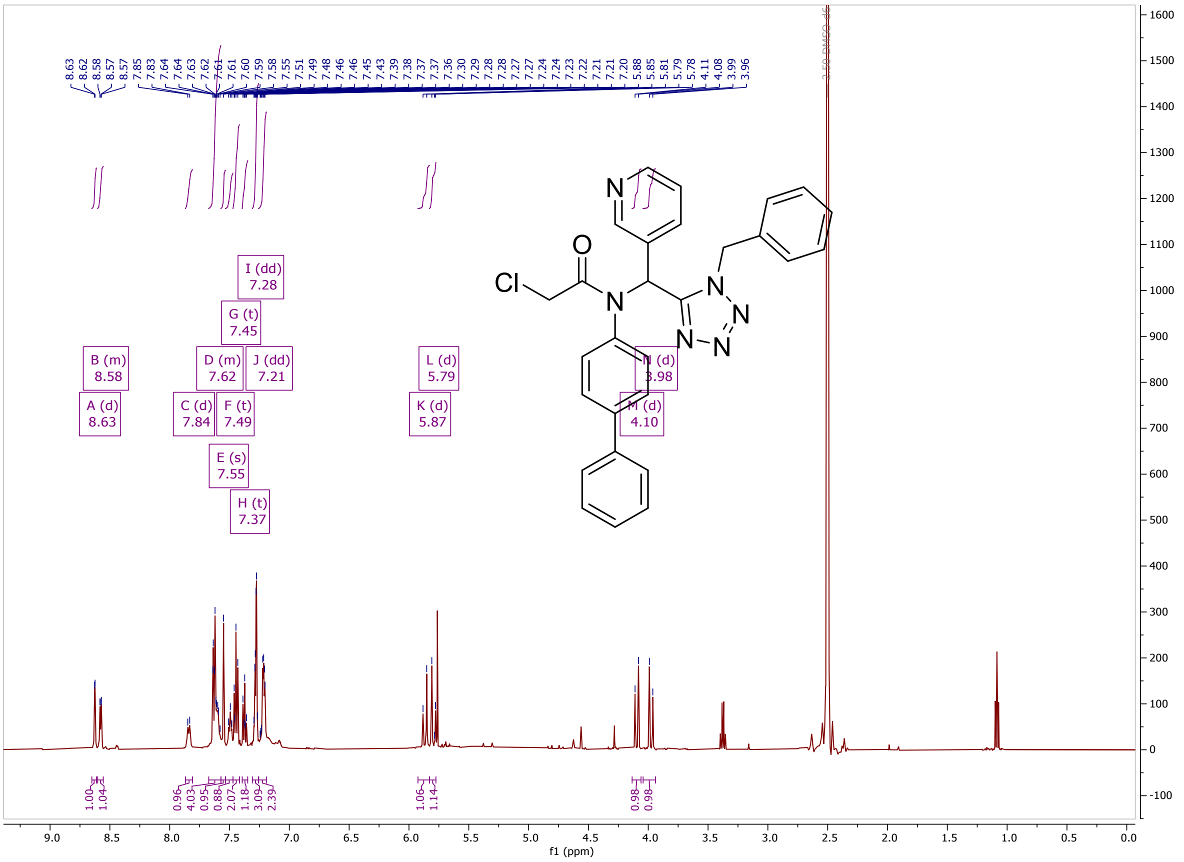

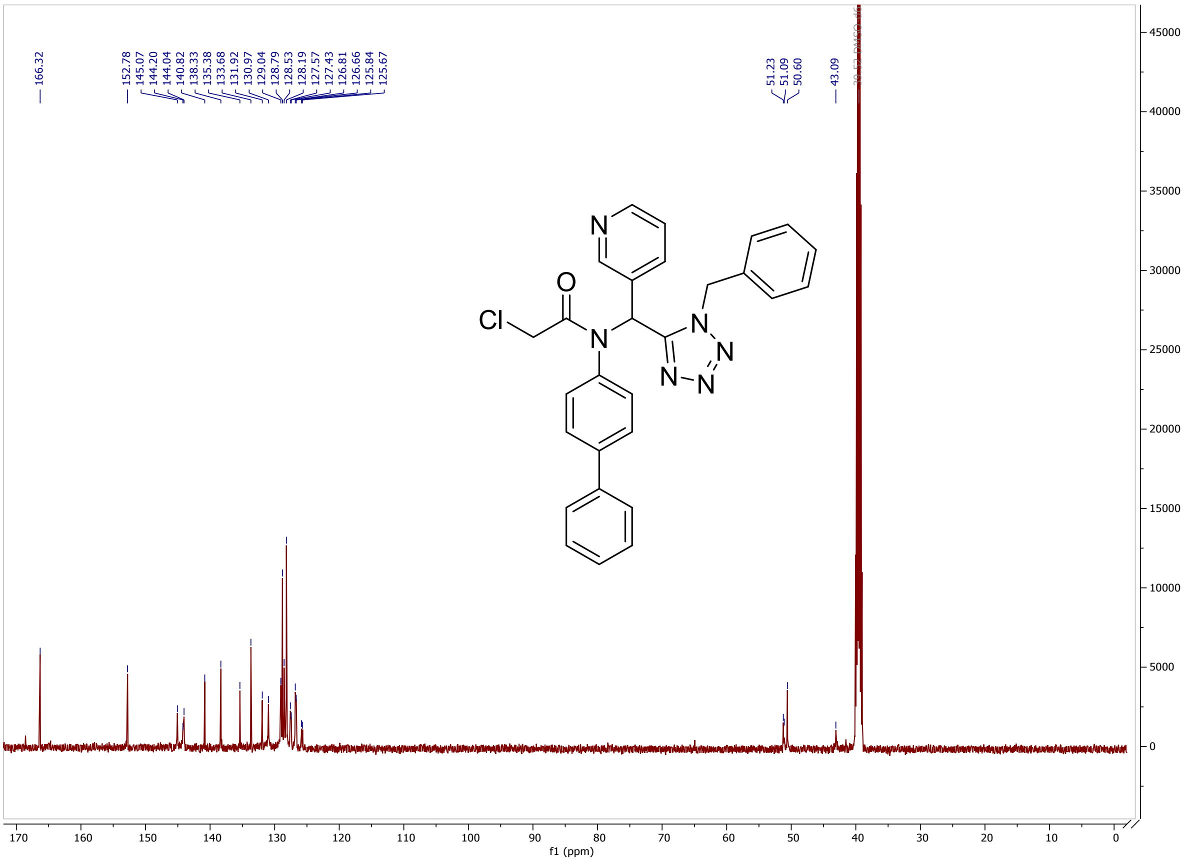

N-(4-(tert-butyl)phenyl)-2-chloro-N-((1-(3-fluorobenzyl)-1H-tetrazol-5-yl)(pyridin-3-yl)methyl)acetamide (2b)

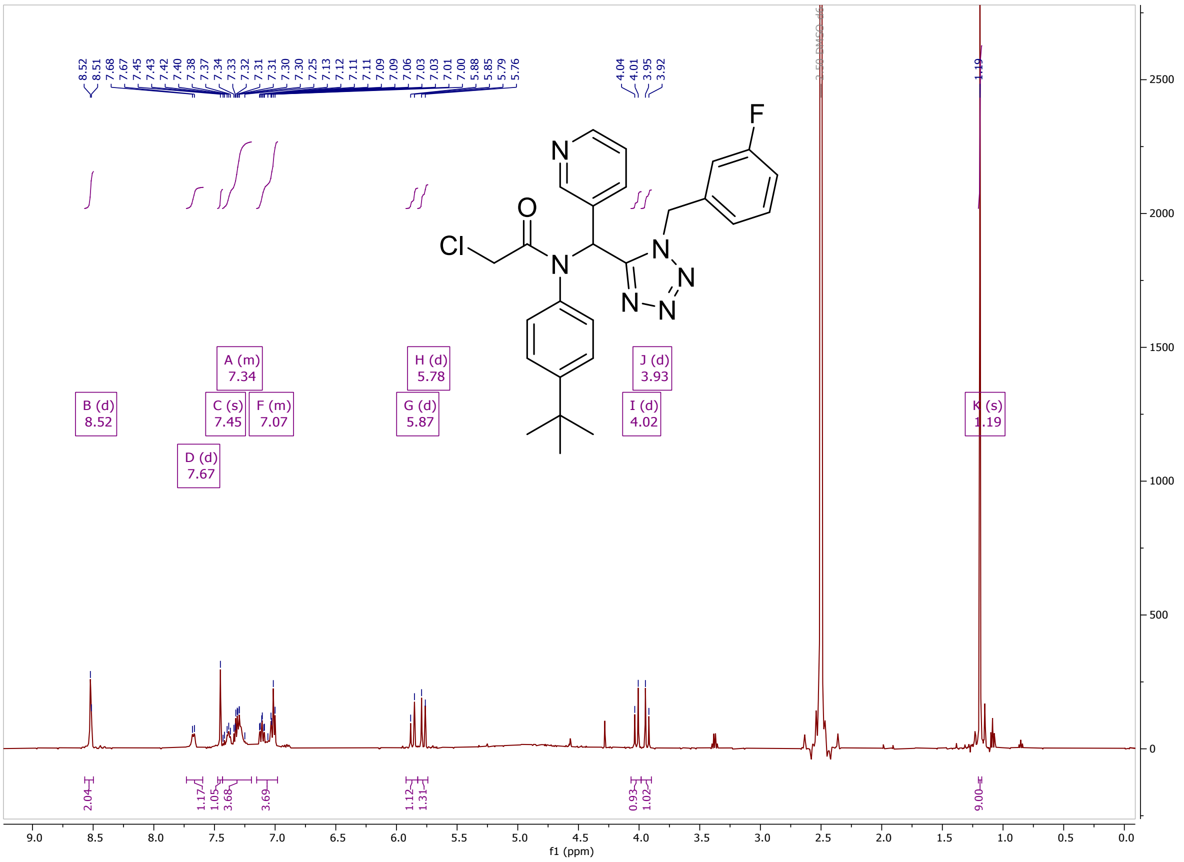

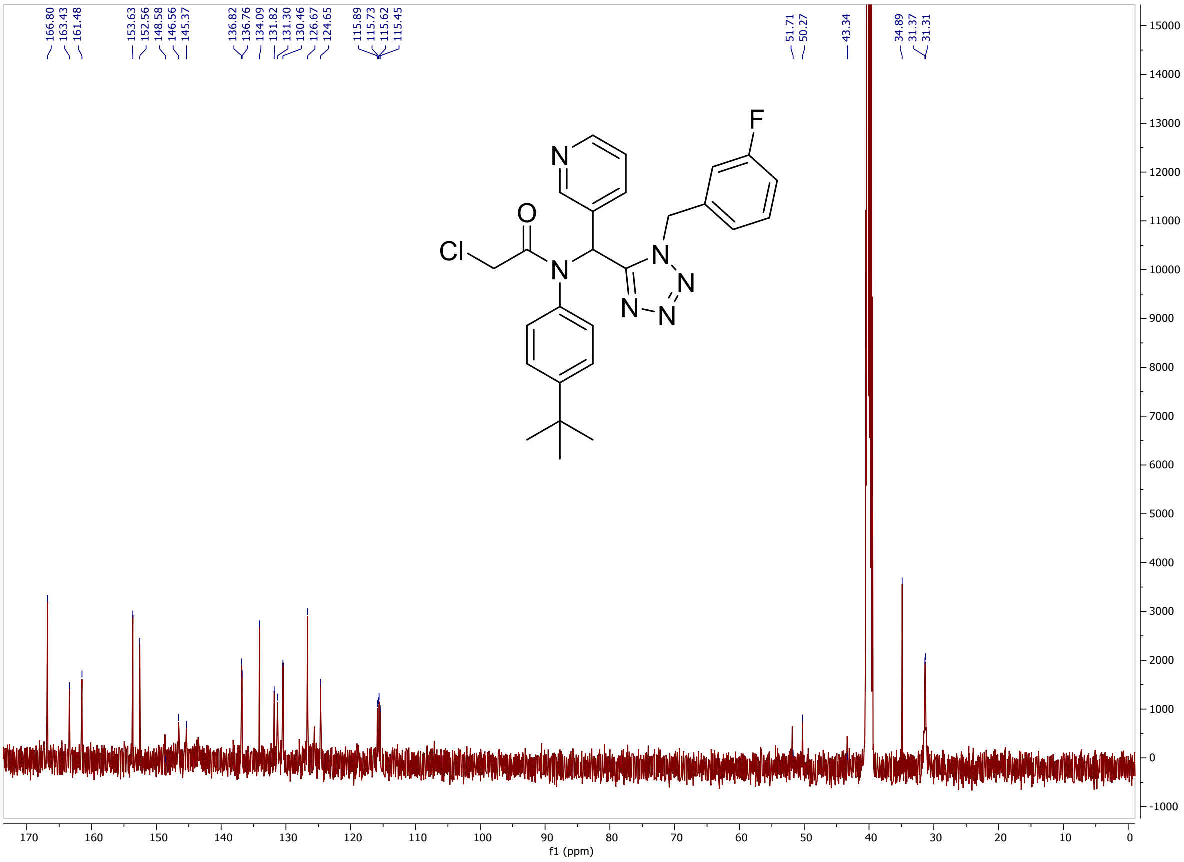

2-chloro-N-(2'-methyl-[1,1'-biphenyl]-4-yl)-N-((1-(1-phenylcyclopropyl)-1H-tetrazol-5-yl)(pyridin-3-yl)methyl)acetamide (2c)

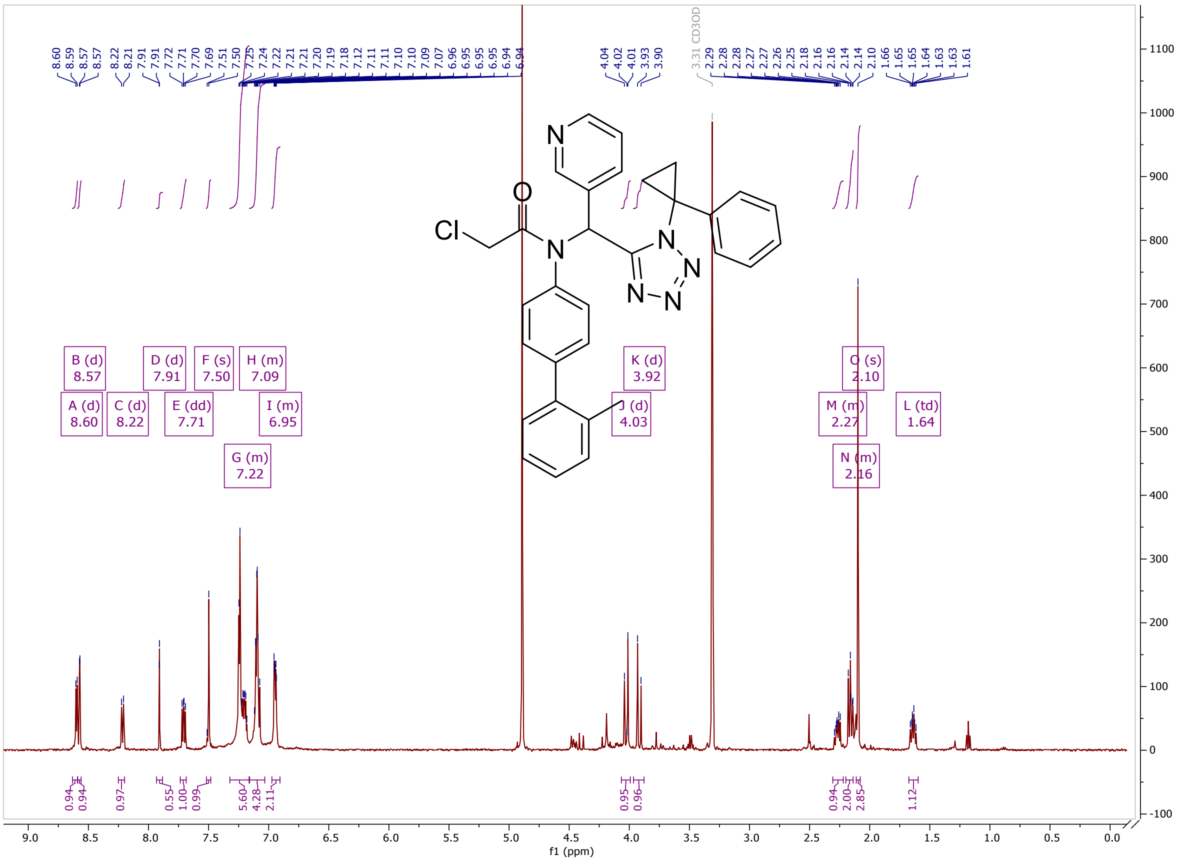

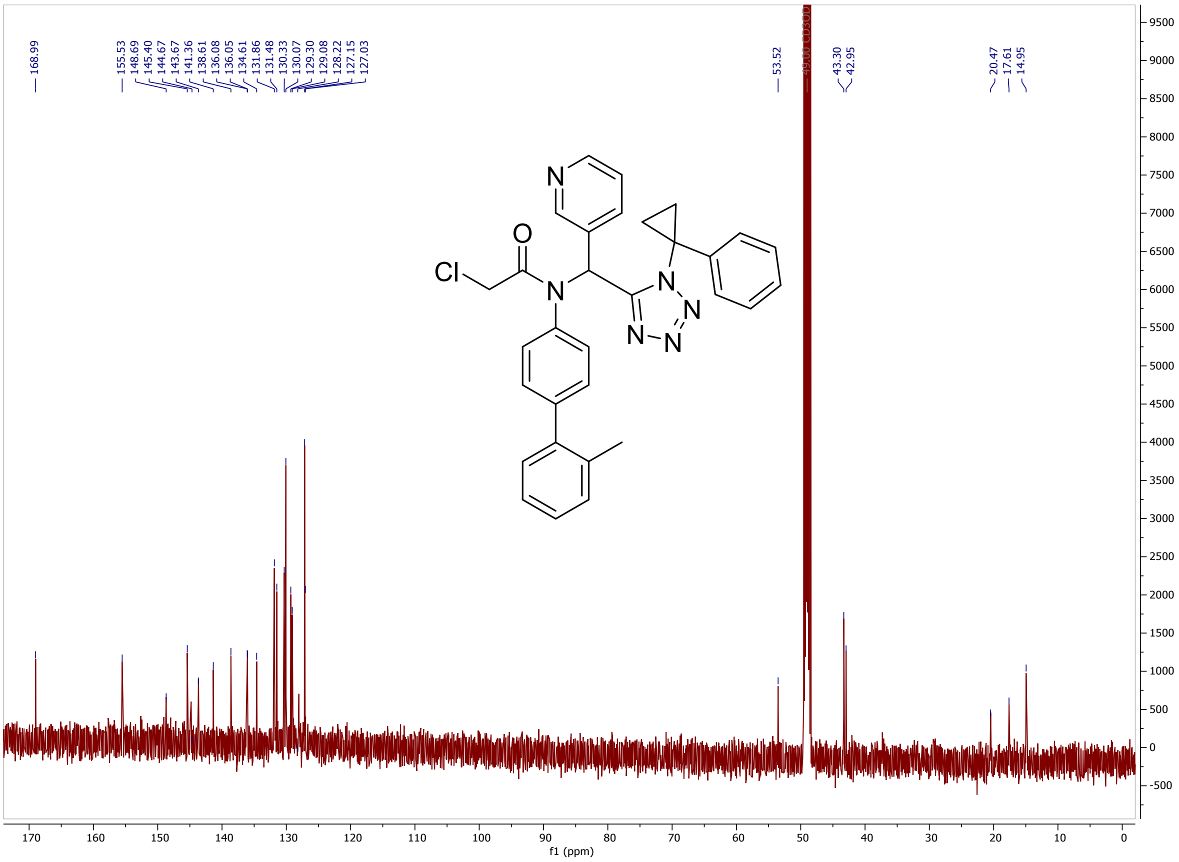

2-chloro-N-((1-(1-(2-fluorophenyl)cyclopropyl)-1H-tetrazol-5-yl)(pyridin-3-yl)methyl)-N-(2'-methyl-[1,1'-biphenyl]-4-yl)acetamide (2d)

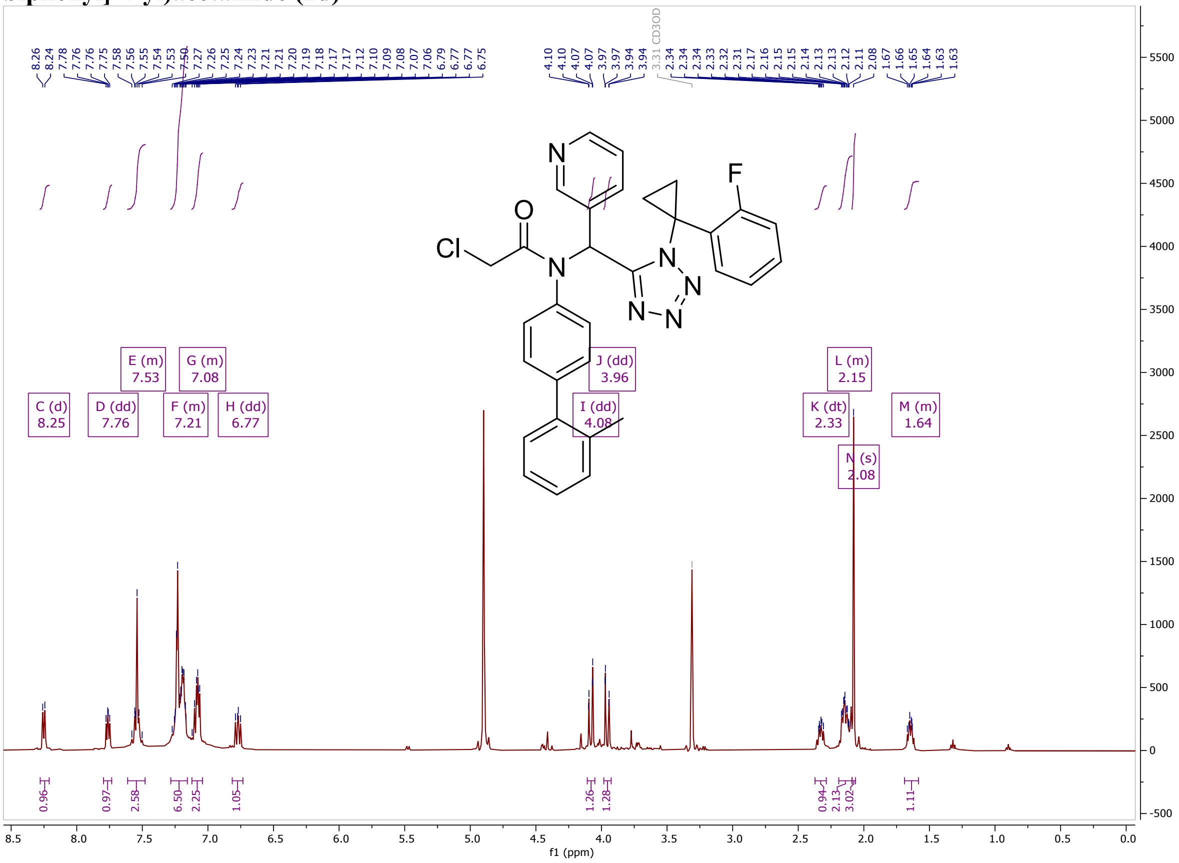

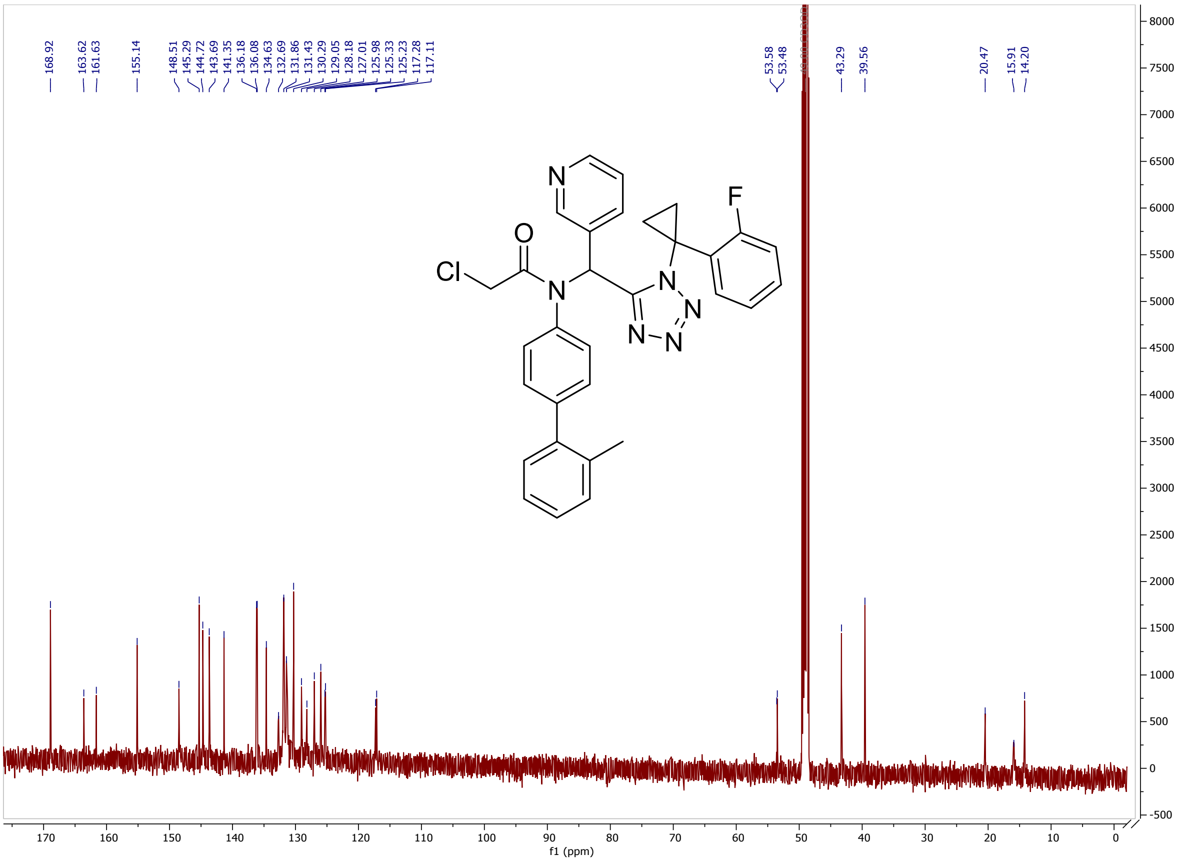

N-([1,1'-biphenyl]-4-yl)-2-cyano-N-((1-(1-phenylcyclopropyl)-1H-tetrazol-5-yl)(pyridin-3-yl)methyl)acetamide (3a)

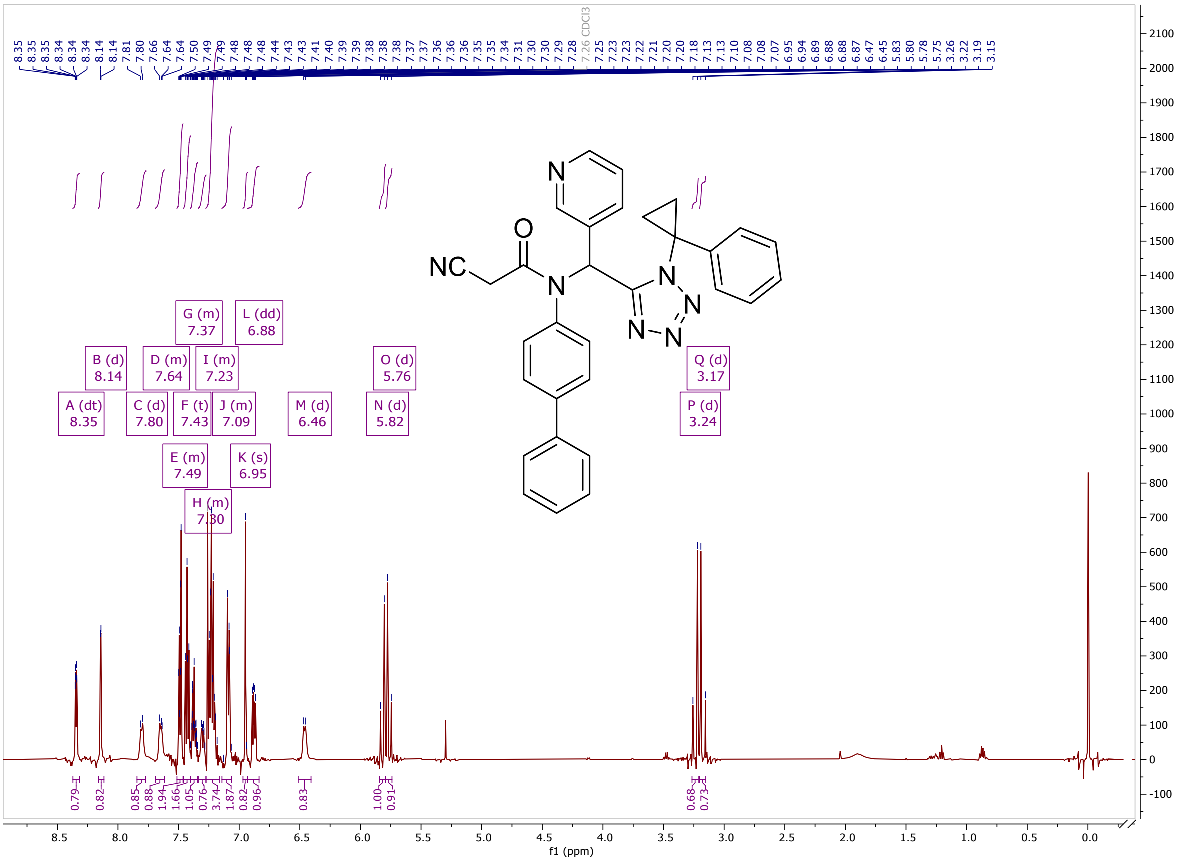

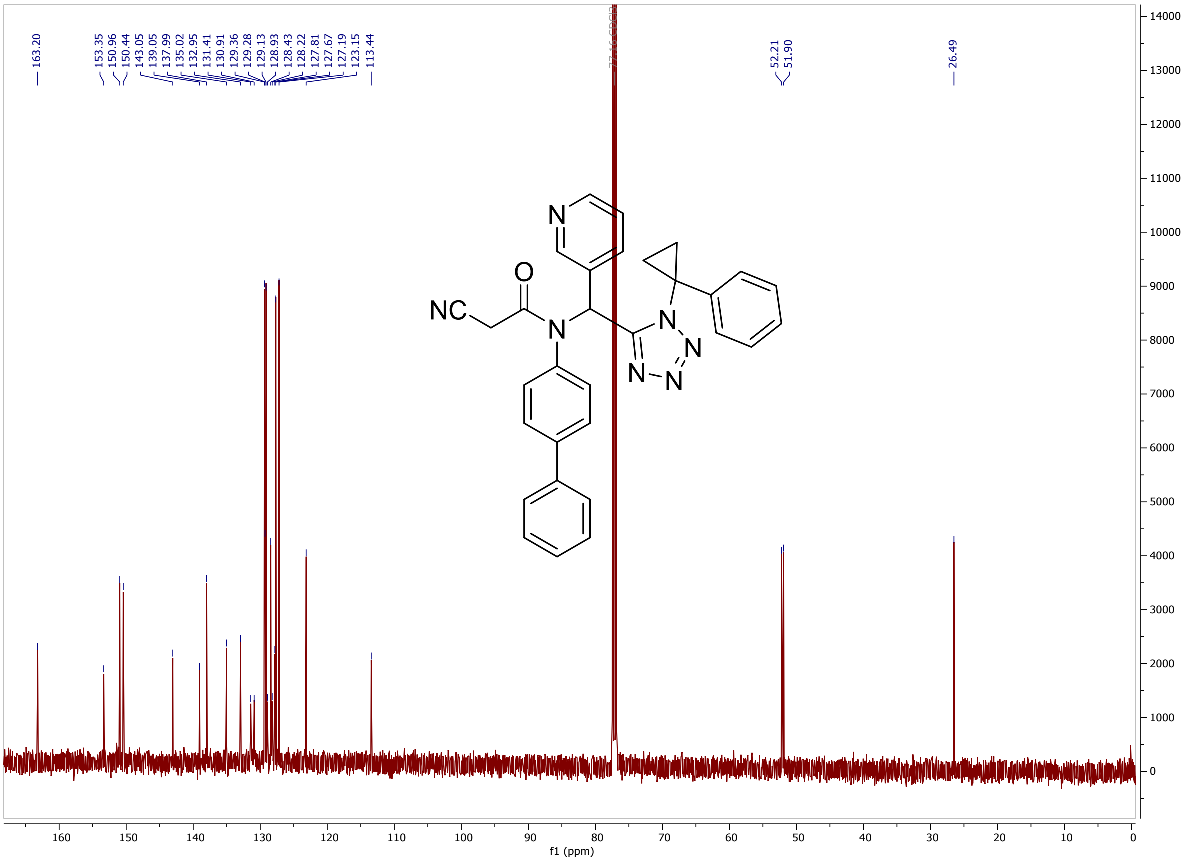

2-cyano-N-((1-(1-(2-fluorophenyl)cyclopropyl)-1H-tetrazol-5-yl)(pyridin-3-yl)methyl)-N-(2-methyl-[1,1'-biphenyl]-4-yl)acetamide (3b)

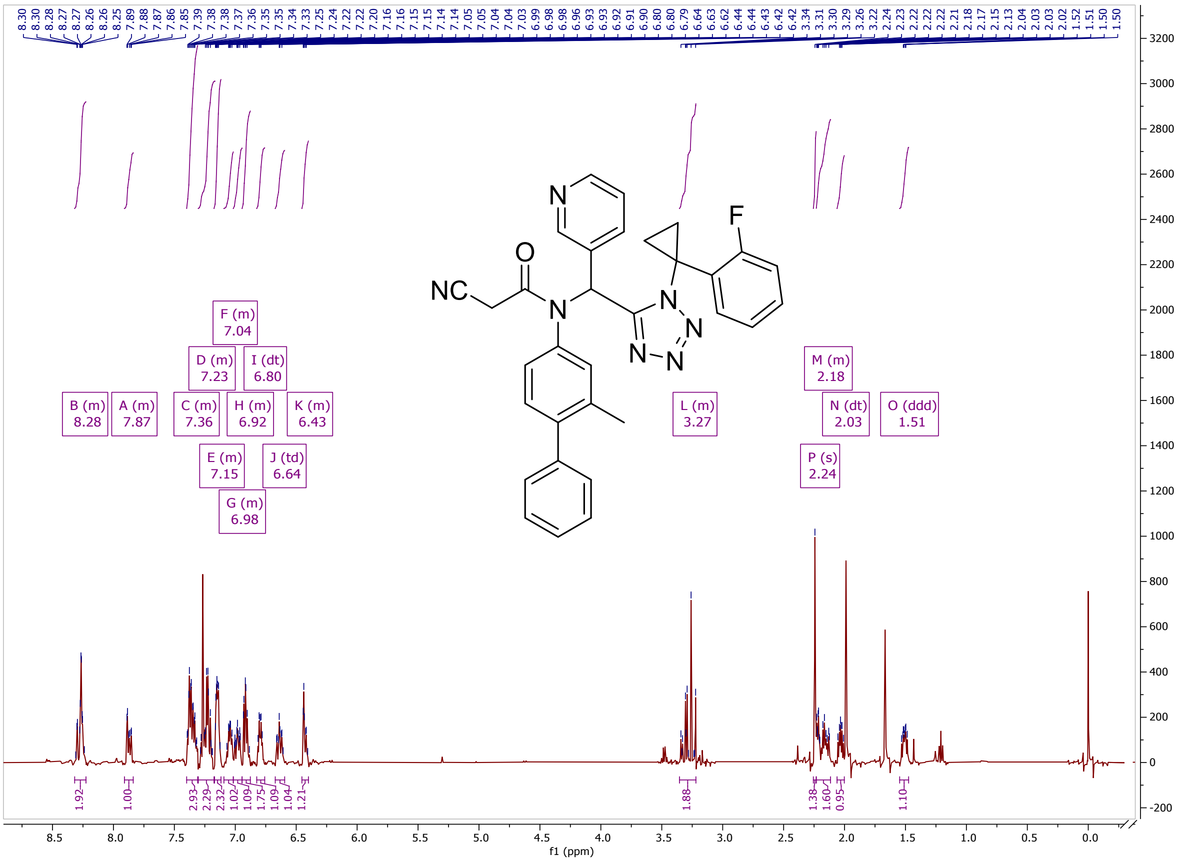

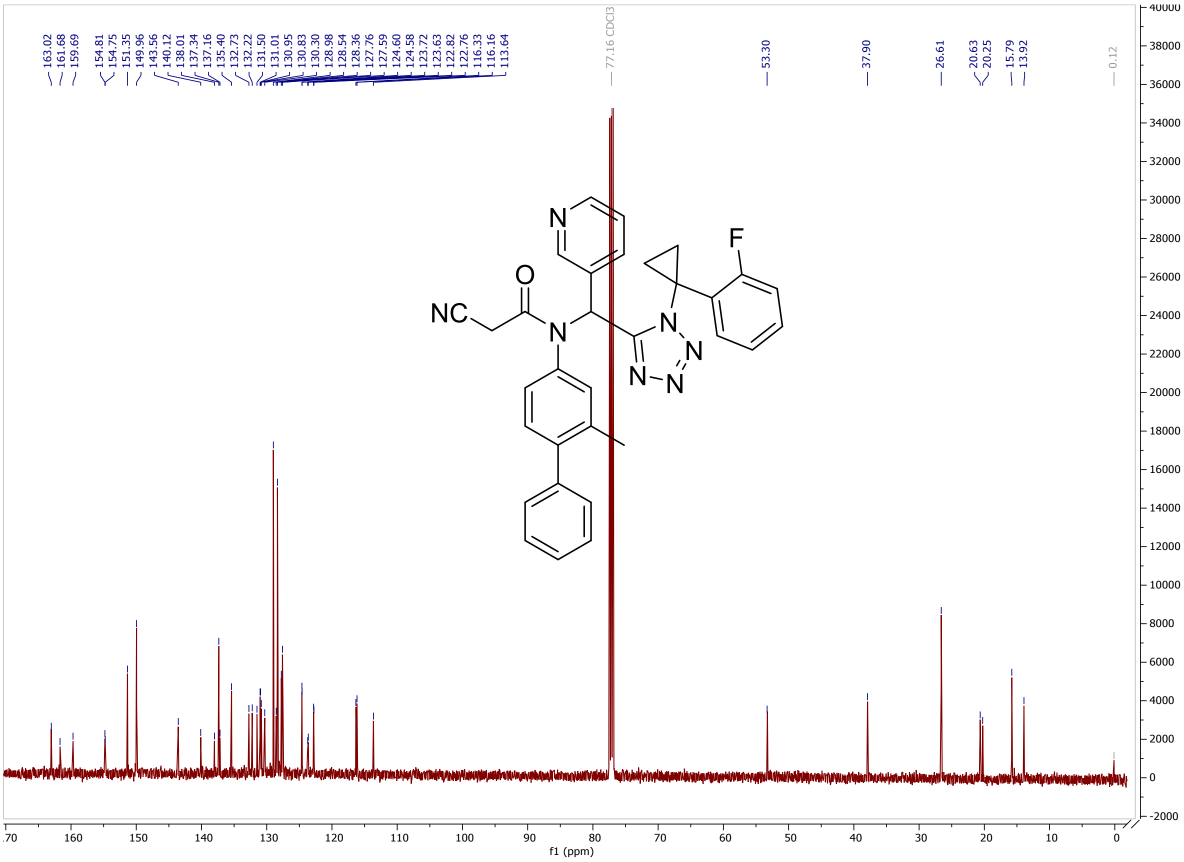

2-cyano-N-((1-(1-(2-fluorophenyl)cyclopropyl)-1H-tetrazol-5-yl)(pyridin-3-yl)methyl)-N-(2'-methyl-[1,1'-biphenyl]-4-yl)acetamide (3c)

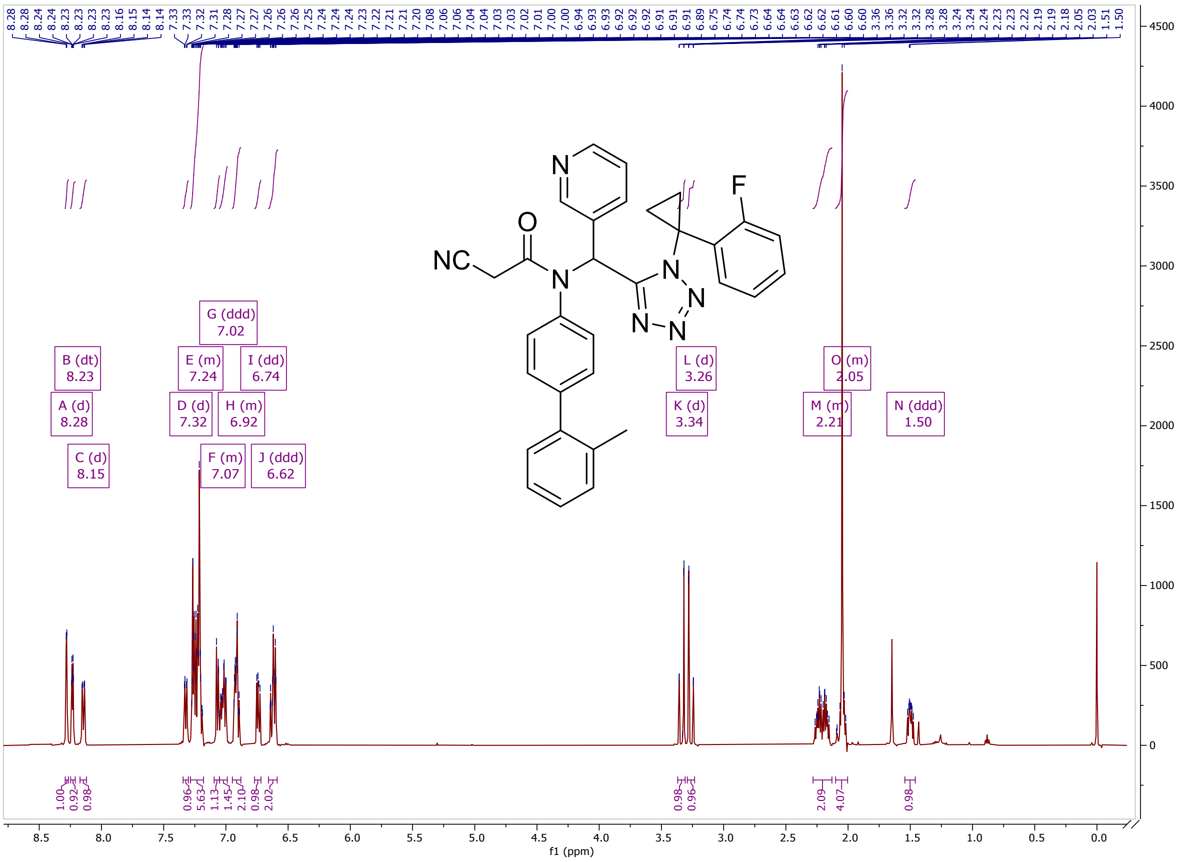

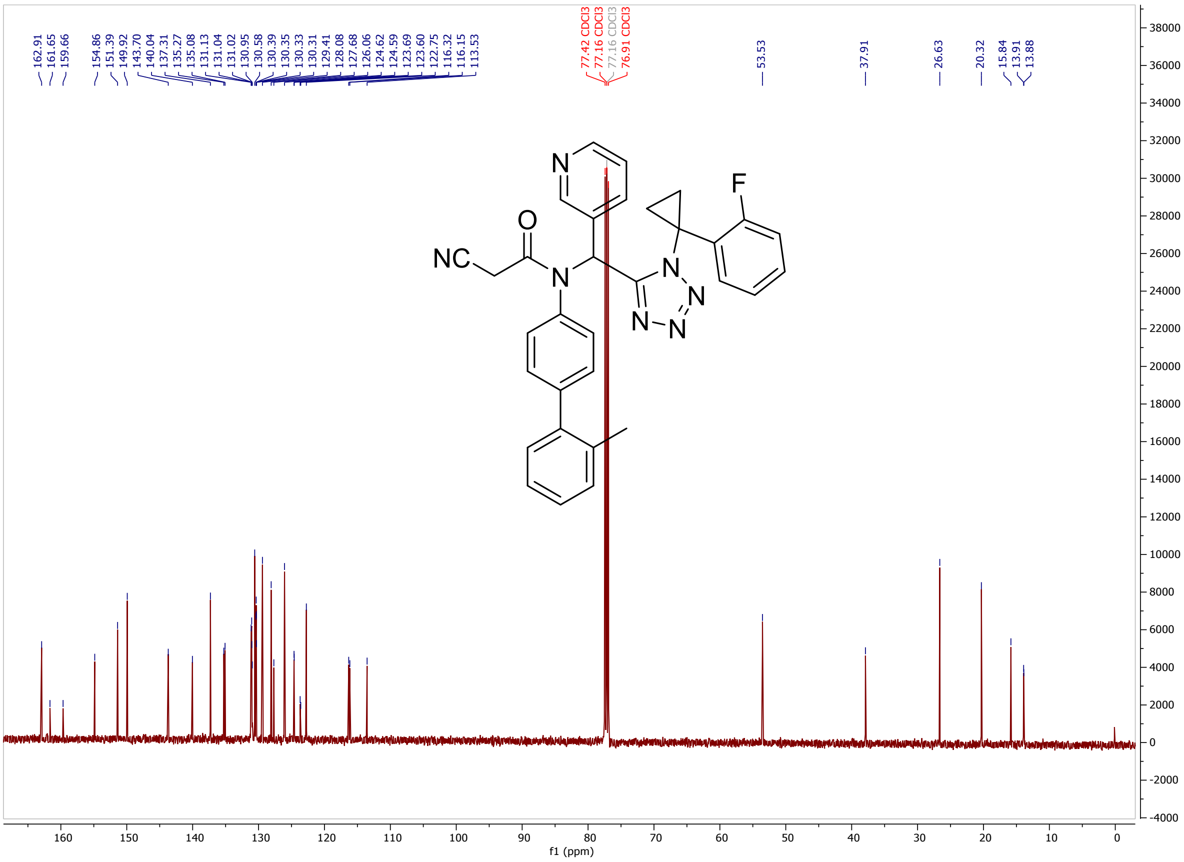

2-methyl-[1,1'-biphenyl]-4-amine (4a)

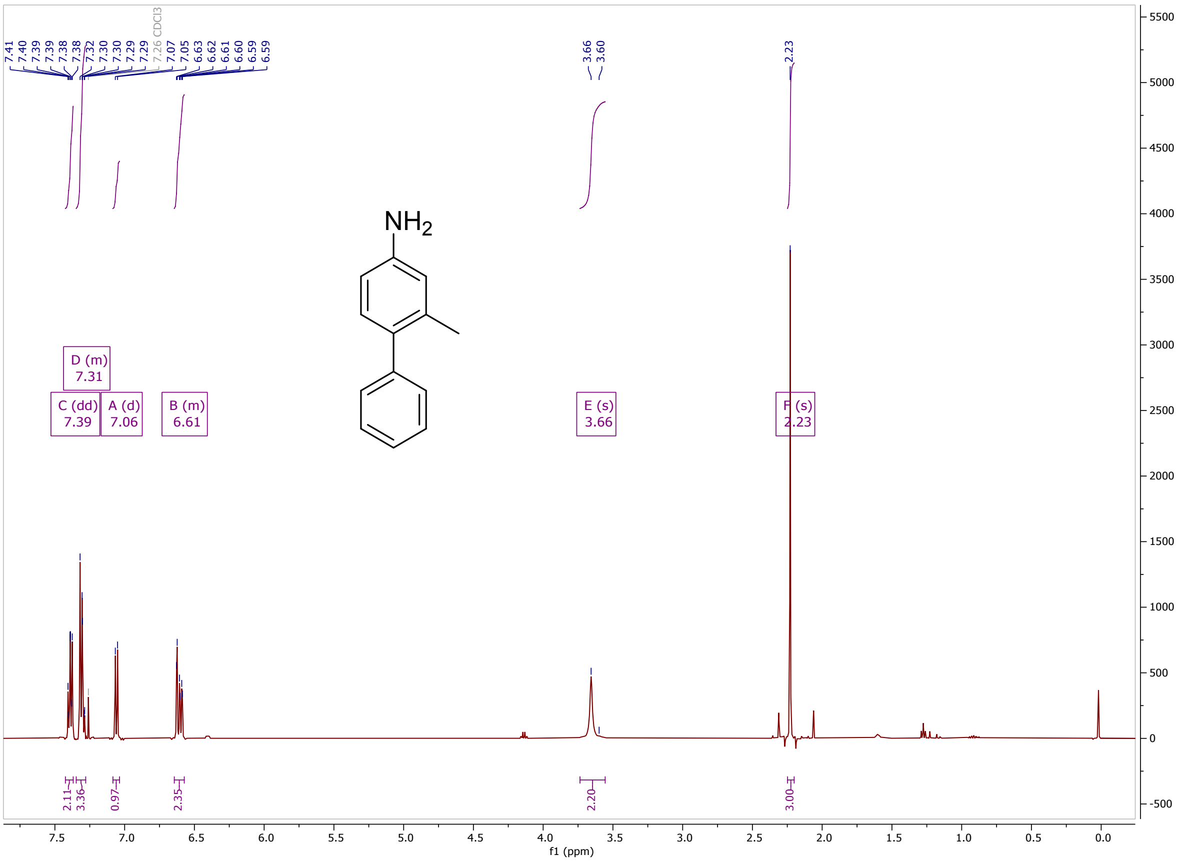

2-(trifluoromethyl)-[1,1'-biphenyl]-4-amine (4b)

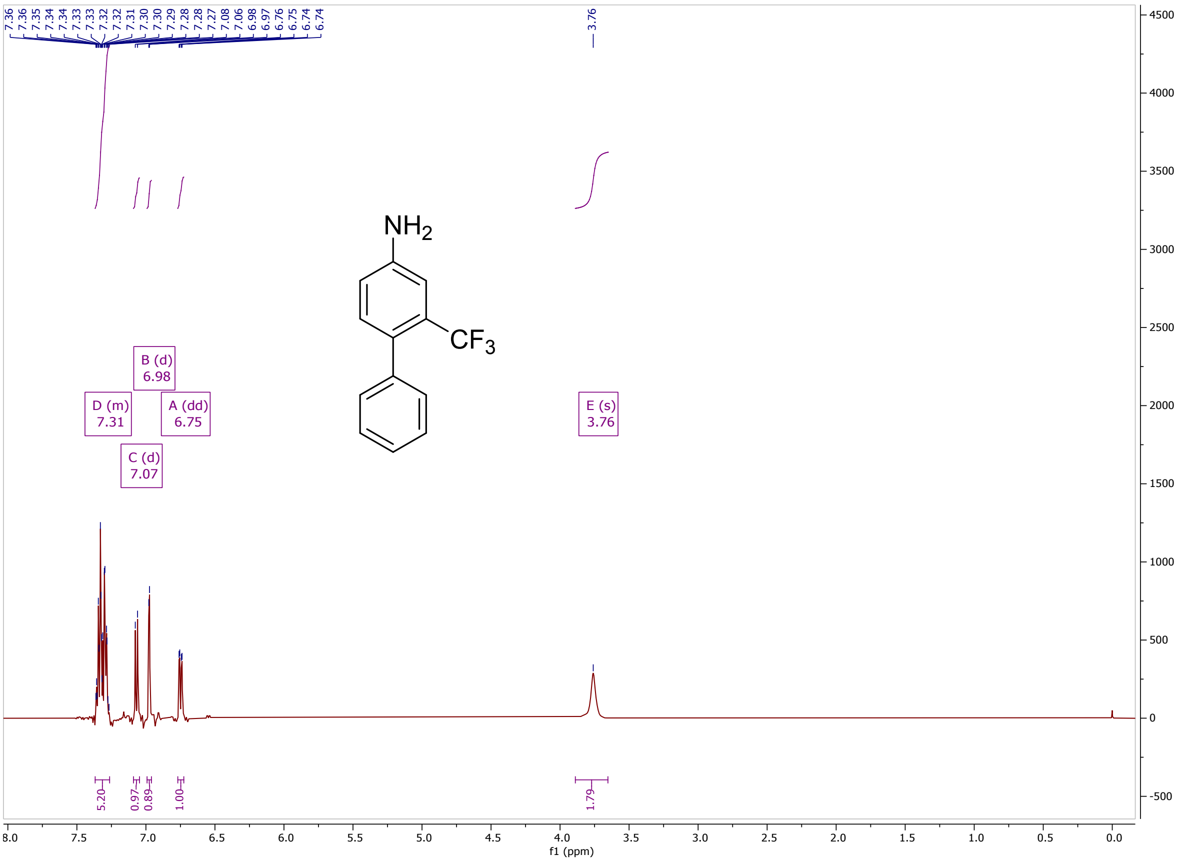

2-chloro-[1,1'-biphenyl]-4-amine (4c)

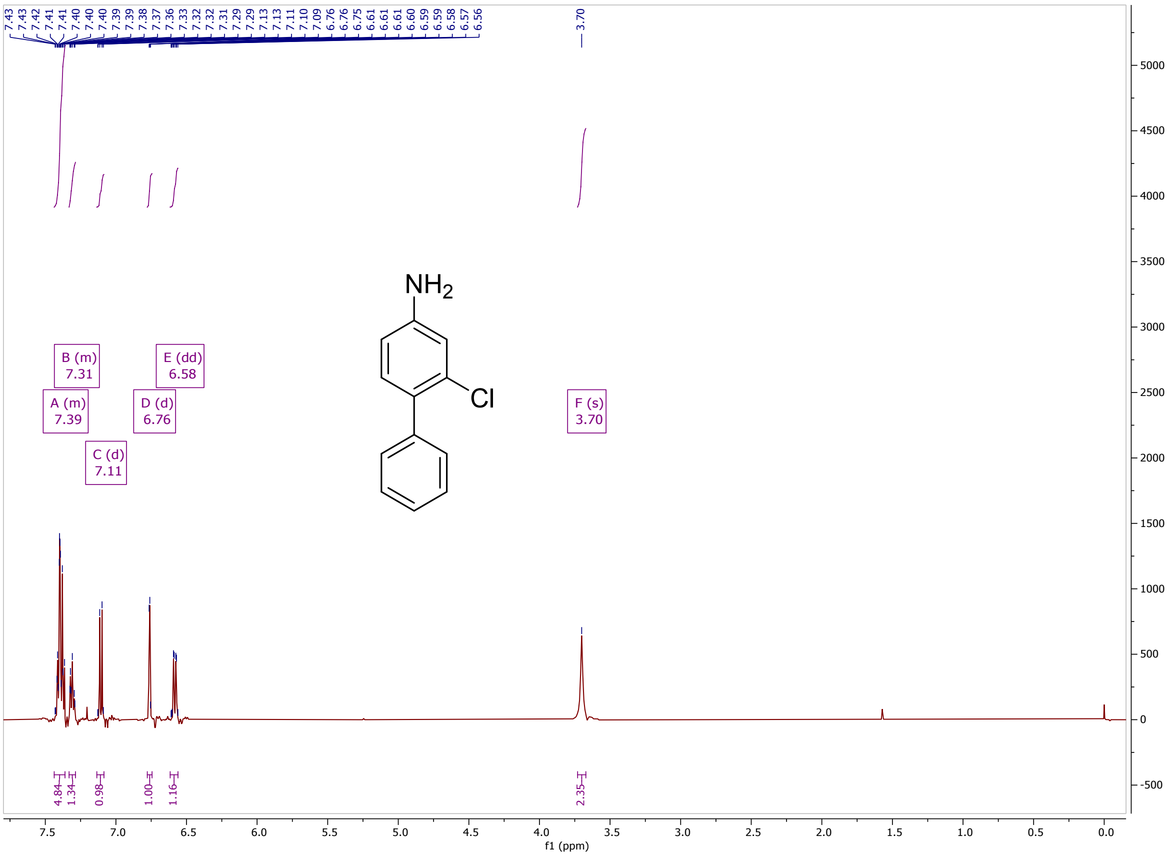

2'-methyl-[1,1'-biphenyl]-4-amine (4d)

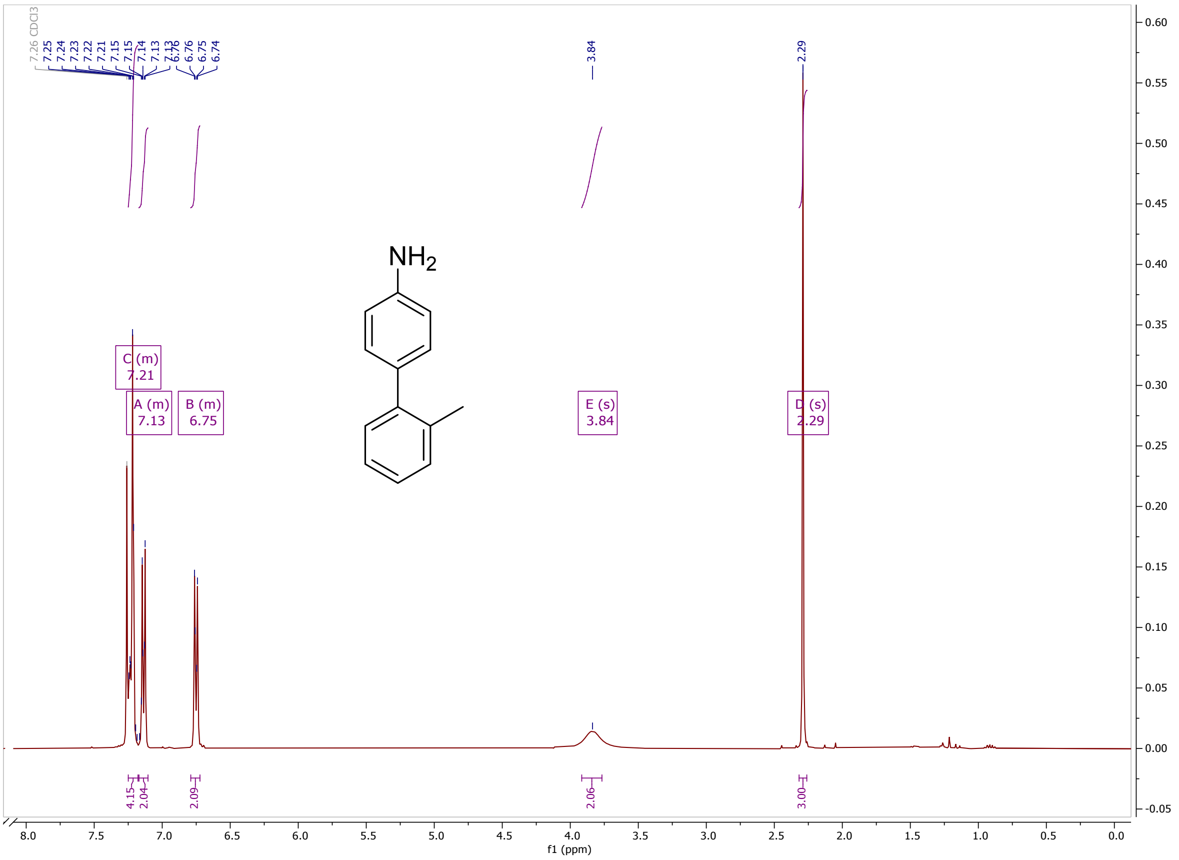

2'-(trifluoromethyl)-[1,1'-biphenyl]-4-amine (4e)

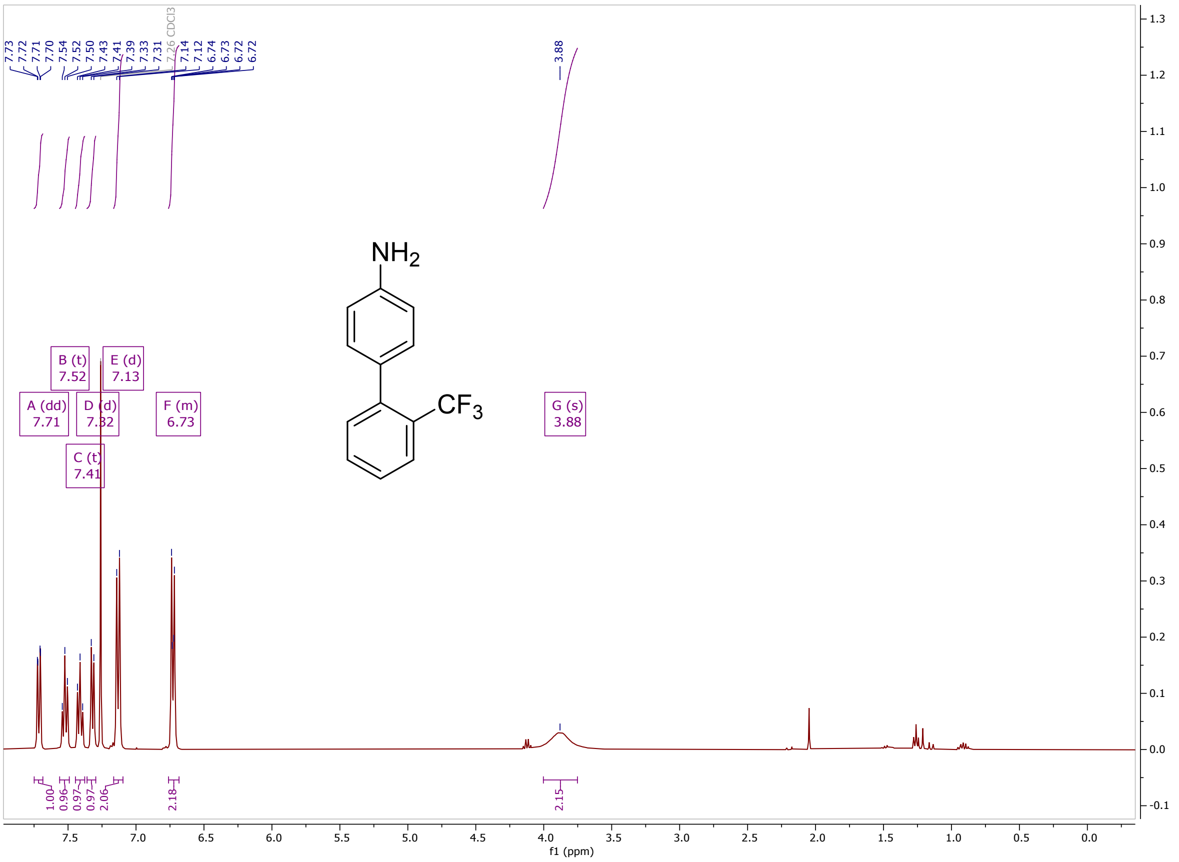

2'-chloro-[1,1'-biphenyl]-4-amine (4f)

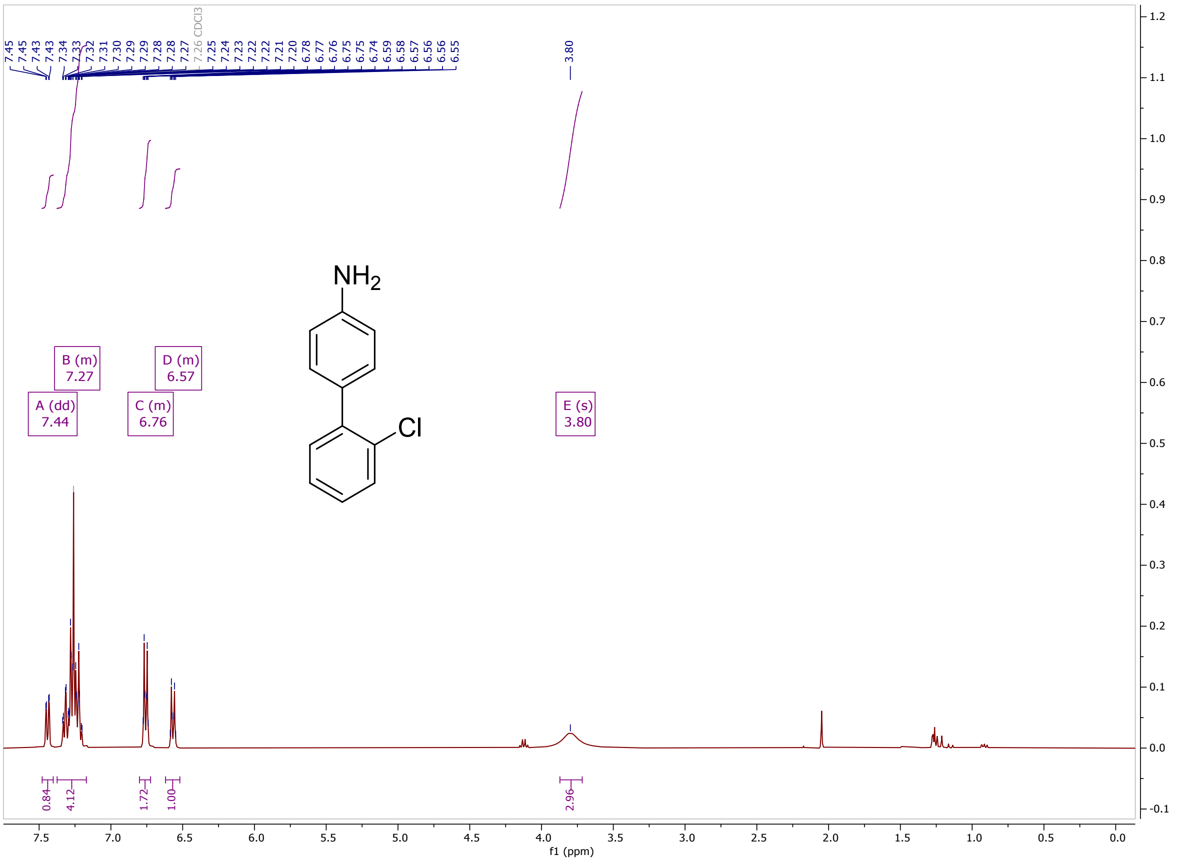

Chiral separation

Chiral separation of compound **2a** was performed on a Buchi Pure C-850 FlashPrep system. The optimal conditions were obtained eluting with n-hexanes/*i*-propanol (85:15, v/v) as mobile phase, 3.0 mL/min flow rate, at room temperature using a Chiralpak IC chiral column 10.0x250mm, 5µm as stationary phase. The product synthesized shows an enatiomeric mixture with retention times of 29.00 and 38.00 minutes. Where separated product shows for **2a-(A)** at the retention time of 29.00 minutes and **2a-(B)** at 38.00 minutes Fig. S1.

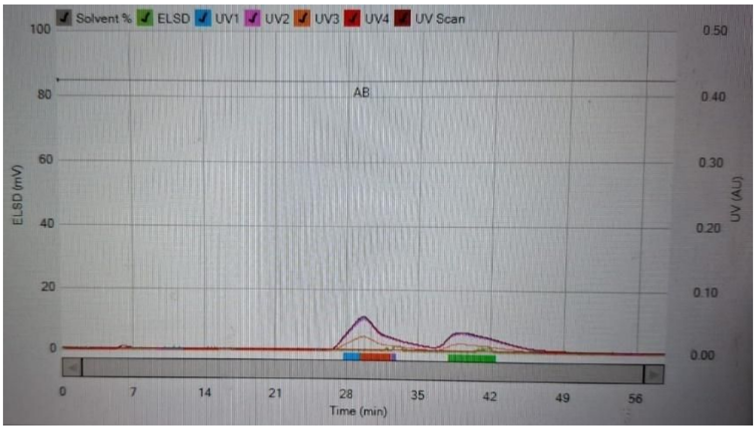

Figure S1. Chromatogram of the separation of **2a** on a Chiralpak IC chiral column.

Protein expression and purification for the purpose of activity assays

An *E.coli* codon-optimised gene encoding the SARS-CoV-2 3CLpro was purchased from Eurofins genomics. The construct was then modified to contain a C terminal human Rhinovirus 3C protease cleavage site, expressed and purified in the manner previously described[4].

Activity assay

3CLpro enzymatic activity was assessed using a kinetic FRET assay, adapted from our previously published protocol[5]. In short, a Synergy H1 platereader (BioTek) was used to monitor the 3CLpro catalysed cleavage of the peptidic FRET substrate 2-aminobenzoyl-SVTLQSG-Tyr(NO2)-R (Genscript). The fluorescence was measured at wavelengths of 330 nm excitation and 420 nm emission. Final assay volume was 200 µL, containing 250 nM 3CLpro and 100 µM substrate in reaction buffer (100 mM Potassium Phosphate pH 8, 3 mM Beta-mercapthoethanol (BME) and 4% (V/V) dimethylsulfoxide (DMSO)). The reaction was monitored continuously for 10 minutes immediately following substrate addition. Initial rates were then determined by linear regression using the first 5 minutes. To measure the inhibitor potency of the compounds, 3-fold serial dilutions were prepared in DMSO and pre-incubated with the enzyme for 30 minutes at 25°C prior to substrate addition. Non-covalent inhibitor concentrations ranged from 500 µM to 200 nM and covalent inhibitors from 10 µM to 4 nM. Data were normalized using control reactions - without enzyme (0% inhibition) and without inhibitor but with 4% DMSO (100% activity). Dose response curves were generated by plotting the initial rates against inhibitor concentrations. IC50 values were calculated by curve-fitting the dose response curves in python using 3 parameter logistics regression. All measurements were performed in triplicate. Outliers in the form of wells with observed air bubbles were removed prior to analysis.

Protein expression and purification for the purpose of crystallisation

Protein production for the purpose of crystallisation was performed by the ASAP Discovery consortium using their standard COVID Moonshot protocol[6,7].

Crystallisation

SARS-CoV-2 3CLpro was crystallised using the sitting-drop vapour diffusion method in SwissCI 3-lens plates. The drops were made using the SPT Mosquito and consisted of 150 nL protein (5 mg/mL), 150 nL reservoir condition (100 mM MES pH 6.7, 11% PEG 4000 and 5% DMSO) and 50 nL seed stock (1:250 dilution[8]) prepared from previously produced crystals under the same conditions. Crystals formed within 24h and grew to their maximum size after 48h. Co-crystal structures of **2a** in complex with 3CLpro were obtained by soaking. **2a** was dissolved in DMSO to 100 mM and 90 nL was directly applied to the crystallisation drop using an ECHO liquid handler, yielding a final compound concentration of 20 mM in the drop. The mixture was incubated for 3 hours at RT before being flash frozen in LN2. Data were collected at the Diamond Light Source on the I03-1 beamline at 100K. The data were processed using the autoPROC-STARANISO pipeline implemented at the beamline[9-14]. The anisotropically truncated data as determined by STARANISO were then used to generate electron density maps with DIMPLE[15], using a previously solved 3CLpro structure as a reference model. CIF restraints for **2a** were created with ACEDRG[16], followed by manual ligand modeling into the density using COOT[17]. Following initial ligand placement, iterative refinement and model building were carried out with REFMAC[18] and COOT, respectively. Polder maps surrounding the ligand were generated using phenix[19]. The final structure was deposited in the PDB (accession code 9QD5). Data collection and refinement statistics are available in Table S1.

Table S1. Data collection and refinement statistics of 9QD5

**Data Collection**	SARS-CoV-2 3CLpro – RS222C (PDB: 9QD5)
Wavelength	0.97625
Space Group	P 21 21 21
Unit Cell (Å, ⁰)	67.742 99.931 104.108 90.0 90.0 90.0
Anisotropic diffraction limits & principal axes of ellipsoid fitted to diffraction cut-off surface	
_a_*	2.114 | 1.0000 0.0000 0.0000
_b_*	2.701 | 0.0000 1.0000 0.0000
_c_*	2.043 | 0.0000 0.0000 1.0000
Resolution (Å)	72.093 – 2.044 (2.230 – 2.044)
Reflections Total	412470 (20848)
Reflection Unique	31215 (1561)
Rmerge	0.199 (1.823)
Completeness (Spherical) (%)	68.7 (15.2)
Completeness (Ellipsoidal) (%)	93.2 (60.4)
<I / σ(I)>	9.7 (1.6)
Multiplicity	13.2 (13.4)
CC(1/2)	0.918 (0.665)
**Refinement**	
Reflections work / free	29621 / 1594
Rwork / Rfree	0.190 / 0.232
No. Atoms	4935
Protein	4613
Ligands	41
Waters	281
B-Factors	
Protein	35.32
Ligands	64.06
Waters	36.38
RMS deviations	
Bond lengths (Å)	0.0095
Bond angles (⁰)	1.377
Ramachandran	
Favoured (%)	98.64
Allowed (%)	1.36
Outliers (%)	0.0

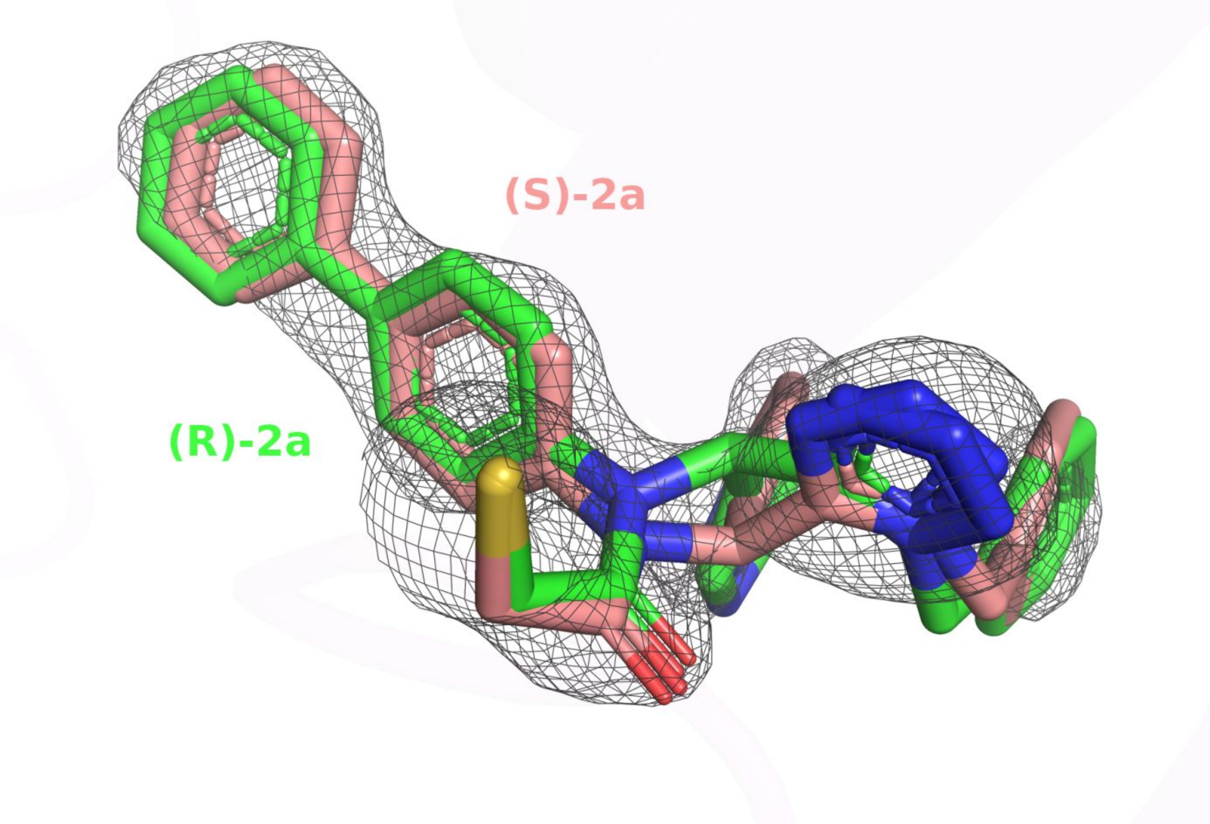

Figure S2. Electron density of (rac)-**2a** shown as a 2Fo–Fc map contoured at 1.0 σ (gray mesh), with both the **R** (green sticks) and **S** (salmon sticks) enantiomers modelled.

Cell culture and the cytotoxic assay

A549-hACE2 cells, which were the A549 lung carcinoma cell line stably transfected to express the human ACE2 (hACE2) gene, were kindly provided by Prof. Trine Mogensen (Aarhus University, Aarhus, Denmark) and the African green monkey Vero E6 cell line (ATCC, Manassas, VA) were maintained in growth medium containing Dulbecco's minimal essential medium (DMEM) high glucose (Life Technologies, Carlsbad, CA) supplemented with 10% fetal bovine serum (FBS) (Lonza, Basel, Switzerland), 100 U/mL penicillin, 100 U/mL streptomycin (Life Technologies). Cells were mycoplasma negative and maintained at 37 °C under 5% CO_2.

For the cytotoxic assay, A549-hACE2 cells were seeded in 96-well plate at a density of 1×10^4 cells per well and cultured in growth medium at 37 °C under 5% CO_2 for 24 h before adding serial dilutions (0-250 µM) of **2a**. Cells were allowed to grow for another 24 h before analyzing the cell proliferation using the 3-(4,5-dimethylthiazol-2-yl)-2,5-diphenyltetrazolium bromide (MTT) method. Briefly, the growth medium was removed and then adding 100 µL of 0.5 mg/mL MTT solution (Sigma-Aldrich, Burlington, MA) for another 4 h at 37 °C before adding 100 µL of DMSO to the cells. To solubilize the formazan crystals, cells were incubated for another 30 min, followed by measuring the absorbance of each well at OD_{600} using GloMax-Multi Detection System (Promega, Madison, WI) and the cell survival percentage was calculated as OD_{600} of the sample/control.

Virus stocks and titration

SARS-CoV-2 B.1.1.7 (Alpha) strain was kindly provided by Prof. Trine Mogensen. SARS-CoV-2 was propagated in Vero E6 cells in infectious medium containing DMWM high glucose supplemented with 2% FBS and the virus titer was determined on Vero E6 cells and the culture infectious dose ($TCID_{50}$) units per mL of supernatant were calculated according to the Reed-Muench method[20].

Antiviral assays

A549-hACE2 cells were seeded at a density of 1 × 10^5 cells per well in 12-well plates in growth medium before SARS-CoV-2 infection at MOI 1 in infectious medium. The inoculum was removed after 1 h followed by twice washing of 1 × PBS (137 mM NaCl, 10 mM phosphate pH 7.4, 2.7 mM KCl) and replaced with the fresh infectious medium with DMSO as control, BafA1 or **2a**. Cells and the supernatants were collected at 24 hpi. Specifically, the cells were harvested in 100 µL of 2 × sample buffer (65.8 mM Tris–HCl, pH 6.8, 26.3% glycerol, 2.1% SDS and 0.01% bromophenol blue) for 30 min on ice followed by 1 min of sonication and 8 min of boiling before loading to SDS-PAGE to perform western blot using primary antibodies against SARS-CoV-2 N protein (Cell Signaling Technology, Danvers, MA) and Vinculin (Cell Signaling Technology), and secondary antibody conjugated to Alexa-680 (Life Technologies). Supernatants were harvested and titrated using $TCID_{50}$ assay and the data were normalized to the DMSO-treated control.

A549-hACE2 cells were also seeded at a density of 8 × 10^3 cells per well in 96-well plates in growth medium before SARS-CoV-2 infection as indicated above before lysing with 30 µL lysis buffer containing DNase I at RT for 5 min followed with additional 3 µL of STOP solution at RT for 3 min, according to the manufacturer's protocol (Power SYBR Green Cells-to-CT kit, Life Technologies). Reverse transcription of the RNA, cDNA synthesis and quantitative PCR were performed in a CFX connect Thermocycler (Bio-Rad, Hercules, CA). Primes used were 5' AGCCACATCGCTCAGACAC and 3' GCCCAATACGACCAAATCC for GAPDH, 5' AAAATCTGTGTGGCTGTCACT and 3' GACGAAACCGTAAGCAGCCT for SARS-CoV-2 gRNA1 79-206 bp, 5' TTATTCTTTTCTTGGCACTGATAAC and 3' AAATGGTGAATTGCCCTCGT for SARS-CoV-2 gRNA2 27401-27531 bp, and 5' GAGACTCGAGCTTGGTTCACCGCTCTCACT and 3' GAGAGGATCCTCGTCTGGTAGCTCTTCGGT for SARS-CoV-2 N gene. The levels of gRNA1, gRNA2 and N were normalized to those of housekeeping GAPDH according to the comparative cycle threshold method used for quantification, as recommended by the manufacture's protocol.

Statistical analyses

Data represent the means of at least 3 independent biological replicates ± standard deviation (SD). Data were statistically analyzed using Microsoft Excel (Microsoft, Redmond, WA) and the GraphPad Prism 10 software to determine significant differences (p-value <0.05) between groups using the paired two-tailed Student's *t* test and the two-way ANOVA followed by Tukey's honest significant difference test for multiple comparisons, respectively. The statistical significance (p-value <0.05) was highlighted with the symbol *. Images in the figures show representative experiments.

Curves activity assay

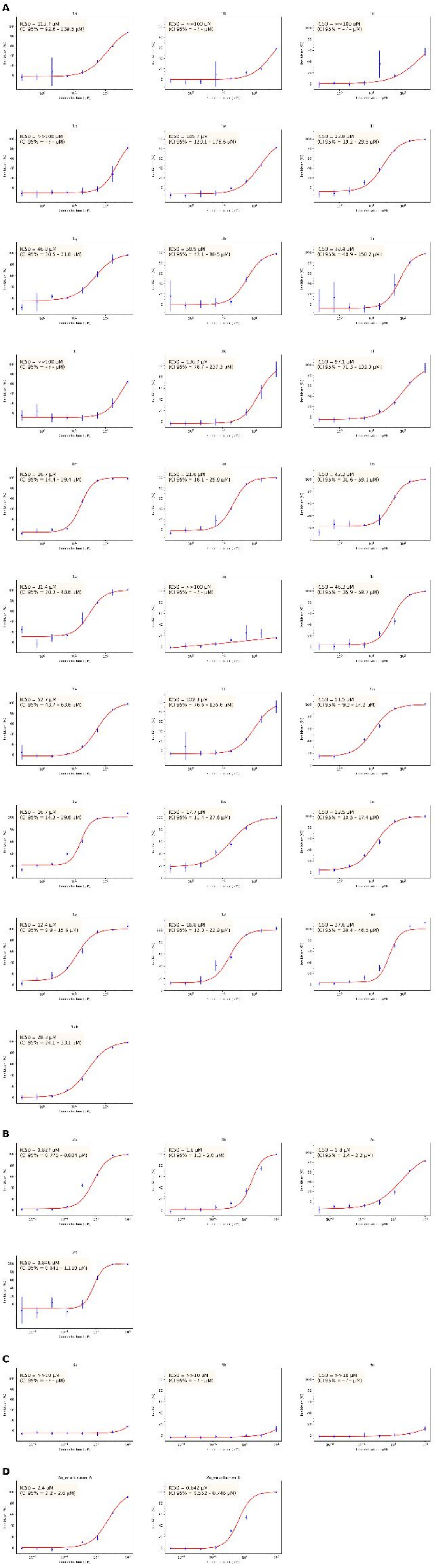

Figure S3. IC50s of synthesized tetrazoles. Dose-response curves for (A) non-covalent tetrazoles, (B) chloroacetamide containing tetrazoles, (C) cyanoacetamide containing tetrazoles and (D) chirally, separated enantiomers of tetrazole **2a**. IC50 values of SARS-CoV-2 3CLpro inhibition of cleavage activity in the presence of increasing concentrations of the measured compounds were determined by 3 parameter nonlinear regression.

References

(1) Patre, R. E.; Mal, S.; Nilkanth, P. R.; Ghorai, S. K.; Deshpande, S. H.; El Qacemi, M.; Smejkal, T.; Pal, S.; Manjunath, B. N. First Report on Bio-Catalytic N-Formylation of Amines Using Ethyl Formate. *Chem. Commun.* **2017**, *53* (15), 2382–2385. https://doi.org/10.1039/c6cc07679c.

(2) Dhake, K. P.; Tambade, P. J.; Singhal, R. S.; Bhanage, B. M. An Efficient, Catalyst- and Solvent-Free *N*-Formylation of Aromatic and Aliphatic Amines. *Green Chem. Lett. Rev.* **2011**, *4* (2), 151–157. https://doi.org/10.1080/17518253.2010.524168.

(3) Patil, P.; Ahmadian-Moghaddam, M.; Dömling, A. Isocyanide 2.0. *Green Chem.* **2020**, *22* (20), 6902–6911. https://doi.org/10.1039/d0gc02722g.

(4) Oerlemans, R.; Ruiz-Moreno, A. J.; Cong, Y.; others. Repurposing the HCV NS3–4A Protease Drug Boceprevir as COVID-19 Therapeutics. *RSC Med Chem* **2021**, *12*, 370–379. https://doi.org/10.1039/D0MD00367K.

(5) Sutanto, F.; Shaabani, S.; Oerlemans, R.; Eris, D.; Patil, P.; Hadian, M.; Wang, M.; Sharpe, M. E.; Groves, M. R.; Dömling, A. Combining High-Throughput Synthesis and High-Throughput Protein Crystallography for Accelerated Hit Identification. *Angew. Chem. Int. Ed.* **2021**, *60* (33), 18231–18239. https://doi.org/10.1002/anie.202105584.

(6) ASAP Discovery Consortium. *Target Enabling Packages (TEPs)*. ASAP Discovery Consortium. https://asapdiscovery.org/outputs/target-enabling-packages/ (accessed 2024-10-16).

(7) Douangamath, A.; Fearon, D.; Gehrtz, P.; Krojer, T.; Lukacik, P.; Owen, C. D.; Resnick, E.; Strain-Damerell, C.; Aimon, A.; Ábrányi-Balogh, P.; Brandão-Neto, J.; Carbery, A.; Davison, G.; Dias, A.; Downes, T. D.; Dunnett, L.; Fairhead, M.; Firth, J. D.; Jones, S. P.; Keeley, A.; Keserü, G. M.; Klein, H. F.; Martin, M. P.; Noble, M. E. M.; O'Brien, P.; Powell, A.; Reddi, R. N.; Skyner, R.; Snee, M.; Waring, M. J.; Wild, C.; London, N.; Von Delft, F.; Walsh, M. A. Crystallographic and Electrophilic Fragment Screening of the SARS-CoV-2 Main Protease. *Nat. Commun.* **2020**, *11* (1), 5047. https://doi.org/10.1038/s41467-020-18709-w.

(8) D'Arcy, A.; Bergfors, T.; Cowan-Jacob, S. W.; Marsh, M. Microseed Matrix Screening for Optimization in Protein Crystallization: What Have We Learned? *Acta Crystallogr. Sect. F Struct. Biol. Commun.* **2014**, *70* (9), 1117–1126. https://doi.org/10.1107/S2053230X14015507.

(9) Vonrhein, C.; Flensburg, C.; Keller, P.; Sharff, A.; Smart, O.; Paciorek, W.; Womack, T.; Bricogne, G. Data Processing and Analysis with the autoPROC Toolbox. *Acta Crystallogr. D* **2011**, *67* (4), 293–302. https://doi.org/10.1107/S0907444911007773.

(10) Kabsch, W. XDS. *Acta Crystallogr. D* **2010**, *66* (2), 125–132. https://doi.org/10.1107/S0907444909047337.

(11) Evans, P. R. Scaling and Assessment of Data Quality. *Acta Crystallogr. D* **2006**, *62* (1), 72–82. https://doi.org/10.1107/S0907444905036693

(12) Evans, P. R.; Murshudov, G. N. How Good Are My Data and What Is the Resolution? *Acta Crystallogr. D* **2013**, *69* (7), 1204–1214. https://doi.org/10.1107/S0907444913000061.

(13) Winn, M. D.; Ballard, C. C.; Cowtan, K. D.; Dodson, E. J.; Emsley, P.; Evans, P. R.; Keegan, R. M.; Krissinel, E. B.; Leslie, A. G. W.; McCoy, A.; McNicholas, S. J.; Murshudov, G. N.; Pannu, N. S.; Potterton, E. A.; Powell, H. R.; Read, R. J.; Vagin, A.; Wilson, K. S. Overview of the CCP4 Suite and Current Developments. *Acta Crystallogr. D* **2011**, *67* (4), 235–242. https://doi.org/10.1107/S0907444910045749

(14) Tickle, I. J.; Flensburg, C.; Keller, P.; Paciorek, W.; Sharff, A.; Vonrhein, C.; Bricogne, G. STARANISO; Global Phasing Ltd.: Cambridge, U.K., 2018–2022. https://staraniso.globalphasing.org

(15) Wojdyr, M.; Keegan, R.; Winter, G.; Ashton, A. DIMPLE: A Pipeline for the Rapid Generation of Difference Maps from Protein Crystals with Putatively Bound Ligands. *Acta Crystallogr. A* **2013**, *69* (s1), s299.

(16) Long, F.; Nicholls, R. A.; Emsley, P.; Gražulis, S.; Merkys, A.; Vaitkus, A.; Murshudov, G. N. ACEDRG: A Stereochemical Description Generator for Ligands. *Acta Crystallogr. D* **2017**, *73* (2), 112–122.

(17) Emsley, P.; Cowtan, K. Coot: Model-Building Tools for Molecular Graphics. *Acta Crystallogr. D* **2004**, *60* (12), 2126–2132. https://doi.org/10.1107/S0907444904019158

(18) Murshudov, G. N.; Skubák, P.; Lebedev, A. A.; Pannu, N. S.; Steiner, R. A.; Nicholls, R. A.; Winn, M. D.; Long, F.; Vagin, A. A. REFMAC5 for the Refinement of Macromolecular Crystal Structures. *Acta Crystallogr. D* **2011**, *67* (4), 355–367. https://doi.org/10.1107/S0907444911001314

(19) Liebschner, D.; Afonine, P. V.; Baker, M. L.; Bunkóczi, G.; Chen, V. B.; Croll, T. I.; Hintze, B.; Hung, L. W.; Jain, S.; McCoy, A. J.; Moriarty, N. W.; Oeffner, R. D.; Poon, B. K.; Prisant, M. G.; Read, R. J.; Richardson, J. S.; Richardson, D. C.; Sammito, M. D.; Sobolev, O. V.; Stockwell, D. H.; Terwilliger, T. C.; Urzhumtsev, A. G.; Videau, L. L.; Williams, C. J.; Adams, P. D. Macromolecular Structure Determination Using X-Rays, Neutrons and Electrons: Recent Developments in Phenix. *Acta Crystallogr. D* **2019**, *75* (10), 861–877. https://doi.org/10.1107/S2059798319011471

(20) Biacchesi, S.; Skiadopoulos, M. H.; Yang, L.; Murphy, B. R.; Collins, P. L.; Buchholz, U. J. Rapid Human Metapneumovirus Microneutralization Assay Based on Green Fluorescent Protein Expression. *J. Virol. Methods* **2005**, *128* (1-2), 192–197. https://doi.org/10.1016/j.jviromet.2005.05.005.
